# Granulocyte Macrophage-Colony Stimulating Factor Produces a Splenic Subset of Monocyte-Derived Dendritic Cells That Efficiently Polarize T Helper Type 2 Cells in Response to Blood-Borne Antigen

**DOI:** 10.3389/fimmu.2021.767037

**Published:** 2022-01-03

**Authors:** Seul Hye Ryu, Hyun Soo Shin, Hye Hyeon Eum, Ji Soo Park, Wanho Choi, Hye Young Na, Hyunju In, Tae-Gyun Kim, Sejung Park, Soomin Hwang, Moah Sohn, Eun-Do Kim, Kyoung Yul Seo, Hae-Ock Lee, Min-Geol Lee, Min Kyung Chu, Chae Gyu Park

**Affiliations:** ^1^ Laboratory of Immunology, Severance Biomedical Science Institute, Yonsei University College of Medicine, Seoul, South Korea; ^2^ Brain Korea 21 FOUR Project for Medical Science, Yonsei University College of Medicine, Seoul, South Korea; ^3^ Immune and Vascular Cell Network Research Center, National Creative Initiatives, Department of Life Sciences, Ewha Womans University, Seoul, South Korea; ^4^ Department of Biomedicine and Health Sciences, Graduate School, The Catholic University of Korea, Seoul, South Korea; ^5^ Department of Microbiology, College of Medicine, The Catholic University of Korea, Seoul, South Korea; ^6^ Department of Neurology, Severance Hospital, Yonsei University College of Medicine, Seoul, South Korea; ^7^ Department of Dermatology, Severance Hospital, Cutaneous Biology Research Institute, Yonsei University College of Medicine, Seoul, South Korea; ^8^ Department of Ophthalmology, Severance Hospital, Institute of Vision Research, Yonsei University College of Medicine, Seoul, South Korea; ^9^ Therapeutic Antibody Research Center, Genuv Inc., Seoul, South Korea; ^10^ Institute for Immunology and Immunological Diseases, Yonsei University College of Medicine, Seoul, South Korea

**Keywords:** allergic sensitization, dendritic cell, GM-CSF, GM-CSF receptor, monocyte-derived dendritic cell, spleen, T cell – DC interactions, Th2 cell

## Abstract

Dendritic cells (DCs) are key antigen-presenting cells that prime naive T cells and initiate adaptive immunity. Although the genetic deficiency and transgenic overexpression of granulocyte macrophage-colony stimulating factor (GM-CSF) signaling were reported to influence the homeostasis of DCs, the *in vivo* development of DC subsets following injection of GM-CSF has not been analyzed in detail. Among the treatment of mice with different hematopoietic cytokines, only GM-CSF generates a distinct subset of XCR1^-^33D1^-^ DCs which make up the majority of DCs in the spleen after three daily injections. These GM-CSF-induced DCs (GMiDCs) are distinguished from classical DCs (cDCs) in the spleen by their expression of CD115 and CD301b and by their superior ability to present blood-borne antigen and thus to stimulate CD4^+^ T cells. Unlike cDCs in the spleen, GMiDCs are exceptionally effective to polarize and expand T helper type 2 (Th2) cells and able to induce allergic sensitization in response to blood-borne antigen. Single-cell RNA sequencing analysis and adoptive cell transfer assay reveal the sequential differentiation of classical monocytes into pre-GMiDCs and GMiDCs. Interestingly, mixed bone marrow chimeric mice of *Csf2rb* ^+/+^ and *Csf2rb* ^-/-^ demonstrate that the generation of GMiDCs necessitates the *cis* expression of GM-CSF receptor. Besides the spleen, GMiDCs are generated in the CCR7-independent resident DCs of the LNs and in some peripheral tissues with GM-CSF treatment. Also, small but significant numbers of GMiDCs are generated in the spleen and other tissues during chronic allergic inflammation. Collectively, our present study identifies a splenic subset of CD115^hi^CD301b^+^ GMiDCs that possess a strong capacity to promote Th2 polarization and allergic sensitization against blood-borne antigen.

## Introduction

Dendritic cells (DCs) are critical antigen-presenting cells that can stimulate naive T cells (1). It has been recognized that classical DCs (cDCs) originate from cDC-committed precursors in the steady state, whereas DCs also derive from monocytes under inflammatory and infectious conditions (2–7). Our previous study (2) examined the development of monocyte-derived DCs (Mo-DCs) in lymphoid organs following the *in vivo* challenge with a series of ligands for toll-like receptors (TLRs). As a result, we discovered that significant numbers of Mo-DCs quickly mobilized to the lymph nodes (LNs), but not to the spleen, upon treatment with TLR4 ligands [i.e., lipopolysaccharide (LPS) and Gram-negative bacteria], but not with other TLR ligands (2). Then, we examined the role of hematopoietic cytokines related to the development of DC subsets and found that only granulocyte-macrophage colony stimulating factor (GM-CSF), but neither FMS-like tyrosine kinase 3 ligand (FLT3L) nor macrophage CSF (M-CSF), were able to induce the differentiation of DCs from splenocytes *in vitro* (8). Though the deletion of genes involved in GM-CSF signaling (9–11), the transgenic overexpression of GM-CSF (10, 12–14), and the treatment with polyethylene glycol-modified GM-CSF (15) were shown to influence the homeostasis of DCs, the *in vivo* development of DC subsets following injection of GM-CSF has not been scrutinized.

Allergen-specific CD4^+^ T helper type 2 (Th2) cells are pivotal players in causing and maintaining the allergic diseases as they specifically recognize the allergen and induce the subsequent development of allergic inflammation (16–18). DCs are essential antigen-presenting cells (APCs) that initiate immunity by stimulating and differentiating antigen-specific naive T cells (1, 19). Therefore, DCs play an important role in initiating and sustaining various immune-related diseases, such as allergies by priming Th2 cells to allergens (20, 21). Although type 2 cDCs (cDC2s) are shown to promote Th2 cell differentiation, cDC2s are also involved in the induction of other Th cells such as Th17 and follicular helper T cells (19, 22). Recently, cDC2s are further divided into anti-inflammatory and pro-inflammatory subsets in the steady state (23) and demonstrated to acquire the features of cDC1s and macrophages during inflammation and infection (24). The interaction between barrier epithelial cells and DCs is considered essential for the differentiation of Th2 cells and the initiation of allergies in the current paradigm of allergic sensitization. Allergen-stimulated barrier epithelial cells secrete pro-allergic cytokines and thus instruct DCs to polarize allergen-specific Th2 cells in the local draining LNs following activation and CCR7-dependent migration from the allergen-stimulated barrier tissues (21, 25–27). Recently, CD301b^+^ migratory DCs in the LNs are demonstrated to stimulate CD4^+^ T cells to polarize towards Th2 or Th17 cells (28, 29). However, the existence of a specialized subset of DCs in the spleen with a potent ability to systemically promote allergic sensitization has not been hypothesized previously.

Here, we set out to investigate the change of splenic DC subsets *in vivo* in response to three hematopoietic cytokines, i.e., GM-CSF, FLT3L, and M-CSF, and identified a novel CD115^hi^CD301b^+^ subset of DCs only after the treatment with GM-CSF. As compared to cDC subsets in the spleen, these GM-CSF-induced DCs (GMiDCs) were remarkably effective to polarize and expand Th2 cells against blood-borne antigen. Analysis of single-cell transcriptomics indicated that classical monocytes sequentially differentiated into pre-GMiDCs and GMiDCs in the spleen, which was verified by the adoptive transfer of classical monocytes. Interestingly, mixed bone marrow chimeric mice of *Csf2rb* ^+/+^ and *Csf2rb* ^-/-^ demonstrated that the generation of GMiDCs required the *cis* expression of GM-CSF receptor, which revealed a previously unrecognized essential role of GM-CSF in differentiation of Mo-DCs *in vivo*. Together, we discovered that a novel subset of CD115^hi^CD301b^+^ Mo-DCs mobilized to the spleen by treatment with GM-CSF and possessed a strong capacity to promote the differentiation of Th2 cells and the allergic sensitization against blood-borne antigen.

## Materials And Methods

### Mice

C57BL/6 mice were purchased from the Orient Bio (Seongnam, Korea) and C57BL/6-Tg(TcraTcrb)1100Mjb/J (OT-1), B6.Cg-Tg(TcraTcrb)425Cbn/J (OT-2), B6.SJL-*Ptprc^a^ Pepc^b^*/BoyJ (CD45.1), B6.129P2(C)-*Ccr7^tm1Rfor^* ^/^J (*Ccr7* KO), C57BL/6-*Ccr7^tm1.1Dnc^*/J (*Ccr7* ^gfp^), B6.129S1-*Csf2rb^tm1Cgb^*/J (GM-CSF receptor βc KO), B6.129S-*Csf2^tm1Mlg^*/J (GM-CSF KO), B6.129S4-*Ccr2^tm1Ifc^*/J (*Ccr2* KO), B6.129S6(C)-*Zbtb46^tm1.1Kmm^*/J (*Zbtb46*gfp), and B6.129S1-*Irf4^tm1Rdf^*/J (*Irf4* ^fl/fl^) mice from the Jackson Laboratory (Bar Harbor, ME). B6(FVB)-*Mgl2^tm1.1(HBEGF/EGFP)Aiwsk^*/J (*Mgl2*DTR) mice were a gift from Akiko Iwasaki (Yale University School of Medicine), B6.129S(C)-*Batf3^tm1Kmm^*/J (Batf3 KO) from Heung Kyu Lee (KAIST), and B6.Cg-Tg(Itgax-cre)1-1Reiz/J (*Itgax*-cre) from Hyoung-Pyo Kim (Yonsei University College of Medicine). Mice were bred and maintained at specific pathogen free facilities of the Department of Laboratory Animal Resources in the Yonsei University College of Medicine. All mice were used between 8 and 12 weeks of age. Experiments were performed with sex and age matched mice in accordance with the guidelines and protocols approved by the Institutional Animal Care and Use Committees of the Yonsei University College of Medicine (approval numbers 2016-0040, 2017-0001, 2019-0024, 2020-0003).

### Treatment With Hematopoietic Cytokines

Mice were subcutaneously injected once-daily with 10 μg of mouse M-CSF, FLT3L or GM-CSF. Cells were isolated and analyzed at 24 hours after the last injection of the respective cytokines. Mouse cytokines were provided from JW CreaGene (Seongnam, Korea) or produced in house as described previously (30–32).

### Cellular Preparation

Spleen, LNs, ear skin, intestine, and BM were harvested from mice after euthanizing with CO2, unless indicated otherwise. For the spleen, erythrocytes were lysed by RBC lysis buffer (BioLegend, San Diego, CA). Single cell suspensions of the spleen and LNs were prepared by grinding up with 100 μm cell strainers and syringe plungers. For the BM, tibia and femurs were flushed with DPBS (GE Healthcare Life Sciences, Logan, UT) and the extracted marrow was resuspended and filtered with a 100 μm cell strainer. For the preparation of single cell suspensions from the lamina propria, mesenteric lymph nodes and Peyer’s patches were removed from the intestine. Dissected intestines were placed in cold DMEM containing 5% fetal bovine serum (FBS, Gibco), opened longitudinally to remove fecal contents, and further incubated at 37°C in 25 ml of extraction media containing 1 mM EDTA and 1.4% FBS in DMEM with stirring at 250 rpm for 15 minutes. Then, intestines were washed with cold DMEM containing 5% FBS and intestinal mucus was removed by rolling over dry paper towel. After cut into small pieces, intestinal tissues were enzymatically digested with stirring in 25 ml of DMEM containing 1 mg/ml of collagenase IV (Gibco), 500 μg/ml of dispase II (Sigma-Aldrich), 50 μg/ml of DNase (Roche), and 1% FBS (Gibco) at 37°C for 30 minutes. Then, single cell suspension was passed through a 100 µm cell strainer and washed with cold DMEM containing 5% FBS (Gibco) twice. For the ear skin, ventral side and dorsal side of ear were separated mechanically. In DMEM containing 5% of FBS (Gibco) and 2 mg/ml of dispase II (Sigma), each side of the ear were floated and incubated in 37°C for 40 minutes. After incubation, epidermis was peeled off from dermis. Epidermis and dermis were chopped into 3 to 4 mm^2^ size and incubated in DMEM containing 5% FBS (Gibco), 1 mg/ml of collagenase D (Gibco) and 0.1 mg/ml of DNase I (Roche) at 37°C for 100 minutes, followed by passing through a 100 µm cell strainer. For the harvest of BALF, lung, and mediastinal LN preparation, mice were euthanized by intraperitoneal injection of a mixture of 6.25 mg tiletamine and zolazepam (Zoletil^®^ 50, Virbac, Carros, France) and 0.58 mg xylazine hydrochloride (Rompun^®^, Bayer, Leverkusen, Germany) per mouse. BALF was collected with 1 ml HBSS (Gibco) without calcium and magnesium. Before the harvest of lung tissues, mice were perfused gently with a syringe containing of HBSS. After mincing with a razor blade, lung tissues were incubated in 1 mg/ml collagenase type IV (Gibco) or collagenase D (Roche, Basel, Swiss) at 37°C for 40 minutes, followed by treatment in 20 mM EDTA for 5 minutes. Then, single cell suspensions were prepared by homogenizing lung tissues with 100 μm cell strainers and syringe plungers and subjected to lysis of erythrocytes with RBC lysis buffer, before wash and resuspension in HBSS (33).

### Flow Cytometry

Single cell suspensions were incubated in the culture supernatant of Fc receptor blocking 2.4G2 hybridoma cells for 20 minutes at 4°C followed by wash with FACS buffer composed of DPBS containing 2% FBS (Avantor, Radnor, PA), 0.1% sodium azide, and 2 mM EDTA. Then, cells were incubated with appropriate cocktails of fluorochrome- and/or biotin-conjugated monoclonal antibodies (**Supplementary Table 1**) in 96-well v-bottom plate at 4°C for 30 minutes. For intracellular staining, cells were first stimulated with PMA (12 nM), ionomycin (1 μM), and Brefeldin A (5 μg/ml) at 37°C for 4 hours. Then, the stimulated cells were incubated with 2.4G2 and conjugated monoclonal antibodies for surface staining as above, followed by fixation, permeabilization, and intracellular staining with conjugated monoclonal antibodies according to the manufacturer’s instructions (Fixation buffer/Intracellular staining permeabilization wash buffer, BioLegend). Multiparameter analysis of each sample was performed on FACSVerse™ and LSRFortessa™ flow cytometers (BD Biosciences, San Jose, CA) and flow cytometric isolation of cells was performed on a BD FACSAria™ II cell sorter (BD Biosciences) at the Flow Cytometry Core Facility of the Yonsei University College of Medicine. Collected data were analyzed with FlowJo software (BD Biosciences).

### Microscopic Analysis

To visualize the morphology of individual cells, splenocytes were sorted on a BD FACSAria™ II cell sorter through 85 μm nozzle. Each sorted population of 1 to 2×10^5^ cells was cultured in DMC7 medium (30) composed of DMEM containing L-glutamine, high glucose, and pyruvate (GE Healthcare Life Sciences) supplemented with 7% FBS, 1× non-essential amino acids (GE Healthcare Life Sciences), and 1× antibiotic-antimycotic (GE Healthcare Life Sciences) on a well of 96-well flat-bottom cell culture plate overnight. Then, cellular morphology was analyzed by eclipse TS100 (Nikon, Tokyo, Japan) and IX73 (Olympus, Tokyo, Japan) fluorescent inverted microscopes. For histological staining, fresh tissues were embedded in optimum cutting temperature (OCT) compound (Sakura Finetek USA, Torrance, CA) and were frozen on dry ice with 2-methylbutane. Then, 10 μm thick sections were cut on a cryostat and collected on microslides (Muto Pure Chemicals, Tokyo, Japan). Each section was fixed in absolute acetone for 15 minutes and then allowed to dry for at least 5 minutes. Endogenous peroxidases were blocked by immersing tissue sections into 0.3% hydrogen peroxide solution. Before staining, slides were blocked with 10% normal goat serum for 2 hours and M.O.M.^®^ (Mouse on Mouse) blocking reagent (Vector Laboratories, Burlingame, CA) at room temperature for 1 hour. Primary antibody staining was performed at room temperature for 2 hours or at 4°C overnight, followed by staining with fluorochrome-conjugated antibodies at room temperature for 1 hour. For some cases, after staining sections with horseradish peroxidase (HRP)-conjugated antibody, tyramide signal amplification (TSA) kit (Thermo Fisher Scientific) was used according to the manufacturer’s instructions, and then the sections were stained with fluorochrome-conjugated antibodies at room temperature for 2 hours. After mounting with Dako fluorescence mounting medium (Dako, Santa Clara, CA), images were acquired with an LSM700 confocal microscope (Zeiss, Oberkochen, Germany) at the Yonsei Advanced Imaging Center in cooperation with Carl Zeiss Microscopy of the Yonsei University College of Medicine.

### T Cell Proliferation Assay

APCs were prepared from the spleens at 1 hour after the intravenous injections of 0.5 to 3.5 mg ovalbumin (OVA, Grade V, Sigma-Aldrich, St. Louis, MO). Single cell suspensions were prepared from the respective tissues as described above, stained with suitable antibodies, and sorted according to the appropriate gating strategies. Splenocytes from OT-1 or OT-2 transgenic mice were enriched for naive T cells by magnetic depletion using a mixture of biotinylated antibodies against CD19, CD11b, NK1.1, CD25, CD44, F4/80, MHC II, and CD4 (for CD8^+^ OT-1) or CD8 (for CD4^+^ OT-2) and Dynabeads™ Biotin Binder (Thermo Fisher Scientific). Enriched T cells were labeled with 5 mM reagent from CellTrace™ CFSE cell proliferation kit (Thermo Fisher Scientific) or CellTrace™ Violet cell proliferation kit (Thermo Fisher Scientific) at 37°C for 10 minutes, washed, and counted. Enriched and labeled 5×10^4^ naive T cells and graded doses of purified APCs were co-cultured in 96-well round-bottom tissue culture plates with DMC7 medium supplemented with 57.2 μM 2-mercaptoethanol (Sigma-Aldrich). Proliferated T cells were assessed by flow cytometry at 3 to 5 days after culture (34).

### Th Cell Polarization Assay

OT-2 T cells and OVA-loaded APCs were prepared as above. Naive OT-2 T cells (5×10^4^) and purified OVA-loaded antigen-presenting cells (1×10^4^) were co-cultured in 96-well round-bottom tissue culture plates under the control (medium alone) or Th polarizing conditions (23) as follows: Th1 with 10 ng/ml IL-12 (BioLegend) and 5 μg/ml anti-IL-4 (BioLegend); Th2 with 10 ng/ml IL-2 (BioLegend), 10 ng/ml IL-4 (BioLegend), and 5 μg/ml anti-IFN-γ (BioLegend); Th17: 2 ng/ml TGF-β (Peprotech, Rocky Hill, NJ), 10 ng/ml IL-1β (BioLegend), 20 ng/ml IL-6 (BioLegend), 5 μg/ml anti-IFN-γ, and 5 μg/ml anti-IL-4. Intracellular staining of Th cytokines IFN-γ (Th1), IL-4 (Th2), and IL-17A (Th17) were performed by flow cytometry following stimulation with PMA, ionomycin, and brefeldin A at 3 to 5 days after culture.

### Antigen Uptake Assay

Mice were sacrificed 1 hour after the intravenous injection of 200 μg FITC-OVA (Thermo Fisher Scientific). Then, single suspension of the spleen was prepared and stained with appropriate cocktails of fluorochrome-conjugated monoclonal antibodies for flow cytometric analysis.

### RNA Sequencing Analysis

Splenocytes were stained and sorted according to the suitable gating strategy from steady-state or GM-CSF-treated mice. For each population, total RNA was extracted by MiniBEST universal RNA extraction kit (TaKaRa Bio, Shiga, Japan) from at least 1×10^5^ sorted cells. Subsequent RNA-sequencing (RNA-seq) procedures were carried out by Macrogen (Seoul, Korea) as follows. Reverse transcription of mRNA and generation of cDNA libraries were carried out with SMARTer Ultra low input RNA library kit and sequenced with Illumina NovaSeq. The raw reads from the sequencer were preprocessed to remove low quality and adapter sequence before analysis to align the processed reads to the *Mus musculus (mm10)* using HISAT v2.1.0 (35). HISAT utilized two types of indexes for alignment (a global, whole-genome index and tens of thousands of small local indexes). These two types’ indexes were constructed using the same BWT (Burrows–Wheeler transform) a graph FM index (GFM) as Bowtie2 (36). After alignment, StringTie v1.3.4d (37, 38) was used to provide the relative abundance estimates as read count values of transcript and gene expressed in each sample and transcript assembly of known transcripts, novel transcripts, and alternative splicing transcripts. Multidimensional scaling method was used to visualize the similarities among samples. The larger the dissimilarity between two samples, the further apart the points representing the experiments in the picture should be. We applied to the Euclidean distance as the measure of the dissimilarity. Hierarchical clustering analysis also was performed using complete linkage and Euclidean distance as a measure of similarity to display the expression patterns of differentially expressed transcripts which are satisfied with |fold change| ≥ 2. All data analysis and visualization of differentially expressed genes was conducted using R 3.5.1 (https://www.r-project.org). Hierarchical clustering was performed with Morpheus (https://software.broadinstitute.org/morpheus/) and Venn diagram analysis was performed in (http://www.interactivenn.net/) (39). Ternary plot analysis was performed using ‘ggtern’ (40).

### Adoptive Cell Transfer

Single cell suspensions of splenocytes or BM from steady-state or GM-CSF-treated mice were labeled with CFSE followed by flow cytometric sorting. Each population of 1 to 2×10^6^ cells was intravenously injected into each recipient treated subcutaneously with GM-CSF. In the case of T cell proliferation *in vivo*, mice were treated with 3 once-daily injections of control DPBS or GM-CSF, followed by the adoptive transfer of CFSE-labeled naive CD4^+^CD25^-^CD44^-^ T cells and the intravenous injection of 100 μg OVA. For the depletion of GMiDCs, 500 ng of diphtheria toxin (DT, Sigma-Aldrich) was injected intraperitoneally to *Mgl2* ^+/+^ and *Mgl2* ^DTR/+^ mice in parallel with the subcutaneous injection of 10 μg GM-CSF. For allergic airway inflammation, donor mice were treated with 3 once-daily subcutaneous injections of 10 μg GM-CSF prior to injecting 500 μg of soluble OVA intravenous for 1 hour and harvesting the spleen. Flow cytometrically sorted subsets of 5×10^5^ splenocytes were adoptively transferred intravenously into recipient mice treated with one subcutaneous injection of 10 μg GM-CSF.

### Allergic Airway Inflammation

C57BL/6 mice were sensitized by one intravenous injection of 50 or 100 μg soluble OVA with or without once-daily subcutaneous injection of 10 μg GM-CSF for 3 or 5 days. From 10 days after sensitization, the mice were intranasally challenged with 50 or 100 µg of OVA once-daily for five days. The mice were sacrificed for analysis at 24 hours after the final intranasal challenge. For the depletion of GMiDCs during sensitization, 500 ng of DT (Sigma-Aldrich) was injected intraperitoneally to *Mgl2* ^+/+^ and *Mgl2* ^DTR/+^ mice in parallel with subcutaneous injection of 10 μg GM-CSF. For *Alternaria*-induced allergic airway inflammation, C57BL/6 mice were intranasally treated once-daily with 5 µg protein of *Alternaria* extract (Greer, Cambridge, MA) for up to 12 days. The mice were sacrificed for analysis at 24 hours after the final intranasal treatment with *Alternaria* extract.

### Mixed BM Chimeric Mice

CD45.1 *Csf2rb* ^+/+^ or CD45.2 *Csf2rb* ^-/-^ recipient mice were lethally irradiated (8 Gy total body irradiation twice in four hours interval) and adoptively transferred intravenously with 6×10^6^ cells containing an equal mixture of BM cells from CD45.1 *Csf2rb* ^+/+^ and CD45.2 *Csf2rb* ^-/-^ mice. Mice were maintained with supply of water containing antibiotics for at least 6 weeks before the experiments started.

### Generation and Processing of CITE-Seq Data

FACS sorted CD11b^+^ splenocytes were loaded into the Chromium system (10x Genomics, Pleasanton, CA) targeting 7,000 cells per sample. The cDNA libraries for mRNA were generated using Chromium Single Cell 3’ v3 Reagent Kits according to the manufacturer’s instructions. Following the CITE-seq protocol (41), ADT PCR additive primers were added to cDNA PCR and the ADT libraries were generated separately from the mRNA’s. Constructed mRNA and ADT libraries were sequenced together on a HiSeq2500 system (Illumina) targeting 15,000 cDNA reads and 5,000 ADT reads per cell using the 100 base-pair paired end mode. CITE-seq data of the mRNA fraction and the ADT fraction were processed using the CellRanger-3.0.2 pipeline (10x Genomics) with a mouse reference genome (*mm10*) and the CITE-seq-Count-1.4.2, respectively. The filtered gene expression matrix from the CellRanger and the ADT count matrix from the CITE-seq-Count were analyzed together by the ‘Seurat’ R package v3.6.0 (42). Before the downstream analysis, we excluded low quality cells with nFeature_RNA < 200, nFeature_RNA > 5,000, or mitochondrial gene expression > 15%. For the remaining cells and genes, the UMI count matrix was log-normalized and then the variable 2,000 genes were selected using ‘vst’ method in each sample. Using these variable genes, control and GM-CSF-treated data were integrated by anchor-based method in Seurat v3.

### CITE-Seq Data Analysis

Integrated data was scaled across total 11,631 cells and then PCA, clustering, and UMAP analyses were performed. Differentially expressed genes (DEGs) of each cluster were selected by the Seurat FindAllMarkers function. The cell types were defined by cluster-based cell type marker gene expression. After excluding clusters for the lineage cell types of T cells, B cells, granulocytes, neutrophils, and cDCs, the remaining clusters 1, 2, 4, and 5 which show assorted features of monocytes, macrophages, and dendritic cells and cluster 6 expressing the progenitor DC markers were named MoMacDC or Focus bundle. A total 6,075 cells of Focus bundle were re-scaled, and further analyses including PCA, clustering, and UMAP were performed. Cell cycle phase scores (S.score and G2M.score) of each cell were evaluated using the Seurat CellCycleScoring function. The ‘cc.genes’ from the Seurat package consisting of human S phase markers and G2/M phase markers was converted into mouse homolog genes. The 38 of 43 S phase markers and 51 of 54 G2/M phase markers were used in the analysis (https://github.com/satijalab/seurat/issues/462). In single cell sequencing data, Clusters I, II, III, and IV were identified by *in-silico* gating based on ADT expression mirroring the FACS gating strategy. The ADT count matrix was CLR-normalized and scaled before gating. To compare CD115^hi^ cells with cDC2 and macrophages, cDC2 and macrophages were collected from those other than Focus bundle cells in the scRNA-seq data. A total of 92 cDC2 cells and 29 macrophages were identified by CD11c^+^ MHC II^+^ 33D1^+^ CD172a^+^ (relative expression value of *Itgax* > 0, *H2-Aa* > 2.5, *Clec4a4* > 0, and *Sirpa* > 0) and F4/80^+^ C1q^+^ (*Adgre1* > 1.5 and *C1qa* > 2), respectively. In **Supplementary Figure 27**, the cells with a relative expression value of *Cd209a* > 0 were defined as CD209a^+^. Signature genes for Th2 polarization (43–45) and antigen uptake (43) were compared.

### Pseudotime and RNA Velocity of Focus Bundle

We utilized the pseudotime (46) and RNA velocity (47) to show the differentiation paths and changes in cellular state of Focus bundle cells. Pseudotime of Focus bundle cells was estimated by the R package ‘Monocle3’ v0.2.1. Trajectory graph for pseudotime estimation was learned from the UMAP embeddings of RNA-seq expression data, and the other steps were processed with the default option. Based-on the RNA expression analysis, we assigned the progenitor-like cells (cluster 9) as the starting point for the pseudotime. Following the velocyto pipeline (47), we estimated the RNA velocity in each cell. While running ‘velocyto run’ function in velocyto.py, a barcode list of the Focus bundle cells was applied with -b option, and the mm10-rmsk.gtf (Dec. 2011 version) downloaded from UCSC genome browser (https://genome.ucsc.edu/) was used with -*m* option to mask expressed repetitive elements. Counted spliced, unspliced, and ambiguous matrices were loaded into R and converted to the Seurat object using R package ‘Seurat’ and ‘SeuratWrappers’. We merged control and GM-treated data and then performed SCTransform in Seurat v3 using the spliced matrix to remove batch effect before running PCA and UMAP. RunVelocity function in R package ‘velocyto.R (version 0.6)’ was used to calculate the velocity of each cell. We set deltaT = 1, kCells = 25, and fit.quantile = 0.02 as options for RunVelocity. The velocity values were projected onto the UMAP embedding from the RNA-seq analysis.

### Statistical Analysis

Data in all experiments were analyzed with one-way or two-way ANOVA and Student’s *t*-test. Statistical analysis was performed with Prism (GraphPad Software, La Jolla, CA).

### Data Availability

All the sequencing data generated during the course of our study have been deposited and are available at the Gene Expression Omnibus (GEO) database under the accession numbers GSE157945 and GSE158200. All other data and materials supporting the findings in this report are available from the corresponding author upon request. Source data are provided with this paper.

## Results

### GM-CSF Treatment Generates a Novel Subset of Splenocytes in the DC Gate

**Figure 1:**
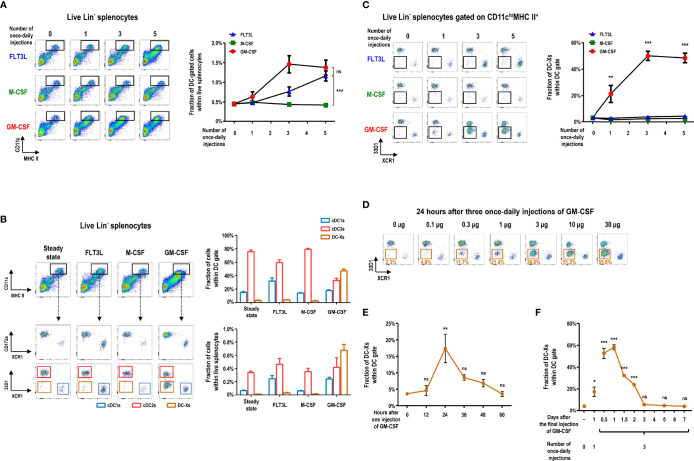
GM-CSF treatment generates a new subset of DCs in the spleen. **(A)** Changes in lineage (Lin)^-^ splenocytes within CD11c^hi^MHC II^+^ DC gate are analyzed at 24 hours after different numbers of once-daily injections of 10 μg of the respective cytokines. Lin^-^ cells exclude CD3^+^, CD19^+^, Ly6G^+^, and TER119^+^ cells. Data are shown in mean ± SEM (n = 3 to 7 mice for each condition). **(B)** Fractions of Lin^-^ splenocyte subsets within DC gate at 24 hours after 3 once-daily injections of the respective cytokines. Data are shown in mean ± SEM (n = 7 mice for each condition). **(C)** Changes in the fraction of splenocytes within XCR1^-^33D1^-^ DC-X gate at 24 hours after different numbers of once-daily injections of the respective cytokines. Data are shown in mean ± SEM (n = 3 to 7 mice for each condition). **(D)** Fraction of splenic DC-Xs within DC gate at 24 hours after 3 once-daily injections of graded doses of GM-CSF. Representative flow cytograms (n = 2 mice for each condition). **(E, F)** Kinetic changes of splenic DC-Xs after 1 injection **(E)** and 3 once-daily injections **(F)** of 10 μg of GM-CSF. Data are shown in mean ± SEM (n = 3 mice for each condition). *p ≤ 0.05; **p ≤ 0.01; ***p ≤ 0.001; ns, not significant; one-way **(E, F)** or two-way **(A, C)** ANOVA test.

We investigated the change of splenic DC subsets following injection of 3 different hematopoietic cytokines (i.e., FLT3L, M-CSF, or GM-CSF). In the steady state, splenic DCs exist as two main subsets, cDC1s and cDC2s, primarily defined by surface markers (6, 48, 49). Key surface markers for splenic cDC1s include CD8α, CD205, and XCR1; those for splenic cDC2s are CD172a, 33D1 (DCIR2 or Clec4A4), and CD11b (**Supplementary Figure 1**). Once-daily injections of GM-CSF increased splenocyte numbers in the CD11c^hi^MHC II^+^ DCs gate more quickly than those of FLT3L, whereas M-CSF treatment caused no change (**Figure 1A**). Among the C57BL/6 mice injected once daily with 3 different cytokines, only those treated with GM-CSF produced a new subset of XCR1^-^33D1^-^ splenocytes within the splenic DC gate (**Figures 1B, C**, and **Supplementary Figures 1, 2**). These XCR1^-^33D1^-^ DCs were tentatively named as DC-Xs in comparison to XCR1^+^33D1^-^ cDC1s and XCR1^-^33D1^+^ cDC2s. Evidently, 3 once-daily injections of more than 10 µg of GM-CSF per mouse fully mobilized DC-Xs to become the dominant DCs in the spleen (**Figure 1D**). After one injection of GM-CSF, DC-Xs accumulated to peak at 24 hours and quickly vanished in 36 to 48 hours (**Figure 1E** and **Supplementary Figure 3A**). Meanwhile, the largely accumulated DC-Xs similarly disappeared within 3 days following cessation of the 3 once-daily injections of 10 µg GM-CSF (**Figure 1F** and **Supplementary Figure 3B**).

### GM-CSF-Induced XCR1^-^33D1^-^ DCs in the Spleen Are Superior to Stimulate CD4^+^ T Cells in Response to Blood-Borne Antigen

**Figure 2:**
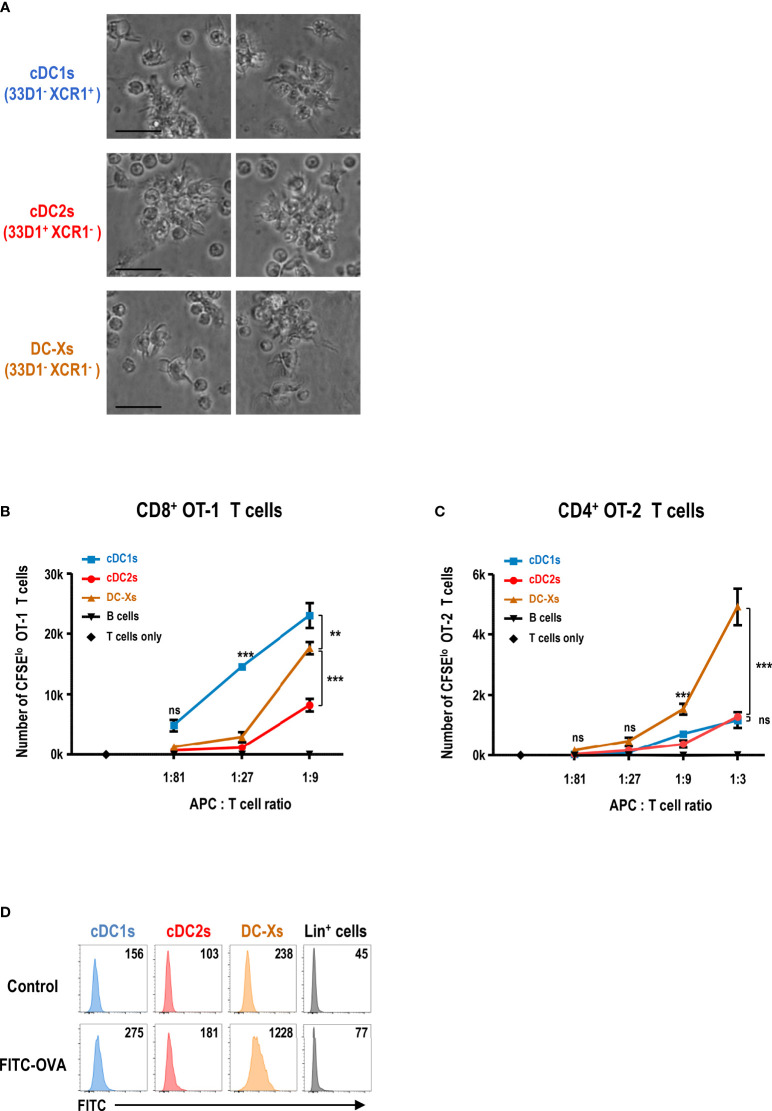
GM-CSF-induced splenic DCs efficiently stimulate CD4^+^ T cells in response to blood-borne antigen. **(A)** Morphologies of splenic DC subsets isolated from GM-CSF-treated mice and cultured overnight (magnification, 400×; scale bars, 25 μm). Representative images (n = 2 mice). **(B, C)** Antigen presentation by splenic DC subsets and CD19^+^ B cells isolated from GM-CSF-treated mice at 1 hour after the intravenous injection of OVA. Proliferation of CFSE-labeled naive CD8^+^ OT-1 **(B)** and CD4^+^ OT-2 **(C)** T cells following culture with graded doses of different antigen-presenting cells (APCs). Data are shown in mean ± SEM (n = 3 or 4 replicates) from 2 **(B)** and 4 **(C)** independent experiments. **(D)** Uptake of blood-borne FITC-OVA by splenic DC subsets at 1 hour after the intravenous injection of control DPBS or FITC-OVA into GM-CSF-treated mice. Mean fluorescence intensity (MFI) of FITC signal is denoted. Lin^+^ cells include CD3^+^, CD19^+^, Ly6G^+^, and TER119^+^ cells. Representative flow cytograms (n = 3 mice for each condition). **p ≤ 0.01; ***p ≤ 0.001; ns, not significant; two-way ANOVA test **(B, C)**.

We isolated each subset of DCs from the spleen of GM-CSF-treated mice and verified that they all exhibited non-adherent clustering and probing morphology of typical DCs (**Figure 2A**). To evaluate the functional capacity of DCs to prime and expand naive T cells, ovalbumin (OVA)-specific TCR transgenic T cells (i.e., CD8^+^ OT-1 and CD4^+^ OT-2) were used as responders; splenic cDCs and DC-Xs were sorted from the GM-CSF-treated mice and compared. In the co-cultures of graded doses of each splenic DC subset with OVA and naive OT-1 or OT-2 T cells, DC-Xs were inferior to cDC1s and/or cDC2s such that cDC1s were superior APCs for OT-1 T cells and cDC2s for OT-2 T cells (**Supplementary Figures 4A, B**).

Because the spleen is the body’s largest secondary lymphoid organ and filter for blood-borne antigens (49, 50), we administered soluble OVA intravenously to the GM-CSF-treated mice. After 1 hour, we isolated splenic DC subsets and co-cultured with naive OT-1 or OT-2 T cells. Surprisingly, when the blood-borne antigen was delivered to the spleen, DC-Xs were far superior to cDC1s and cDC2s in stimulating naive OT-2 T cells (**Figure 2C** and **Supplementary Figure 5B**). Besides, the ability of DC-Xs to stimulate naive OT-1 T cells also improved significantly, although cDC1s were still better APCs for OT-1 T cells (**Figure 2B** and **Supplementary Figure 5A**). Next, we assessed the capacity of each splenic DC subset to take up blood-borne antigens by injecting fluorescein isothiocyanate-labeled OVA (FITC-OVA) intravenously. The result showed that DC-Xs were clearly superior to cDC1s and cDC2s in taking up blood-borne FITC-OVA (**Figure 2D**), suggesting that the greater ability of DC-Xs to present blood-borne OVA to OT-2 T cells was in part due to their superior capacity to take up blood-borne antigens.

### GM-CSF-Induced DCs Are a Subset of Splenocytes Expressing High Levels of CD115

**Figure 3:**
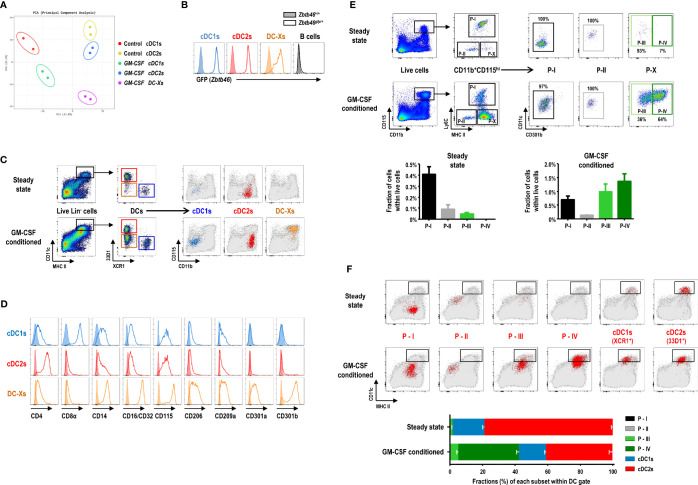
GM-CSF-induced splenic DCs express high levels of CD115. **(A)** Principal components analysis (PCA) of transcriptomes of splenic DC subsets in steady-state (control) and GM-CSF-treated mice (n = 2 mice for each cell population). **(B)** Flow cytograms showing GFP levels, as a reporter for *Zbtb46* gene expression, in splenic DC subsets and CD19^+^ B cells by comparing between *Zbtb46* ^gfp/+^ (line histograms) and *Zbtb46* ^+/+^ (shaded histograms) mice treated with GM-CSF. Representative flow cytograms (n = 3 mice for each genotype). **(C)** Gating strategy for each population of splenic DC subsets in control and GM-CSF-treated mice, backgated in a CD11b versus CD115 dot plot of total live-gated cells (gray dots). cDC1s represented by blue dots; cDC2s by red dots; DC-Xs by orange dots. Representative flow cytograms (n = 100 or more mice for each condition). **(D)** Flow cytograms showing the expression of the respective molecules (line histograms) on the surface of splenic DC subsets in GM-CSF-treated mice. Shaded histograms represent fluorescence minus one (FMO) control samples. Representative flow cytograms (n = 2 mice for each condition). **(E)** Gating strategy (upper panels) identifying GM-CSF-induced DC population in the spleen. Fractions (lower graphs) of 4 splenic CD11b^+^CD115^hi^ cell populations, i.e., P-I, P-II, P-III, and P-IV in control and GM-CSF-treated mice. Data are shown in mean ± SEM (n = 3 mice for each condition). **(F)** Each population (red dots) of splenic DC and CD11b^+^CD115^hi^ cell subsets in control and GM-CSF-treated mice is backgated in an MHC II versus CD11c dot plot of total live-gated cells (gray dots). Fraction (lower graph) of each cell subset within the DC gate on flow cytograms (upper panels). Data are shown in mean ± SEM (n = 3 mice for each condition).

We compared the global gene expression profiles of bulk DC subsets sorted from the spleens of control and GM-CSF-conditioned mice. Principal components analysis (PCA) of the transcriptomes indicated that the clusters of cDC1s were located close to each other; so were those of cDC2s (**Figure 3A**). Meanwhile, DC-Xs clustered between cDC1s and cDC2s from the spleen of GM-CSF-treated mice. Hierarchical cluster analysis (HCA) of the transcriptomes also resulted in similar outcomes (**Supplementary Figure 6A**). The expression of various transcription factor genes associated with DC development, such as *Batf3*, *Id2*, *Irf4*, *Irf8*, *Spi1* (PU.1), *Zbtb46*, etc., were augmented in DC-Xs as similarly as in cDC1s and/or cDC2s, while a number of genes related to monocytes and/or Mo-DCs, such as *Cd14*, *Fcgr3* (CD16), *Fcgr2b* (CD32), *Csf1r* (CD115), *Mrc1* (CD206), *Cd209a*, *Clec10a* (CD301a), *Mgl2* (CD301b), etc., were also upregulated in DC-Xs (**Supplementary Figures 6B, C**), implying that DC-Xs are Mo-DCs. Indeed, the high expression of DC-specific transcription factor *Zbtb46* (51) was similarly detected from cDC1s, cDC2s, and DC-Xs in the *Zbtb46* ^gfp/+^ mice treated with GM-CSF (**Figure 3B**), indicating that DC-Xs belong to the DC lineage.

We next characterized the surface markers of DC-Xs distinguished from those of cDCs by flow cytometry (**Supplementary Figure 7**) and found the conspicuously elevated expression of CD115 and CD301b on the surface of DC-Xs (**Figures 3C, D**). Accordingly, we devised new gating strategies for splenocytes expressing high levels of CD115 (**Figure 3E** and **Supplementary Figure 8**). In the steady state, CD11b^+^CD115^hi^ splenocytes were divided into 3 subpopulations (Ly6C^+^ **P-I**, Ly6C^-^MHC II^-^ **P-II**, and Ly6C^-^MHC II^+^CD301b^-^ **P-III**), two of which are monocyte subsets (i.e., P-I and P-II). In the mice treated with GM-CSF, there existed 4 subpopulations in CD11b^+^CD115^hi^ splenocytes (P-I, P-II, P-III, and Ly6C^-^MHC II^+^CD301b^+^ **P-IV**), among which the most populous P-IV subset was dominant in the DC gate (**Figures 3E, F**). Therefore, DC-Xs consist mainly of P-IV subset with a minor contribution of P-III. Meanwhile, only a small subset of cDC2s expressed CD301b, as previously reported (23), with/without the treatment of GM-CSF (**Supplementary Figure 9**).

### CD115^hi^CD301b^+^ Cells Are GM-CSF-Induced DCs (GMiDCs) in the Spleen

**Figure 4:**
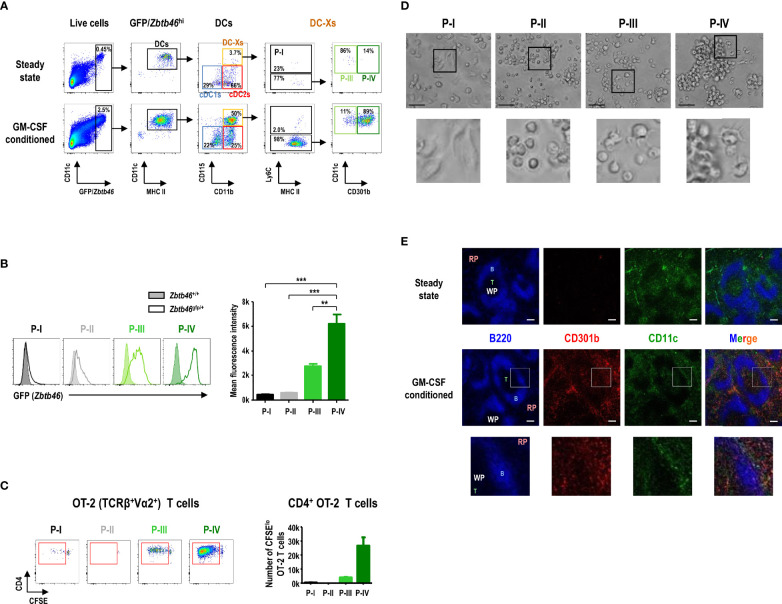
CD115^hi^CD301b^+^ splenocytes are GM-CSF-induced DCs (GMiDCs). **(A)** Gating strategy showing the distribution of GFP/*Zbtb46* ^hi^ splenocytes within the respective DC and CD11b^+^CD115^hi^ cell subsets in control and GM-CSF-treated *Zbtb46* ^gfp/+^ mice. Representative flow cytograms (n = 2 mice for each condition). **(B)** Expression level of GFP (i.e., *Zbtb46* gene) in splenic CD11b^+^CD115^hi^ cell subsets by comparing between *Zbtb46* ^gfp/+^ (line histograms) and *Zbtb46* ^+/+^ (shaded histograms) mice treated with GM-CSF. Data are shown in mean ± SEM of MFI for GFP signal in each cell subset from GM-CSF-treated *Zbtb46* ^gfp/+^ mice (n = 3 mice). **p ≤ 0.01; ***p ≤ 0.001; one-way ANOVA test. **(C)** Proliferation of CFSE-labeled naive CD4^+^ OT-2 T cells after co-culture (at an APC to T cell ratio of 1:5) with CD11b^+^CD115^hi^ cell subsets isolated from the spleen of GM-CSF-treated mice loaded with blood-borne OVA for 1 hour. Data are shown in mean ± SEM (n = 3 replicates for P-I, P-III, and P-IV; n = 1 for P-II) from 2 independent experiments. **(D)** Morphologies of splenic CD11b^+^CD115^hi^ cell subsets isolated from GM-CSF-treated mice and cultured overnight (magnification, 400×; scale bars, 25 μm). Representative images (n = 2 mice). **(E)** Spleen sections from control (upper panels) and GM-CSF-treated (lower panels) mice are stained for B cells with anti-B220 (blue), GMiDCs with anti-CD301b (red), and total DCs with anti-CD11c (green). RP, red pulp; WP, white pulp; B, B cell area; T, T cell area; magnification, 100×; scale bars, 100 μm. Insets are digitally magnified below to allow better visualization of morphology **(D)** and localization **(E)**.

Analysis of *Zbtb46* expression, a DC-specific transcription factor, in splenocytes using the *Zbtb46* ^gfp/+^ mice demonstrated that only a few of the cells in the green fluorescent protein (GFP)^hi^ population were found in the CD115^hi^ subsets in the steady state (**Figure 4A**). In contrast, the number of CD115^hi^ cells in the GFP^hi^ population greatly increased in the *Zbtb46* ^gfp/+^ mice following treatment with GM-CSF, and most of the CD115^hi^GFP^hi^ cells belonged to the P-IV subset (**Figures 4A, B**). Because CD11b^+^CD115^hi^ DC-Xs were far superior to cDC subsets in presenting blood-borne OVA to naive CD4^+^ OT-2 T cells, we evaluated which subset(s) of CD11b^+^CD115^hi^ splenocytes were responsible. Only P-IV cells were able to induce strong proliferation of naive OT-2 T cells in response to blood-borne OVA, whereas the other CD11b^+^CD115^hi^ cell subsets were either incompetent or weak to stimulate naive OT-2 T cells (**Figure 4C**). P-IV cells expressed a high level of *Zbtb46* whereas P-I and P-II cells as well as F4/80^+^ red pulp macrophages expressed low levels of *Zbtb46* (**Supplementary Figure 10A**). Regarding capacity to capture blood-borne FITC-OVA antigen, both P-III and P-IV cells exhibited superior to P-I and P-II cells but inferior to F4/80^+^ red pulp macrophages (**Supplementary Figure 10B**). Collectively, these data indicate that P-IV cells are the **GM**-CSF-**i**nduced **DCs** (hereafter **GMiDCs**) in the spleen.

In the overnight culture of splenocytes isolated from GM-CSF-treated mice (**Figure 4D**), the individual morphologies of CD11b^+^CD115^hi^ cell subsets revealed that P-I cells, defined as classical monocytes, became adherent macrophage-like cells while P-II cells, non-classical monocytes, displayed a round-shaped morphology; P-IV cells, GMiDCs, contained the large clusters of non-adherent cells with DC morphology, whereas only a fraction of P-III cells exhibited DC morphology without cell clusters. In the tissue sections, CD301b^+^ GMiDCs were found in the T cell areas of the white pulp (WP) as well as in the red pulp (RP) of the spleen from GM-CSF-conditioned mice, but not from steady-state control mice (**Figure 4E**). GMiDCs existed more abundantly in the RP than in the WP, while GMiDCs and cDCs in the WP were localized distinct from each other (**Figure 4E** and **Supplementary Figure 11**). GMiDCs were also readily detectable in the bridging channel (BC) and marginal zone (MZ) areas of the spleen (**Supplementary Figure 11**). The superior ability of GMiDCs to capture and present blood-borne antigens might be due to their ubiquitous presence in the spleen.

### Splenic GMiDCs Effectively Polarize Th2 Cells and Induce Allergic Sensitization in Response to Blood-Borne Antigen

**Figure 5:**
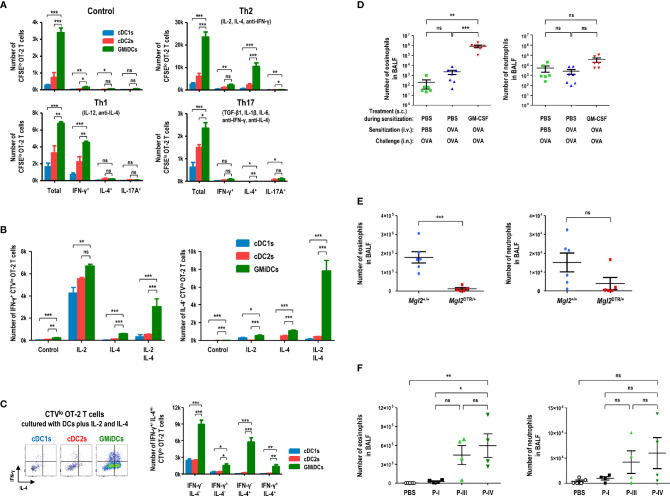
Splenic GMiDCs efficiently polarize Th2 cells and induce allergic sensitization in response to blood-borne antigen. Antigen-presenting splenocytes are isolated from GM-CSF-treated mice at 1 hour after the intravenous injection of OVA and cultured at an APC to T cell ratio of 1:5. **(A)** Proliferation and Th polarization of CFSE-labeled naive CD4^+^ OT-2 T cells stimulated by splenic DC subsets under the respective conditions (Th1: 10 ng/ml IL-12, 5 μg/ml anti-IL-4; Th2: 10 ng/ml IL-2, 10 ng/ml IL-4, 5 μg/ml anti-IFN-γ; Th17: 2 ng/ml TGF-β, 10 ng/ml IL-1β, 20 ng/ml IL-6, 5 μg/ml anti-IFN-γ, 5 μg/ml anti-IL-4). Data are shown in mean ± SEM (n = 3 or 4 replicates) from 3 independent experiments. **(B)** Numbers of proliferated IFN-γ^+^ Th1 (left graph) and IL-4^+^ Th2 (right graph) CellTrace Violet (CTV)^lo^ CD4^+^ OT-2 T cells stimulated by splenic DC subsets under the conditions containing 10 ng/ml IL-2 and/or 10 ng/ml IL-4. Data are shown in mean ± SEM (n = 3 or 4 replicates) from 3 independent experiments. **(C)** Flow cytograms and graphs showing numbers of proliferated IFN-γ^+^ and/or IL-4^+^ CTV^lo^ CD4^+^ OT-2 T cells stimulated by splenic DC subsets under the condition containing IL-2 and IL-4. Data are shown in mean ± SEM (n = 3 or 4 replicates) from 3 independent experiments. **(D)** Graphs showing the numbers of eosinophils (left) and neutrophils (right) in BALF of the mice treated to cause OVA-induced allergic airway inflammation as in **Supplementary Figure 17**. Data are shown in mean ± SEM (n = 6 or 7 mice for each condition). **(E)** Graphs showing the numbers of eosinophils (left) and neutrophils (right) in BALF of the GM-CSF/DT-treated *Mgl2* ^+/+^ and *Mgl2* ^DTR/+^ mice following blood-borne OVA sensitization and OVA-induced allergic airway inflammation as in **Supplementary Figure 18**. Data are shown in mean ± SEM (n = 5 or 6 mice for each genotype). **(F)** Graphs showing the numbers of eosinophils (left) and neutrophils (right) in BALF of the mice treated with GM-CSF, adoptive transfer with blood-borne OVA-sensitized splenic CD11b^+^CD115^hi^ cell subsets, and OVA-induced allergic airway inflammation as in **Supplementary Figure 19**. Data are shown in mean ± SEM (n = 4 or 5 mice for each condition). *p ≤ 0.05; **p ≤ 0.01; ***p ≤ 0.001; ns, not significant; one-way **(D, F)** or two-way **(A-C)** ANOVA test; Student’s *t*-test **(E)**.

Different DC subsets under specific culture conditions can stimulate and polarize naive CD4^+^ T cells into functionally distinct effector/helper or regulatory T cells (22, 52, 53). After delivery of blood-borne OVA, we isolated and compared splenic cDC1s, cDC2s, and GMiDCs for their ability to induce the polarization of naive OT-2 T cells into different Th cells. GMiDCs were significantly superior to cDC1s and cDC2s in stimulating and polarizing OT-2 T cells under all the different conditions we tested (**Figure 5A** and **Supplementary Figure 12**). Noticeably, GMiDCs promoted OT-2 T cells to polarize as well as to proliferate most vigorously in the Th2 condition but also at lower levels under Th1, Th17, and non-polarizing conditions (**Figure 5A** and **Supplementary Figures 12, 13**). Then, we examined the role of individual cytokines (i.e., IL-2 and IL-4) in the Th2 condition. With the addition of IL-2 alone, all of the splenic DC subsets strongly stimulated OT-2 T cells to prominently express IFN-γ; by contrast, when IL-4 was added, GMiDCs became superior to cDC1s and cDC2s in stimulating OT-2 T cells to proliferate and express IL-4 (**Figure 5B** and **Supplementary Figure 14**). Also, in contrast to cDC1s and cDC2s, GMiDCs uniquely induced OT-2 T cells to vigorously proliferate and express both IL-4 and IFN-γ with the addition of IL-2 and IL-4 together (**Figures 5B, C** and **Supplementary Figure 14**). Treatment with cytokine-neutralizing antibodies indicated that this IL-2/IL-4-induced Th polarization by GMiDCs was dependent on IFN-γ and IL-4 but not IL-12 (**Supplementary Figure 15**).

Various phagocytes like macrophages in the spleen are highly proficient at capturing and eliminating blood-borne antigens (49, 50, 54, 55), and therefore low doses of blood-borne antigens are poorly immunogenic. To evaluate the role of splenic GMiDCs *in vivo*, naive OT-2 T cells were adoptively transferred to congenic control and GM-CSF-treated mice, respectively. When OVA was injected intravenously, OT-2 T cells proliferated vigorously in the spleen of GM-CSF-treated mice but proliferated poorly in the spleen of control mice (**Supplementary Figure 16A**). We next utilized the mice expressing diphtheria toxin receptor (DTR) under the control of the *Mgl2* (CD301b) gene (i.e., *Mgl2* ^DTR/+^), and confirmed the specific ablation of CD301b^+^ GMiDCs by diphtheria toxin (DT) treatment (**Supplementary Figure 16B**). Upon the intravenous injection of OVA, the proliferation of adoptively transferred OT-2 T cells was markedly reduced in *Mgl2* ^DTR/+^ mice treated with GM-CSF and DT, as compared to wild-type (WT) *Mgl2* ^+/+^ mice treated with GM-CSF and DT (**Supplementary Figure 16C**), indicating that CD301b^+^ GMiDCs in the spleen are crucial for the robust proliferation of naive CD4^+^ T cells *in vivo* in response to blood-borne antigens. Then, we investigated the role of GMiDCs during the sensitization phase of allergic eosinophilic airway inflammation induced by an inert model allergen OVA. After being treated with control DPBS or GM-CSF, mice were sensitized once with intravenous injection of OVA and challenged intranasally with OVA (**Supplementary Figure 17A**). Massive accumulation of eosinophils in the bronchoalveolar lavage fluids (BALFs) (**Supplementary Figure 17B**) and lung tissues and extensive cellular infiltration into the perivascular and peribronchiolar spaces (data not shown) were observed only in the mice sensitized once with blood-borne OVA under GM-CSF-treated condition; however, almost no eosinophils accumulated in the mice either unsensitized or sensitized once with blood-borne OVA under control DPBS-treated condition (**Figure 5D**), as did the case of unchallenged naive mice (data not shown). The lack of allergic sensitization in control DPBS-treated mice was likely due to the rapid and effective removal of blood-borne antigens by splenic macrophages (49, 50, 54, 55). In the spleen of GM-CSF-treated mice, however, GMiDCs as a new and dominant subset of APCs likely played a critical role in promoting systemic Th2 responses to blood-borne antigens. Next, to verify the role of CD301b^+^ cells including GMiDCs in the sensitization phase of OVA-induced allergic eosinophilic airway inflammation, *Mgl2* ^+/+^ and *Mgl2* ^DTR/+^ mice were treated with GM-CSF/DT and once sensitized with blood-borne OVA, followed by intranasal challenges with OVA (**Supplementary Figure 18A**). The selective ablation of CD301b^+^ cells during the sensitization with blood-borne OVA significantly inhibited OVA-induced allergic eosinophilic airway inflammation (**Figure 5E** and **Supplementary Figure 18B**). We then determined whether the adoptive transfer of splenic GMiDCs could sensitize naive recipient mice to allergic eosinophilic airway inflammation. Three major subsets (P-I, P-III, and P-IV) of CD11b^+^CD115^hi^ cells were isolated from the spleen of GM-CSF-treated, blood-borne OVA-loaded mice. Then, each cell subset was adoptively transferred to naive recipients, followed by intranasal challenges with OVA. However, none of the adoptively transferred cell subsets showed the sensitization effect (data not shown). The lack of sensitization might have been due to steady-state condition of the recipients. Therefore, to more properly evaluate the sensitization potential of CD11b^+^CD115^hi^ splenocytes, each cell subset was adoptively transferred to the naive mice treated with GM-CSF (**Supplementary Figure 19A**). In fact, the mice treated with GM-CSF and adoptively transferred with P-IV subset (i.e., GMiDCs) developed strong allergic eosinophilic airway inflammation, whereas the adoptive transfer of P-I subset (i.e., classical monocytes) only resulted in mild allergic sensitization (**Figure 5F** and **Supplementary Figure 19B**). The adoptive transfer of P-III subset (i.e., named below as pre-GMiDCs) also led to relatively strong allergic sensitization, implying that P-III subset and GMiDCs might be related functionally and/or developmentally.

### GMiDCs Are Derived From Ly6C^+^ Classical Monocytes

**Figure 6:**
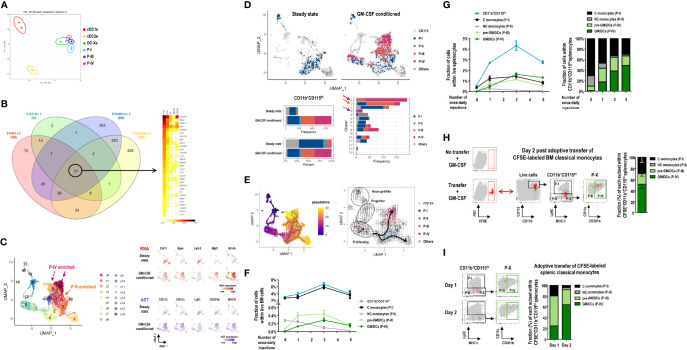
Ly6C^+^ classical monocytes differentiate into GMiDCs in the spleen. **(A)** PCA of transcriptomes of splenic cDC1s, cDC2s, DC-Xs, classical monocytes (C monocytes, P-I), P-III, and GMiDCs (P-IV) in GM-CSF-treated mice (n = 2 mice for each cell population). **(B)** Genes in each circle of Venn diagram (left) are expressed at least twice higher in the P-IV than the respective other populations. Heatmap (right) showing the 31 P-IV-enriched genes in descending order of expression. **(C)** Uniform manifold approximation and projection (UMAP) of 6,075 cells in the CD11b^+^ splenocytes excluding T cells, B cells, granulocytes, neutrophils, and cDCs. Expression levels of 5 marker genes (*Csf1r*/CD115*, Itgax*/CD11c*, Ly6c2*/Ly6C*, Mgl2*/CD301b, and *H2-A2*/MHCII) are detected by CITE-seq analyses. Each cell is colored by cluster (left), RNA expression level (right upper), or antibody-derived tag (ADT) level (right lower). **(D)** Constitution of P-I, P-II, P-III, P-IV, and other CD115^hi^ cells in the spleen of steady-state and GM-CSF-treated mice. UMAP (upper) and bar graphs (lower) are color-coded according to ADT-based CD115^hi^ cell subsets. **(E)** Pseudotemporal analyses showing pseudotime (left) estimated by Monocle3 with the cluster 9 (progenitor-like cells) assigned as root and RNA velocity (right) inferred by the velocyto pipeline with thick arrows representing the directional flow of thin velocity arrows. **(F, G)** Changes in the fractions of CD11b^+^CD115^hi^ cells and their subsets, i.e., C monocytes (P-I), non-classical (NC) monocytes (P-II), pre-GMiDCs (P-III), and GMiDCs (P-IV) in the BM **(F)** and the spleen **(G)** analyzed at 24 hours after different numbers of once-daily injections of GM-CSF. Data are shown in mean ± SEM (n = 4 mice for each condition). **(H, I)** Flow cytograms and graphs showing CFSE^+^ cells in the splenic CD11b^+^CD115^hi^ cell subsets of GM-CSF-treated mice after the adoptive transfer of CFSE-labeled classical monocytes isolated from the BM **(H)** and the spleen **(I)**. CFSE^+^ cells (red dots) in the spleen are backgated in a series of dot plots of total live-gated cells (gray dots) to identify CD11b^+^CD115^hi^ cell subsets (left panels). Graphs (right) showing mean ± SEM (**H,** n = 3 mice) or mean (**I,** n = 2 mice for each condition) of the fraction for each cell subset.

Three major subsets (P-I, P-III, and P-IV) of the splenic CD11b^+^CD115^hi^ cells in GM-CSF-treated mice (**Figure 3E**) were subject to bulk RNA-seq analysis. PCA of transcriptomic data indicated that the gene expression profiles of CD11b^+^CD115^hi^ cell subsets clustered closer than those of cDC1s and cDC2s (**Figure 6A**). Particularly, the clusters of GMiDCs (P-IV) and P-III cells were located very near each other. As compared to all the other splenic subsets of DCs and CD11b^+^CD115^hi^ cells, we identified the upregulated expression of 31 genes, such as *Cd209a*, *Mgl2*, *Mmp12*, *Mt1*, *Cd200r1*, etc., in GMiDCs (**Figure 6B** and **Supplementary Figure 20**). In addition, the diversities of CD11b^+^ splenocytes from the mice treated with or without GM-CSF were probed by single-cell analyses using cellular indexing of transcriptomes and epitopes by sequencing (CITE-seq) method. An unsupervised uniform manifold approximation and projection (UMAP) was performed and cell clusters were annotated based on the expression of selective representative genes. Then, a further UMAP analysis was carried out after excluding clusters for the lineage cells containing T cells, B cells, granulocytes, neutrophils, and cDCs (**Figure 6C** and **Supplementary Figure 21**), and the clusters pertaining to CD115^hi^ cell subsets were identified on the basis of CITE-seq analyses (**Figure 6D** and **Supplementary Figure 22**). Then, 32 differentially expressed genes (DEGs) in the clusters containing GMiDCs were determined (**Supplementary Figure 23**), and 10 of those 32 DEGs overlapped with the 31 DEGs identified from the bulk RNA-seq (**Figure 6B**). The analyses of pseudotime cell trajectories and RNA velocity (**Figure 6E**) revealed that P-III and GMiDCs (P-IV) subsets arose from classical monocytes (P-I). The transitions from P-I to P-III and P-III to P-IV were observed in 2 separate groups of clusters, one including clusters 7 and 8 and the other including clusters 0, 1, 3, 5, and 10 (**Figures 6C-E**). Especially, the cells in clusters 7 and 8 were enriched for proliferation and differentiation signatures, and their RNA velocities were significantly higher than those in the other group (**Figure 6E** and **Supplementary Figures 24, 25**). The normalized expression of signature genes for Th2 polarization and antigen uptake was elevated in GMiDCs (P-IV) subset (**Supplementary Figure 26**). Similar to the flow cytometry data (**Supplementary Figure 8**), the expression level of CD209a RNA could subdivide the cells in GMiDCs (P-IV) subset (**Supplementary Figures 27A, B**) but was not related to the expression levels of macrophage marker genes (**Supplementary Figure 27C**).

To further corroborate the findings from CITE-seq analyses, we examined the sequential development of CD11b^+^CD115^hi^ cell subsets following GM-CSF injections. In the bone marrow (BM), the vast majority of CD11b^+^CD115^hi^ cells were classical monocytes (P-I) regardless of GM-CSF injections. With once-daily injections of GM-CSF, P-III cells increased sharply followed by GMiDCs (P-IV), but non-classical monocytes (P-II) gradually disappeared in the BM (**Figure 6F**). In the spleen, once-daily injections of GM-CSF also increased the numbers of P-III cells, GMiDCs, and classical monocytes, but not non-classical monocytes, and GMiDCs became the most numerous of the CD11b^+^CD115^hi^ cells after 3 once-daily injections of GM-CSF (**Figure 6G** and **Supplementary Figure 28A**). When the once-daily injections of GM-CSF were continued for 7 days, the increased numbers of classical monocytes were not altered but those of P-III and GMiDCs were decreased significantly in the spleen (**Supplementary Figure 28B**). Meanwhile, unlike CD11b^+^CD115^hi^ cell subsets, the numbers of splenic F4/80^+^ macrophages were maintained before and after the once-daily injections of GM-CSF (**Supplementary Figure 28B**). Next, to verify the development potential to differentiate into GMiDCs *in vivo*, Ly6C^+^ classical monocytes were isolated from the BM of untreated control mice, labeled with carboxyfluorescein succinimidyl ester (CFSE), and adoptively transferred to the recipient mice treated with GM-CSF. In the spleen, at 2 days after adoptive transfer, a half of the CFSE-labeled CD11b^+^CD115^hi^ cells were GMiDCs, and much less were P-III cells and monocytes (**Figure 6H**). Then, we labeled and adoptively transferred classical monocytes (P-I), isolated from the spleen of GM-CSF-treated mice, to the GM-CSF-treated recipients. In the spleen, at 1 day after adoptive transfer, more than a half of the CFSE-labeled CD11b^+^CD115^hi^ cells were P-III cells; at 2 days after adoptive transfer, more than a half of the CFSE-labeled CD11b^+^CD115^hi^ cells were GMiDCs (**Figure 6I**). Also, we isolated P-III cells and GMiDCs (P-IV) respectively from the spleen of GM-CSF-treated mice, labeled with CFSE, and adoptively transferred to the GM-CSF-treated recipients. In the spleen, at 2 days after adoptive transfer of GMiDCs, no CFSE-labeled CD11b^+^CD115^hi^ cells were detected; however, at 2 days after adoptive transfer of P-III cells, most of the CFSE-labeled CD11b^+^CD115^hi^ cells in the spleen were GMiDCs (**Supplementary Figure 29**). Therefore, classical monocytes are precursors for P-III cells and GMiDCs, and P-III cells are pre-GMiDCs.

### Differentiation of GMiDCs Requires the *Cis* Expression of GM-CSF Receptor

**Figure 7:**
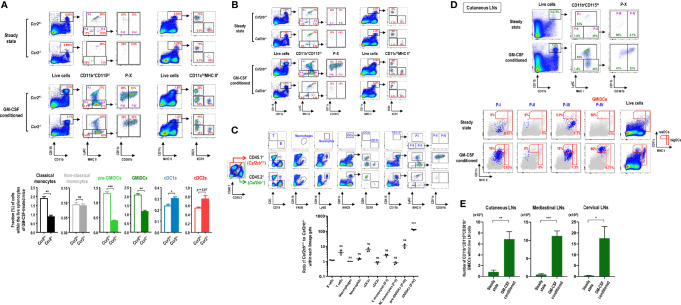
Differentiation of GMiDCs from classical monocytes necessitates the *cis* expression of GM-CSF receptor. **(A)** Gating strategy (upper panels) and analysis (lower graphs) showing differences in the fractions of DC and CD11b^+^CD115^hi^ cell subsets in the spleen between *Ccr2* ^+/-^ and *Ccr2* ^-/-^ mice treated with (lower graphs) or without GM-CSF. Data are shown in mean ± SEM (n = 3 for each genotype and condition). **(B)** Gating strategy showing differences in the fractions of DC and CD11b^+^CD115^hi^ cell subsets in the spleen between *Csf2rb* ^+/-^ and *Csf2rb* ^-/-^ mice treated with or without GM-CSF. Representative flow cytograms (n = 3 for each genotype and condition). **(C)** Gating strategy (upper panels) and the ratio of CD45.1 cells to CD45.2 cells (lower graph) of each Lin^+^ population in GM-CSF-treated mixed BM chimeric mice at 6 weeks after adoptive transfer of *Csf2rb* ^+/+^ (CD45.1) and *Csf2rb* ^-/-^ (CD45.2) BM at a 1:1 ratio into lethally irradiated WT (CD45.1) recipients. Data are shown in mean ± SEM (n = 3 mice) and compared to the mean of the B cells. **(D)** Gating strategy identifying CD11b^+^CD115^hi^ cell subsets in cutaneous LNs of control and GM-CSF-treated mice (upper). Each population (blue dots) of CD11b^+^CD115^hi^ cell subsets in the LNs is backgated in an MHC II versus CD11c dot plot of total live-gated cells (gray dots), indicating the fraction of each subset within the respective DC gates of resident (resDC) and migratory (migDC) phenotypes (lower). Representative flow cytograms (n = 4 mice for each condition). **(E)** Graphs showing the number of CD11b^+^CD115^hi^CD301b^+^ GMiDCs cells in the respective LNs of control and GM-CSF-treated mice according to the gating strategies as in **(D)** and **Supplementary Figure 35**. Data are shown in mean ± SEM (n = 4 mice for each condition). *p ≤ 0.05; **p ≤ 0.01; ***p ≤ 0.001; ns, not significant; Student’s *t*-test **(A, E)**; one-way ANOVA test **(C)**.

Since BM classical monocytes were found to differentiate into splenic GMiDCs, we examined the effect of GM-CSF treatment in mice deficient of CCR2, a key chemokine receptor for the egress of BM classical monocytes at steady state (56). In CCR2 knockout (KO) mice treated with GM-CSF (**Figure 7A**), classical monocytes as well as pre-GMiDCs and GMiDCs were significantly reduced in the spleen, indicating that splenic GMiDCs substantially derived from BM classical monocytes. Meanwhile, the deficiency of CCR7, an essential chemokine receptor for DC migration from tissues to lymphoid organs (57) including the spleen (58), did not alter the fraction of GMiDCs in the spleen of GM-CSF-treated mice (**Supplementary Figure 30**), suggesting that the development of GMiDCs was not dependent on CCR7. Given that the transcription factor *Irf4* is upregulated in both cDC2s and GMiDCs under GM-CSF condition (**Supplementary Figure 20**), we analyzed the *Irf4* ^fl/fl^ mice crossed with the *Itgax-*cre mice that express Cre recombinase in DCs (59). Both cDC2s and GMiDCs were most conspicuously reduced in *Itgax*-cre *Irf4* ^fl/fl^ mice, which confirmed that *Irf4* was involved in the development of both cDC2s and Mo-DCs (59–64) including GMiDCs (**Supplementary Figure 31**). However, the deletion of *Batf3*, a transcription factor essential for cDC1 differentiation, did not affect the development of GMiDCs or cDC2s (**Supplementary Figure 32**).

GM-CSF receptor (*Csf2ra* and *Csf2rb*) is expressed in most of myeloid cells (9). As expected, GM-CSF treatment produced no effect in *Csf2rb* KO mice (**Figure 7B**). We next generated mixed BM chimeras by adoptively transferring an equal mixture of BM cells from WT *Csf2rb* ^+/+^ (CD45.1) and KO *Csf2rb* ^-/-^ (CD45.2) mice into lethally irradiated WT *Csf2rb* ^+/+^ (CD45.1) recipients; the mixed BM chimeric mice were treated with GM-CSF after 6 weeks of adoptive cell transfer. The results demonstrated that GMiDCs were almost exclusively derived from the BM of WT *Csf2rb* ^+/+^ mice (**Figure 7C**), which clearly indicated that the differentiation of GMiDCs required the *cis* expression of GM-CSF receptor in their precursors. Meanwhile, ca. 90% of pre-GMiDCs were derived from the BM of WT mice, and ca. 70% of classical monocytes were derived from the BM of WT mice. Notably, cDC1s were predominantly derived from the BM of WT mice during GM-CSF treatment but not in the steady state (**Figures 7B, C**), verifying that direct GM-CSF receptor signaling was involved in regulating the size of cDC1 population (65). The deletion of *Csf2rb* in the BM did not influence the GM-CSF-induced development of other myeloid cells such as non-classical monocytes, macrophages, cDC2s, and neutrophils (**Figure 7C**). The biased over-representation of WT (CD45.1) T cells (**Figure 7C**) was likely due to the radiation-resistant T cells originating from the recipient host mice (66, 67). Then, we generated mixed BM chimeric mice of WT *Csf2rb* ^+/+^ (CD45.2) and KO *Csf2rb* ^-/-^ (CD45.1) in lethally irradiated KO *Csf2rb* ^-/-^ (CD45.1) recipients, where GMiDCs were also exclusively derived from the BM of WT *Csf2rb* ^+/+^ mice (**Supplementary Figure 33**).

### GMiDCs Mobilize to Peripheral LNs and Tissues

Very similarly as seen in the spleen (**Supplementary Figure 16**), in response to blood-borne OVA, the robust proliferation of adoptively transferred naive OT-2 T cells was observed in the cutaneous LNs of GM-CSF-treated mice but the poor proliferation in those of control mice (data not shown), suggesting that GMiDCs might exist in the LNs of GM-CSF-treated mice. Also, our previous study demonstrated that the treatment with B16 melanomas expressing GM-CSF increased the number of CD301b^+^ resident DCs (resDCs) in cutaneous LNs (29). Indeed, following treatment with GM-CSF, CD11b^+^CD115^hi^Ly6C^-^MHC II^+^CD301b^+^ GMiDCs were generated in cutaneous LNs and localized predominantly in resDCs but rarely in migratory DCs (migDCs), whereas GMiDCs were hardly detectable in steady-state LNs (**Figure 7D**). As we reported previously (29), most of the CD301b^+^ DCs in the steady-state LNs were migratory cDC2s with low expression of CD115 (data not shown). Like other resDCs of the LNs (68), the generation of GMiDCs in the LNs was independent of CCR7 expression (**Supplementary Figure 34**). GMiDCs were also generated within the resDCs of the LNs at various locations we examined, such as mediastinal and cervical LNs (**Figure 7E** and **Supplementary Figure 35**). We next examined the generation of GMiDCs in peripheral tissues. As compared to *Csf2rb* ^-/-^ mice, CD11b^+^CD115^hi^CD301b^+^ GMiDCs were not detected in the dermis (**Supplementary Figure 36A**) and generated moderately in the intestines (**Supplementary Figures 36B, C**) of the mice treated with GM-CSF. Unlike other peripheral tissues examined, CD11b^+^CD115^hi^ cells increased markedly by treatment with GM-CSF in the lung (**Supplementary Figure 36D**) where the increases of pre-GMiDCs (P-III) and GMiDCs (P-IV) were most prominent.

It was notable that GMiDCs existed as a significant fraction of CD11b^+^CD115^hi^ cells in the lung of the steady-state mice as compared to the lung of mice deficient of GM-CSF signaling (**Supplementary Figure 36D**). It is likely due to the relatively high expression of endogenous GM-CSF in the steady-state lung (9). Therefore, we further investigated whether GMiDCs were generated under the inflammatory condition where the production of GM-CSF was augmented (69). We treated mice with daily intranasal administration of *Alternaria* extract. After more than a week of daily treatment with allergen *Alternaria*, the accumulation of inflammatory eosinophils in both BALFs and lung tissues became prominent (**Supplementary Figure 37A**); in the lung-draining mediastinal LNs as well as in the spleen, increased levels of GMiDCs were evident (**Supplementary Figures 37B, C**). Next, to verify whether the generation of GMiDCs by treatment with *Alternaria* was dependent on GM-CSF signaling, we tested the *Csf2* KO and *Csf2rb* KO mice. As compared to *Csf2* ^+/-^ and *Csf2rb* ^+/-^ mice, the accumulation of inflammatory eosinophils in the BALF was markedly reduced in *Csf2* ^-/-^ (**Supplementary Figure 37D**) and *Csf2rb* ^-/-^ (data not shown) mice; in the lung as well as in the spleen, only the number of GMiDCs was significantly reduced among the CD11b^+^CD115^hi^ cell subsets in the mice deficient of GM-CSF signaling (**Supplementary Figures 37E-G**).

## Discussion

We discovered a novel subset of splenic DCs (i.e., GMiDCs) generated under GM-CSF-enriched conditions. Up to our knowledge, GMiDCs are the most abundant, authentic Mo-DCs in the spleen under any circumstances. Likely due to the unique role of spleen as a blood filter (49, 50), GMiDCs in the spleen might function as highly effective antigen-presenting cells that orchestrate systemic immunity to blood-borne antigen. Interestingly, while GMiDCs could boost Th1 responses better than cDC subsets, GMiDCs exhibited an exceptional ability to polarize and expand Th2 cells in response to blood-borne antigen. Therefore, it was important to examine whether CD301b^+^ GMiDCs in the spleen could induce or sensitize Th2 responses and thus to promote allergic inflammation *in vivo* in response to blood-borne antigen. In the current view of allergic sensitization and development (21, 27), DCs in or near barrier tissues are skewed and influenced by pro-allergic cytokines derived from allergen-stimulated barrier epithelial cells; the activation and CCR7-dependent migration of pro-allergic DCs to the draining LNs are essential for mounting allergen-specific Th2 polarization and subsequent allergic responses; CD301b^+^ migDCs in the skin-draining LNs were demonstrated to stimulate CD4^+^ T cells to polarize towards Th2 cells *in vivo* following the subcutaneous treatment with Th2-type adjuvant (28). In the present study, we demonstrated that splenic GMiDCs were able to sensitize mice to a blood-borne OVA, an inert allergen, in a model of allergic eosinophilic airway inflammation. When we examined the expression of CD301b from cDCs in the steady-state spleen, a small subset of cDC2s expressed CD301b, as previously reported (23). Since blood-borne antigen did not sensitize the control DPBS-treated mice in allergic eosinophilic airway inflammation model, the small subset of CD301b^+^ cDC2s in the steady-state spleen seemed unable to induce allergic sensitization or Th2 immunity. Besides the spleen, GMiDCs were also generated in the CCR7-independent resDCs of the LNs and in some peripheral tissues with GM-CSF treatment. Meanwhile, we also found that small but significant numbers of GMiDCs were generated in the spleen and other tissues during chronic allergic inflammation. Collectively, our present findings suggest that some, if not much, of the sensitization and progression of allergies may occur systemically through the presentation of allergens by GMiDCs in the spleen. Further works are required to investigate the specific roles of GMiDCs in various tissues and LNs considering that the resDCs in the LNs were recently shown to drive Th2 polarization (70).

Many anti-cancer immunotherapies based on GM-CSF were proposed with promising results in their early stages of development and clinical trials, but most of them have eventually failed due to poor clinical benefits (71, 72). Accordingly, GM-CSF is now considered to have both immunostimulatory and immunosuppressive effects. The role of GM-CSF in the development of DCs is viewed as immunostimulatory, whereas its role in the generation of myeloid-derived suppressor cells as immunosuppressive (71). However, our current work shows that GM-CSF preferentially induces Th2-polarizing GMiDCs in the spleen, which likely limits anti-cancer immunity (73), although GMiDCs can also boost Th1 responses better than cDC subsets. We also find that the intravenous delivery of OVA in GM-CSF-treated mice generates only mild or insignificant enhancement of protective immunity against OVA-expressing tumor cells (our unpublished data). Therefore, DCs derived from GM-CSF-based immunotherapies are expected to play both immunostimulatory and immunosuppressive roles.

According to our current study on single-cell analyses using CITE-seq, pre-GMiDCs (P-III) and GMiDCs (P-IV) subsets are differentiated from classical monocytes (P-I) subset. In the spleen, these transitions from classical monocytes to pre-GMiDCs then to GMiDCs appear to occur in 2 separate modes, one enriched with high expression of genes for proliferation and differentiation signatures but not in the other. Within the GMiDCs (P-IV) subset, there exist CD209a positive and negative subpopulations but the expression levels of other signature genes do not differ significantly between those. Meanwhile, in the experiments with mixed BM chimeras of *Csf2rb* ^+/+^ and *Csf2rb* ^-/-^ mice, the differentiation of GMiDCs and pre-GMiDCs requires GM-CSF signaling through the GM-CSF receptor expressed in *cis* in their precursor cells, but the differentiation of Ly6C^+^ and Ly6C^-^ monocytes does not. However, unlike GMiDCs, other Mo-DCs were generated independently of GM-CSF during inflammation (9). Interestingly, a recent study with a mouse model of experimental autoimmune encephalomyelitis (EAE) revealed that the conditional deletion of *Csf2rb* in CCR2^+^Ly6C^+^ classical monocytes inhibited the neuroinflammation, but the conditional deletion of *Csf2rb* in other myeloid cells including cDCs and Mo-DCs did not (74). Since Mo-DCs in the inflamed brain developed normally from classical monocytes independently of GM-CSF, the role of GM-CSF signaling was suggested to license an inflammatory program to classical monocytes before their becoming pathogenic Mo-DCs (74, 75). It is not known yet whether Mo-DCs in the inflamed brain of EAE mice contain GMiDCs. However, our findings imply that the conditional deletion of *Csf2rb* in classical monocytes likely prevents the development of GM-CSF-dependent Mo-DCs in EAE mice, but not the development of GM-CSF-independent Mo-DCs. It also raises a possibility that GMiDCs or GM-CSF-dependent Mo-DCs might involve inflammatory immune responses.

When Mo-DCs were generated *in vitro*, either from the BM cells cultured with GM-CSF (29, 76) or from the splenocytes cultured with GM-CSF (8), they all exhibited CD115^lo^CD301b^+^ phenotype. Hence, there likely exist unknown factor(s) and pathway(s) for the differentiation of CD115^hi^CD301b^+^ GMiDCs *in vivo*. Along with discovering a splenic subset of monocyte-derived GMiDCs that efficiently capture and present blood-borne antigens, our findings provide clues to understand the complex roles of GM-CSF in inflammation and immunity.

## Data Availability Statement 

The datasets presented in this study can be found in online repositories. The names of the repository/repositories and accession number(s) can be found below: https://www.ncbi.nlm.nih.gov/geo/query/acc.cgi?acc=GSE157945, https://www.ncbi.nlm.nih.gov/geo/query/acc.cgi?acc=GSE158200.

## Ethics Statement 

The animal study was reviewed and approved by Institutional Animal Care and Use Committees of the Yonsei University College of Medicine.

## Author Contributions

Conceptualization: CP. Methodology: SR, HS, WC, HN, HI, JP, SP, and CP. Formal Analysis: SR, HE, JP, and H-OL. Investigation: SR, HS, WC, HN, HI, T-GK, JP, SP, SH, and MS. Resources: TK, E-DK, KS, and M-GL. Data Curation: SR and MS. Writing – Original Draft: SR, HS, HE, WC, HN, HI, JP, SP, and CP. Writing – Review & Editing: SR, T-GK, H-OL, and CP. Visualization: SR, HS, HE, HN, HI, JP, and SP. Supervision: H-OL and CP. Project Administration: SR and HN. Funding Acquisition: HN, KS, H-OL, MC, and CP. All authors contributed to the article and approved the submitted version.

## Funding

This work was supported by grants from the National Research Foundation of Korea to CP (2014R1A4A1008625, 2017R1D1A1B03028385, 2017M3A9C8064887, 2019M3A9 B6064691, 2019R1F1A1041700), HN (2017R1A6A3A11028388, 2021R1I1A1A01043872), H-OL (2019M3A9B6064691), MC (2019R1F1A1053841), and KS (2013M3A9D5072551); the Brain Korea 21 PLUS/FOUR Project for Medical Science, Yonsei University. The authors declare that this study received funding from Genuv Inc. The funder was not involved in the study design, collection, analysis, interpretation of data, the writing of this article or the decision to submit it for publication.

## Conflict of Interest

Author CP is employed by Genuv Inc.

The remaining authors declare that the research was conducted in the absence of any commercial or financial relationships that could be construed as a potential conflict of interest.

## Publisher’s Note

All claims expressed in this article are solely those of the authors and do not necessarily represent those of their affiliated organizations, or those of the publisher, the editors and the reviewers. Any product that may be evaluated in this article, or claim that may be made by its manufacturer, is not guaranteed or endorsed by the publisher.

## Abbreviations

APCs, antigen presenting cells; BALF, bronchoalveolar lavage fluids; BC, bridging channel; BM, bone marrow; BWT, Burrows–Wheeler transform; C, classical; cDC, classical dendritic cells; CFSE, carboxyfluorescein succinimidyl ester; CITE-seq, cellular indexing of transcriptomes and epitopes by sequencing; CTV, CellTrace Violet; DCs, dendritic cells; DEGs, differentially expressed genes; DPBS, Dulbecco’s phosphate-buffered saline; DT, diphtheria toxin; DTR, DT receptor; EAE, experimental autoimmune encephalomyelitis; FITC, fluorescein isothiocyanate; FLT3L, FMS-like tyrosine kinase 3 ligand; FMO, fluorescence minus one; GEO, Gene Expression Omnibus; GFM, graph FM index; GFP, green fluorescent protein; GM-CSF, granulocyte macrophage-colony stimulating factor; GMiDCs, GM-CSF-induced DCs; HCA, hierarchical cluster analysis; HRP, horseradish peroxidase; KO, knockout; Lin, lineage; LNs, lymph nodes; LPS, lipopolysaccharide; M-CSF, macrophage-colony stimulating factor; MFI, mean fluorescence intensity; migDCs, migratory DCs; Mo-DCs, monocyte-derived DCs; MZ, marginal zone; NC, non-classical; OCT, optimum cutting temperature; OVA, ovalbumin; PCA, principal component analysis; RBC, red blood cell; resDCs, resident DCs; RP, red pulp; Th cell, T helper cell; TLRs, toll-like receptors; TSA, tyramide signal amplification; UMAP, uniform manifold approximation and projection; WP, white pulp; WT, wild-type.
