# Supplementary material for: Granulocyte Macrophage-Colony Stimulating Factor Produces a Splenic Subset of Monocyte-Derived Dendritic Cells That Efficiently Polarize T Helper Type 2 Cells in Response to Blood-Borne Antigen
Source: Front Immunol. 2022 Jan 3;12:767037. doi: 10.3389/fimmu.2021.767037 (PMC8778578; doi:10.3389/fimmu.2021.767037)
Supplement: Supplementary file 1 [file Presentation_1.zip › Ryu et al - FI - Supplementary Figures - Production.PPTX]

## Slide 1
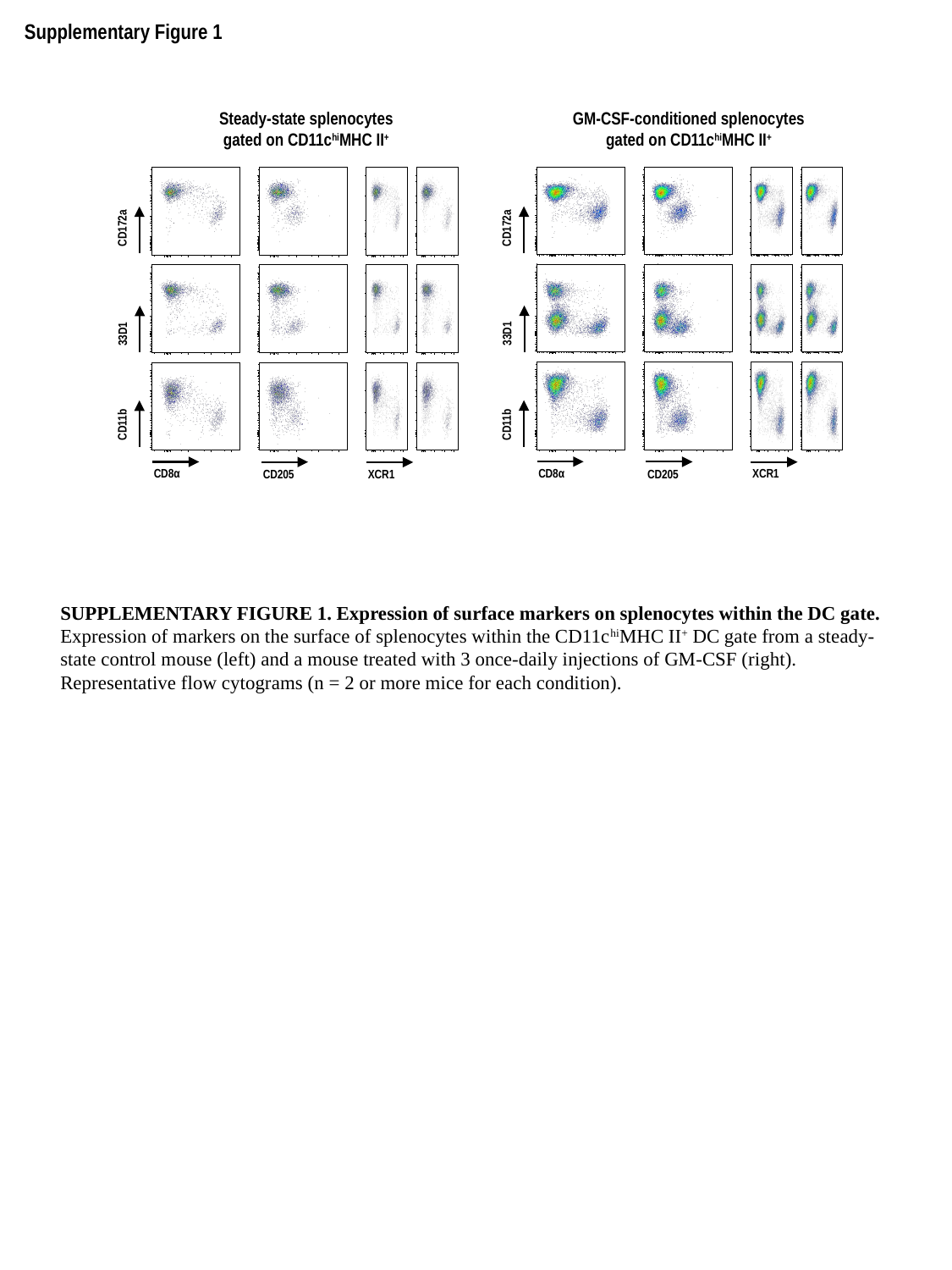

Supplementary Figure 1
Steady-state splenocytes
gated on CD11chiMHC II+
CD172a
33D1
CD11b
CD8α
XCR1
CD205
GM-CSF-conditioned splenocytes
gated on CD11chiMHC II+
CD172a
33D1
CD11b
CD8α
XCR1
CD205
SUPPLEMENTARY FIGURE 1. Expression of surface markers on splenocytes within the DC gate.
Expression of markers on the surface of splenocytes within the CD11chiMHC II+ DC gate from a steady-state control mouse (left) and a mouse treated with 3 once-daily injections of GM-CSF (right). Representative flow cytograms (n = 2 or more mice for each condition).

## Slide 2
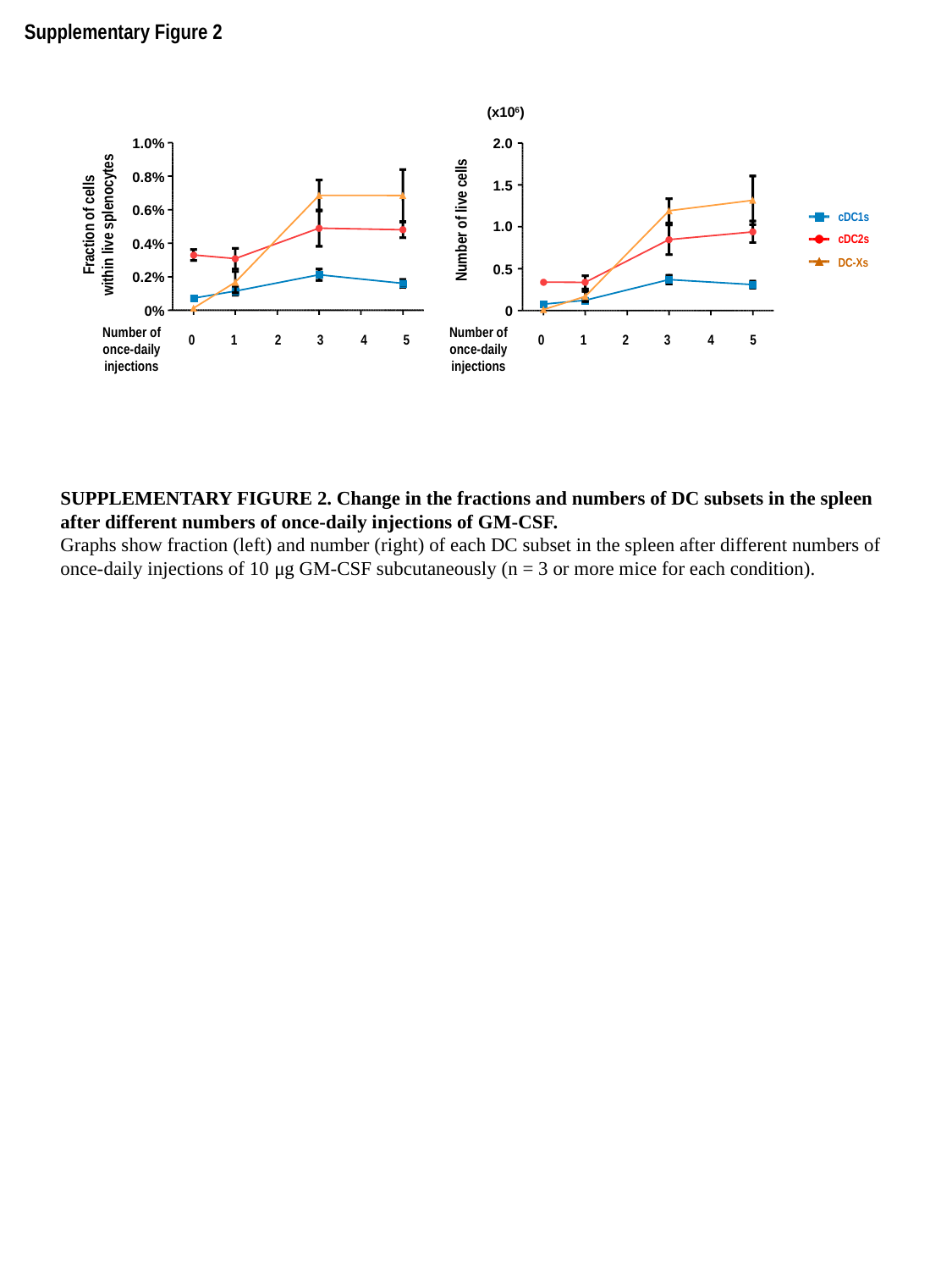

Supplementary Figure 2
(x106)
2.0
1.5
1.0
0.5
0
Number of live cells
Number of
once-daily
injections
0
1
2
3
4
5
1.0%
0.8%
0.6%
0.4%
0.2%
0%
Fraction of cells
within live splenocytes
Number of
once-daily
injections
0
1
2
3
4
5
cDC1s
cDC2s
DC-Xs
SUPPLEMENTARY FIGURE 2. Change in the fractions and numbers of DC subsets in the spleen after different numbers of once-daily injections of GM-CSF.
Graphs show fraction (left) and number (right) of each DC subset in the spleen after different numbers of once-daily injections of 10 μg GM-CSF subcutaneously (n = 3 or more mice for each condition).

## Slide 3
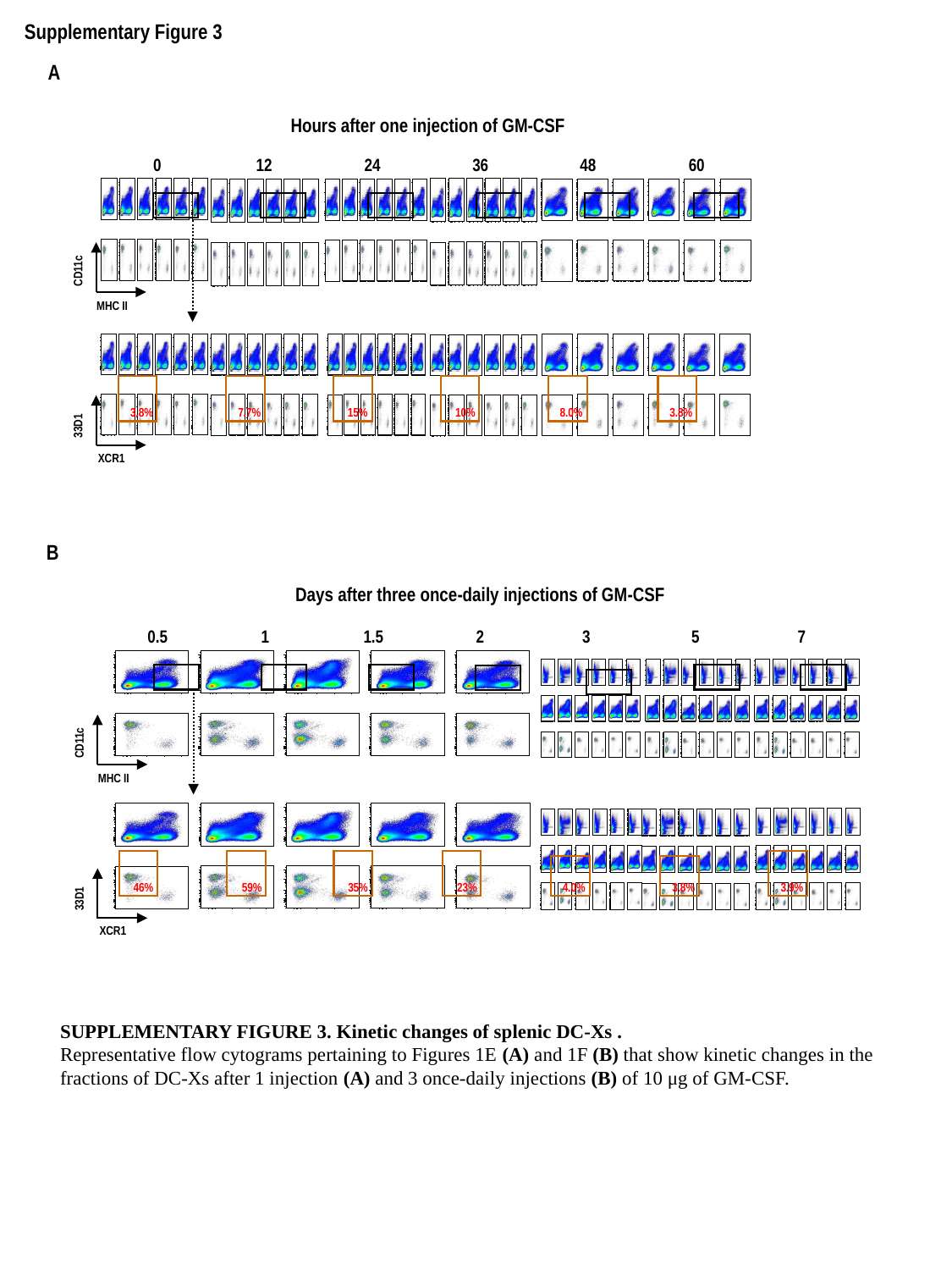

Supplementary Figure 3
A
Hours after one injection of GM-CSF
0
12
24
36
48
60
CD11c
MHC II
33D1
XCR1
3.8%
7.7%
15%
10%
8.0%
3.8%
B
Days after three once-daily injections of GM-CSF
0.5
1
1.5
2
3
5
7
CD11c
MHC II
33D1
XCR1
46%
59%
35%
23%
4.0%
3.8%
3.9%
SUPPLEMENTARY FIGURE 3. Kinetic changes of splenic DC-Xs .
Representative flow cytograms pertaining to Figures 1E (A) and 1F (B) that show kinetic changes in the fractions of DC-Xs after 1 injection (A) and 3 once-daily injections (B) of 10 μg of GM-CSF.

## Slide 4
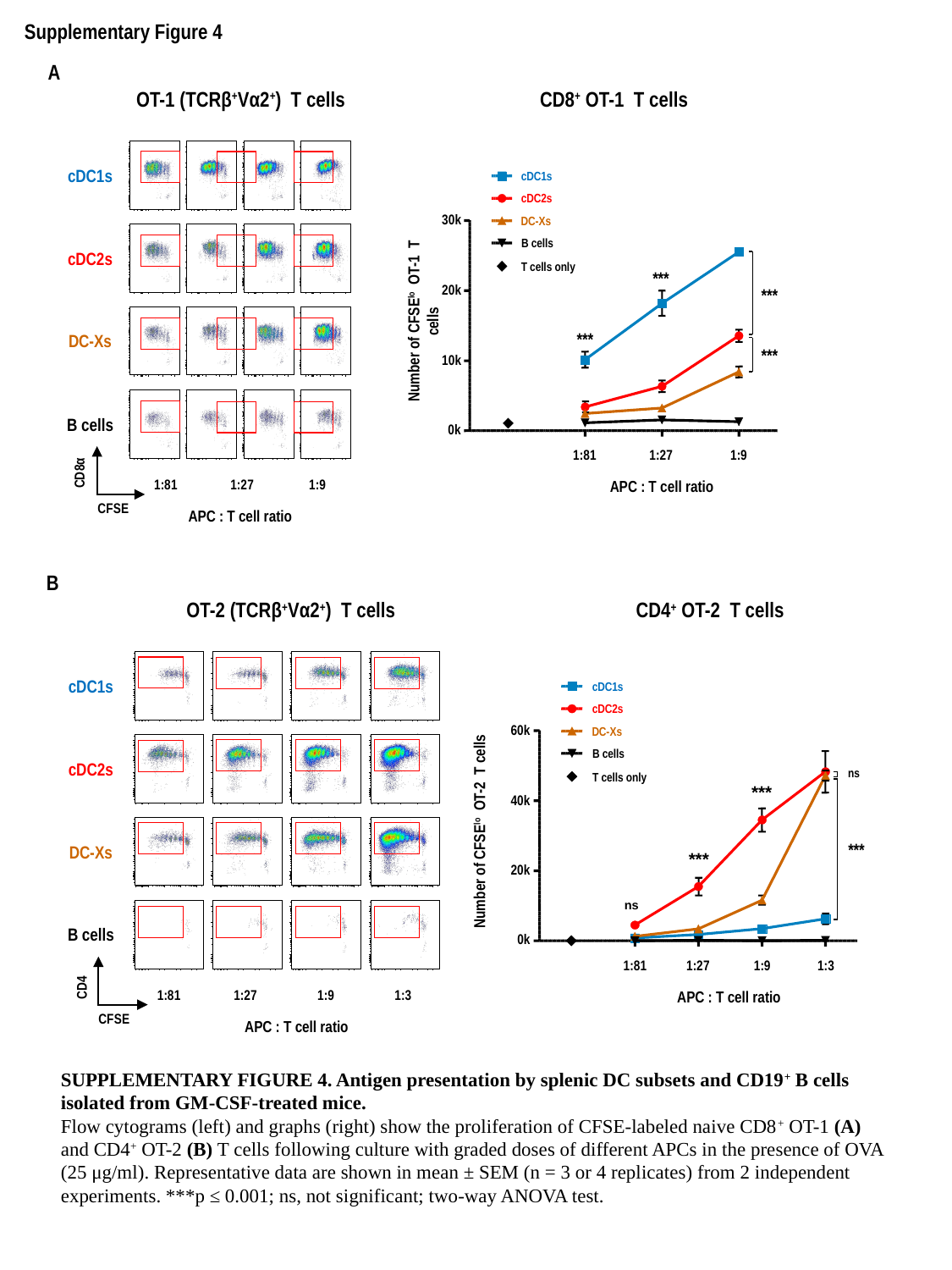

Supplementary Figure 4
A
 OT-1 (TCRβ+Vα2+) T cells
cDC1s
cDC2s
DC-Xs
B cells
CD8α
CFSE
1:81
1:27
1:9
APC : T cell ratio
CD8+ OT-1 T cells
cDC1s
cDC2s
DC-Xs
B cells
T cells only
30k
***
***
20k
***
***
10k
0k
Number of CFSElo OT-1 T cells
1:81
1:27
1:9
APC : T cell ratio
B
 OT-2 (TCRβ+Vα2+) T cells
cDC1s
cDC2s
DC-Xs
B cells
CD4
CFSE
1:81
1:27
1:9
1:3
APC : T cell ratio
CD4+ OT-2 T cells
cDC1s
cDC2s
DC-Xs
B cells
T cells only
60k
ns
***
40k
***
***
20k
ns
0k
Number of CFSElo OT-2 T cells
1:81
1:27
1:9
1:3
APC : T cell ratio
SUPPLEMENTARY FIGURE 4. Antigen presentation by splenic DC subsets and CD19+ B cells isolated from GM-CSF-treated mice.
Flow cytograms (left) and graphs (right) show the proliferation of CFSE-labeled naive CD8+ OT-1 (A) and CD4+ OT-2 (B) T cells following culture with graded doses of different APCs in the presence of OVA (25 μg/ml). Representative data are shown in mean ± SEM (n = 3 or 4 replicates) from 2 independent experiments. ***p ≤ 0.001; ns, not significant; two-way ANOVA test.

## Slide 5
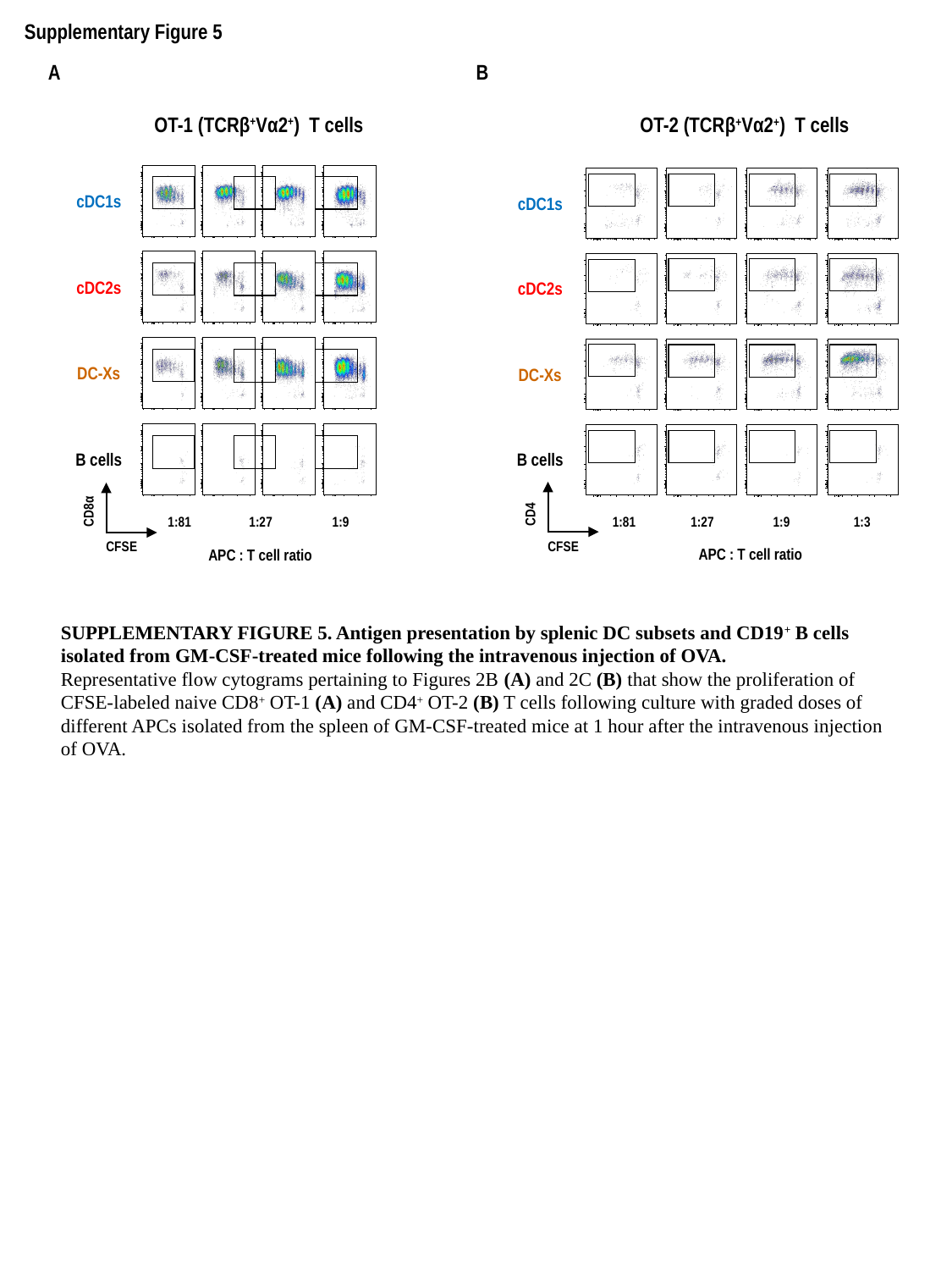

Supplementary Figure 5
A
B
 OT-1 (TCRβ+Vα2+) T cells
 OT-2 (TCRβ+Vα2+) T cells
cDC1s
cDC1s
cDC2s
cDC2s
DC-Xs
DC-Xs
B cells
B cells
CD4
CFSE
CD8α
CFSE
1:81
1:27
1:9
1:3
APC : T cell ratio
1:81
1:27
1:9
APC : T cell ratio
SUPPLEMENTARY FIGURE 5. Antigen presentation by splenic DC subsets and CD19+ B cells isolated from GM-CSF-treated mice following the intravenous injection of OVA.
Representative flow cytograms pertaining to Figures 2B (A) and 2C (B) that show the proliferation of CFSE-labeled naive CD8+ OT-1 (A) and CD4+ OT-2 (B) T cells following culture with graded doses of different APCs isolated from the spleen of GM-CSF-treated mice at 1 hour after the intravenous injection of OVA.

## Slide 6
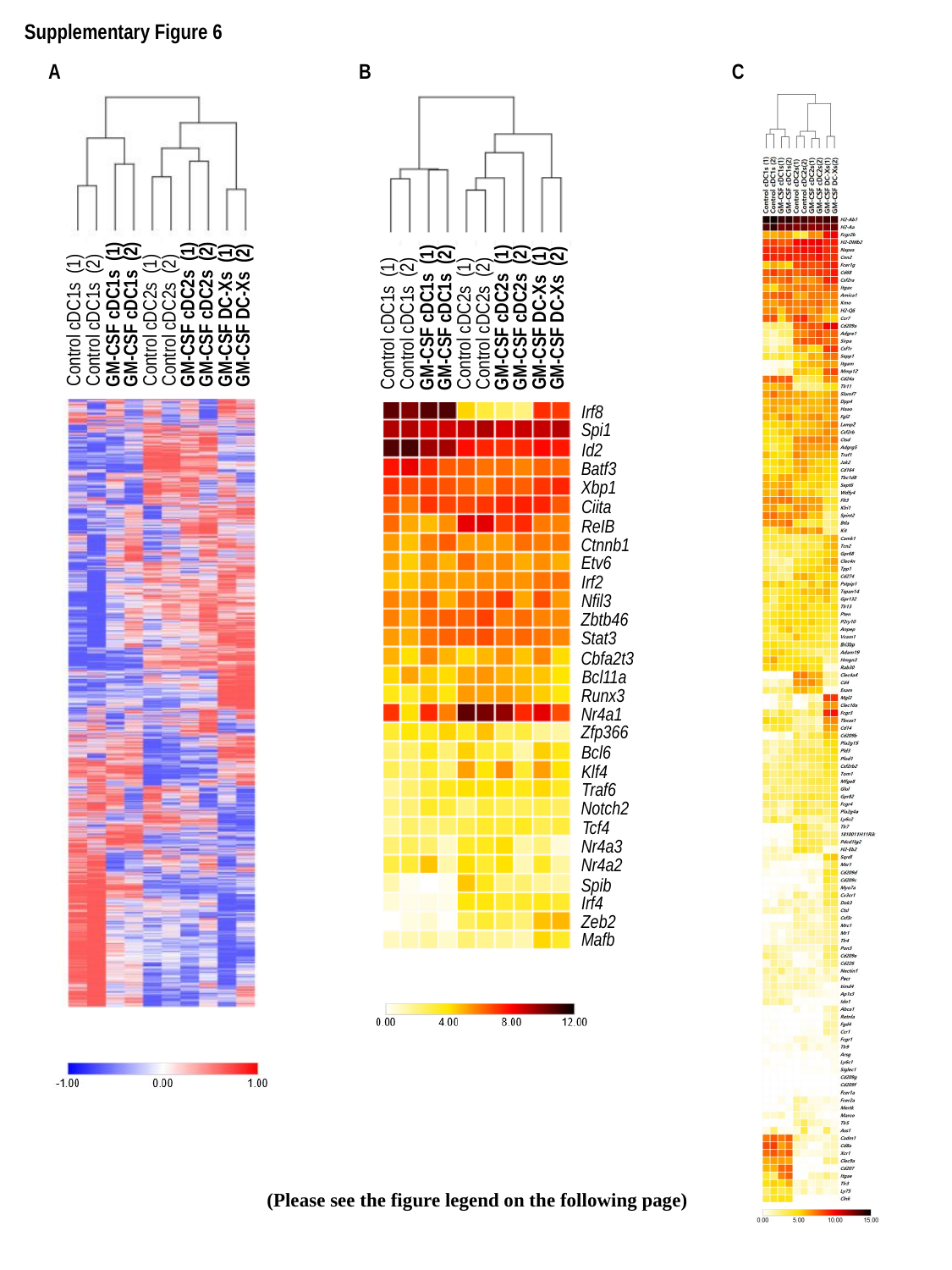

Supplementary Figure 6
A
B
C
GM-CSF cDC1s (1)
GM-CSF cDC1s (2)
GM-CSF cDC2s (1)
GM-CSF cDC2s (2)
GM-CSF DC-Xs (1)
GM-CSF DC-Xs (2)
Control cDC1s (1)
Control cDC1s (2)
Control cDC2s (1)
Control cDC2s (2)
GM-CSF cDC1s (1)
GM-CSF cDC1s (2)
GM-CSF cDC2s (1)
GM-CSF cDC2s (2)
GM-CSF DC-Xs (1)
GM-CSF DC-Xs (2)
Control cDC1s (1)
Control cDC1s (2)
Control cDC2s (1)
Control cDC2s (2)
Irf8
Spi1
Id2
Batf3
Xbp1
Ciita
ReIB
Ctnnb1
Etv6
Irf2
Nfil3
Zbtb46
Stat3
Cbfa2t3
Bcl11a
Runx3
Nr4a1
Zfp366
Bcl6
Klf4
Traf6
Notch2
Tcf4
Nr4a3
Nr4a2
Spib
Irf4
Zeb2
Mafb
(Please see the figure legend on the following page)

## Slide 7
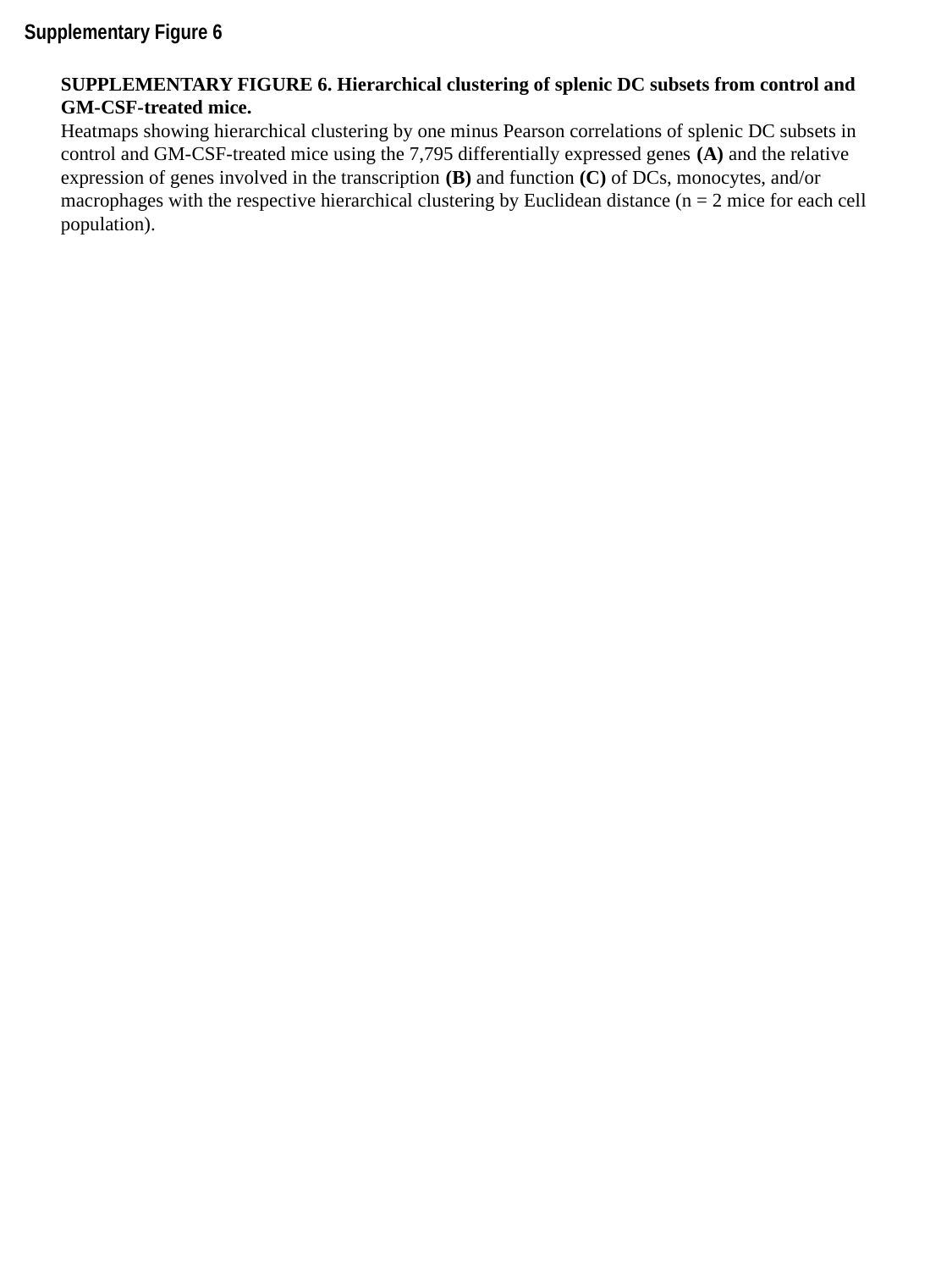

Supplementary Figure 6
SUPPLEMENTARY FIGURE 6. Hierarchical clustering of splenic DC subsets from control and GM-CSF-treated mice.
Heatmaps showing hierarchical clustering by one minus Pearson correlations of splenic DC subsets in control and GM-CSF-treated mice using the 7,795 differentially expressed genes (A) and the relative expression of genes involved in the transcription (B) and function (C) of DCs, monocytes, and/or macrophages with the respective hierarchical clustering by Euclidean distance (n = 2 mice for each cell population).

## Slide 8
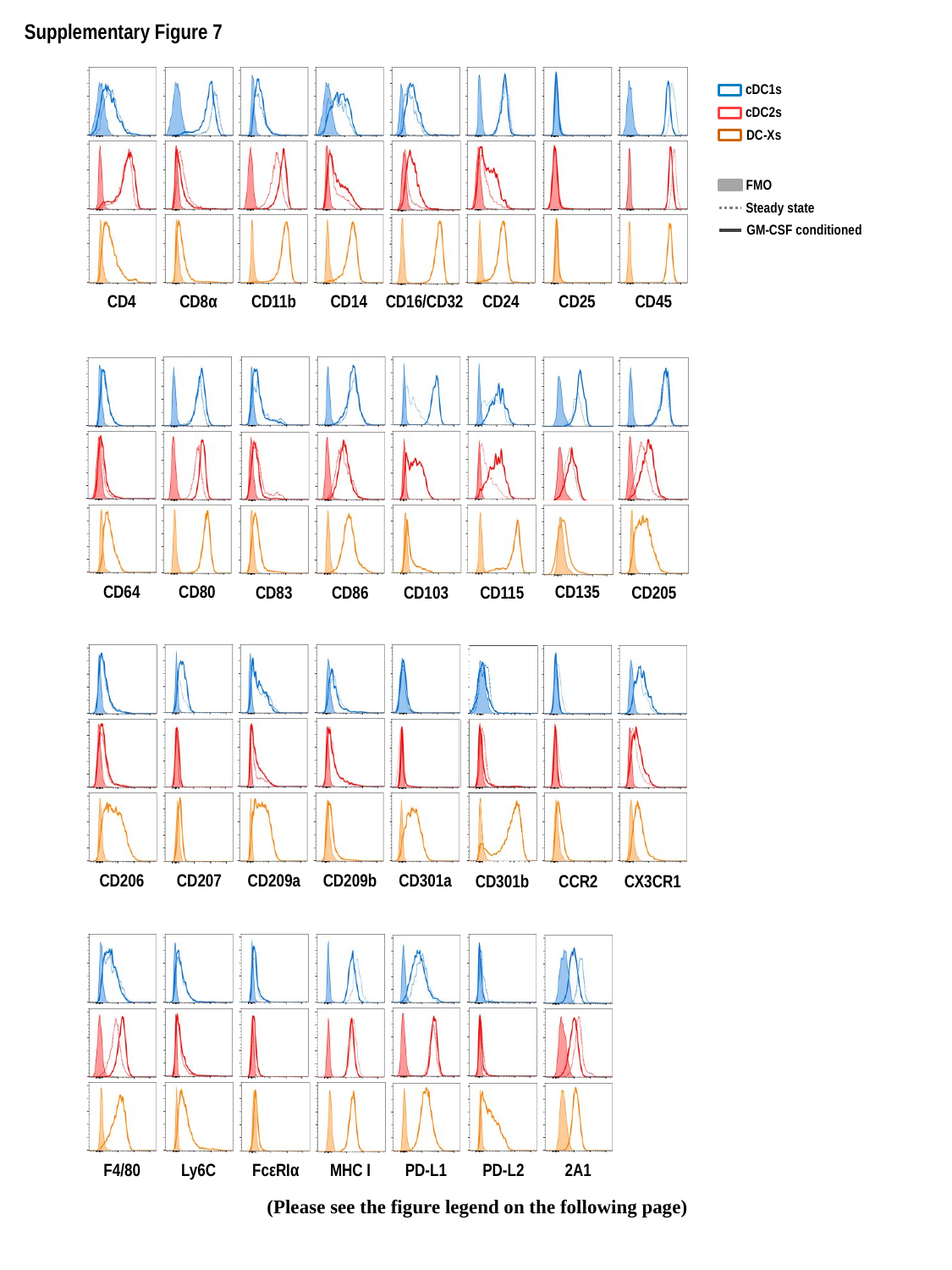

Supplementary Figure 7
cDC1s
cDC2s
DC-Xs
FMO
Steady state
GM-CSF conditioned
CD4
CD8α
CD11b
CD14
CD16/CD32
CD24
CD25
CD45
CD135
CD64
CD80
CD83
CD86
CD103
CD115
CD205
CD206
CD207
CD209a
CD209b
CD301a
CD301b
CCR2
CX3CR1
F4/80
Ly6C
FcεRIα
MHC I
PD-L1
PD-L2
2A1
(Please see the figure legend on the following page)

## Slide 9
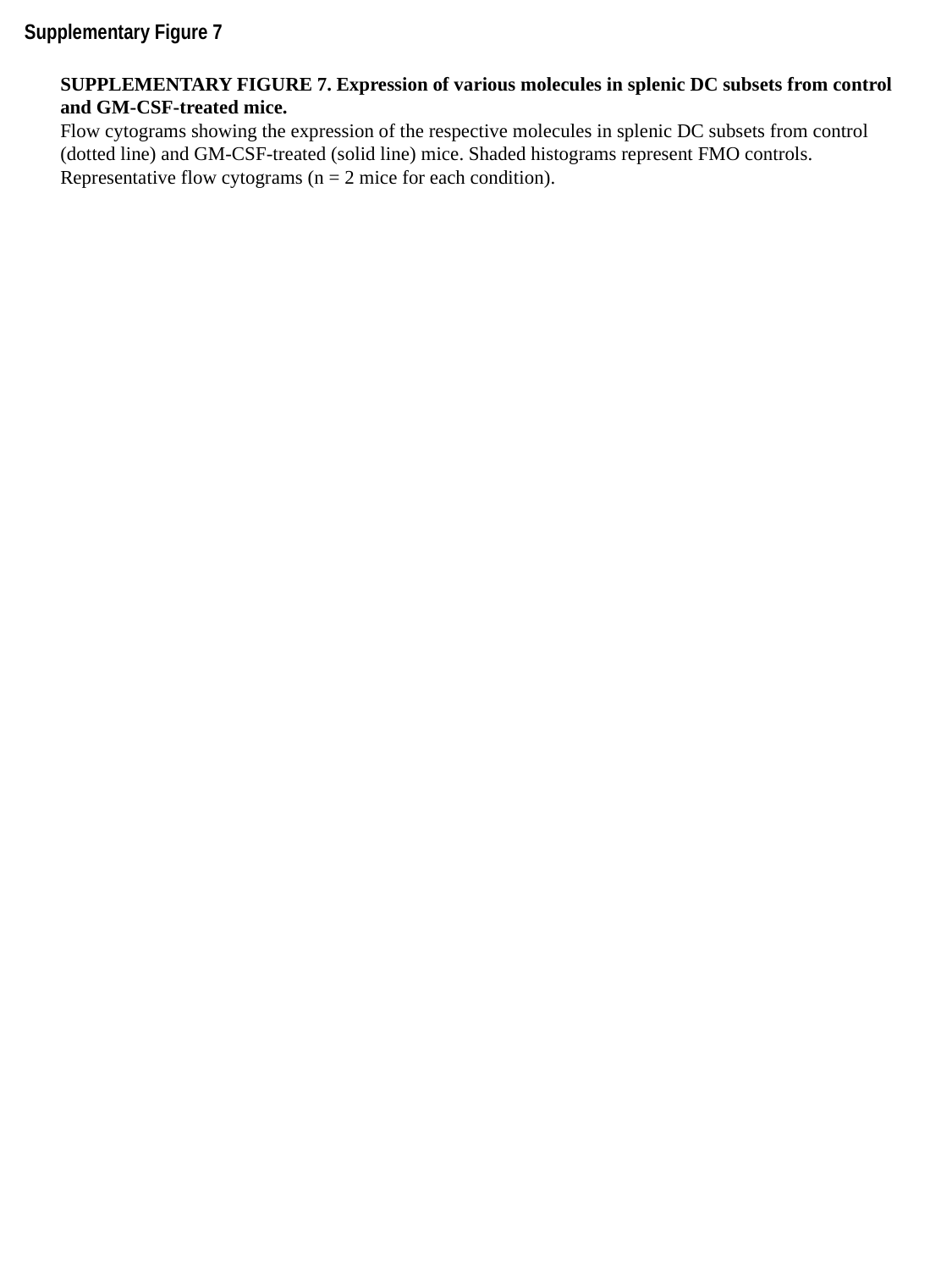

Supplementary Figure 7
SUPPLEMENTARY FIGURE 7. Expression of various molecules in splenic DC subsets from control and GM-CSF-treated mice.
Flow cytograms showing the expression of the respective molecules in splenic DC subsets from control (dotted line) and GM-CSF-treated (solid line) mice. Shaded histograms represent FMO controls. Representative flow cytograms (n = 2 mice for each condition).

## Slide 10
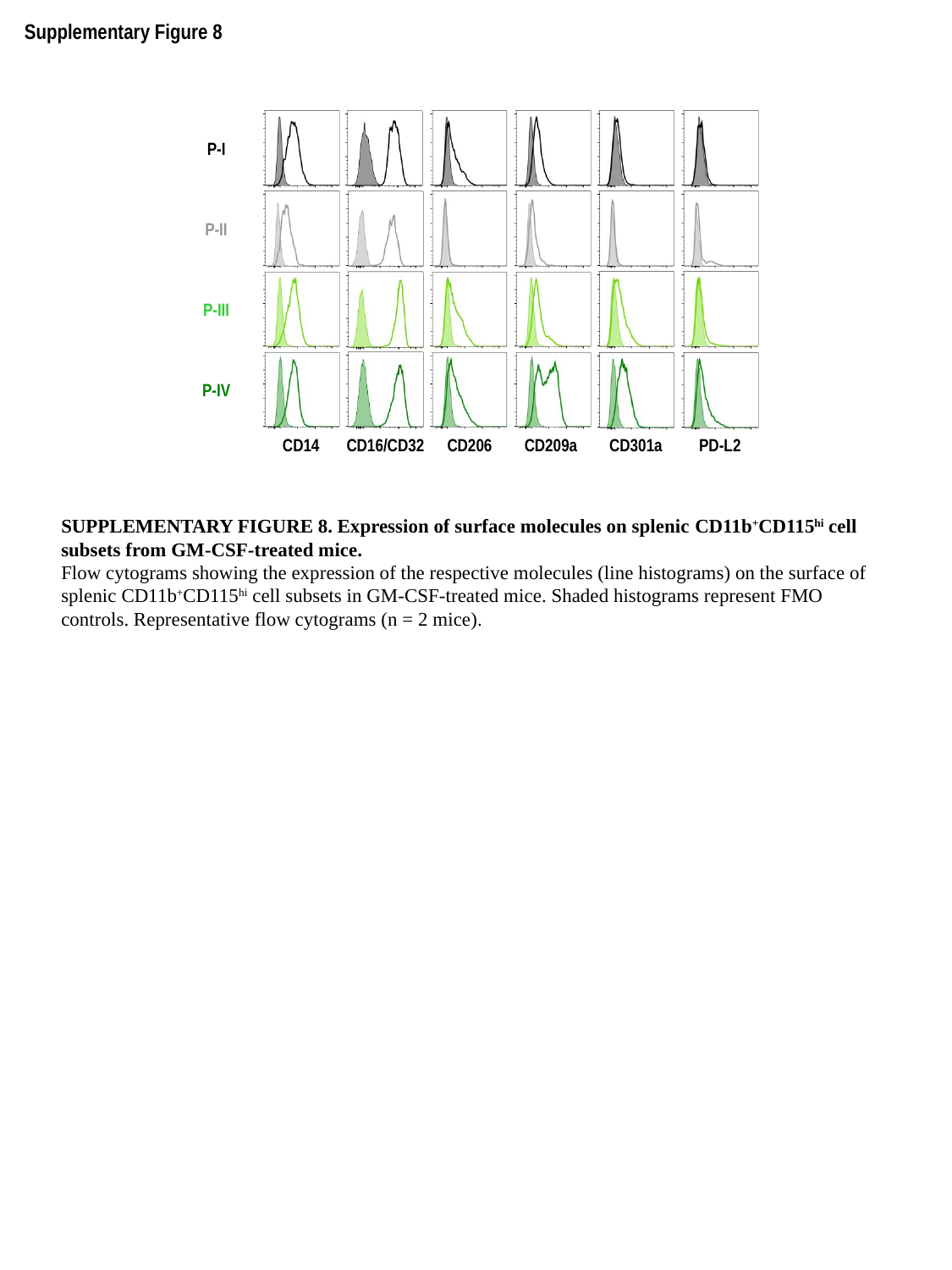

Supplementary Figure 8
P-I
P-II
P-III
P-IV
CD14
CD16/CD32
CD206
CD209a
CD301a
PD-L2
SUPPLEMENTARY FIGURE 8. Expression of surface molecules on splenic CD11b+CD115hi cell subsets from GM-CSF-treated mice.
Flow cytograms showing the expression of the respective molecules (line histograms) on the surface of splenic CD11b+CD115hi cell subsets in GM-CSF-treated mice. Shaded histograms represent FMO controls. Representative flow cytograms (n = 2 mice).

## Slide 11
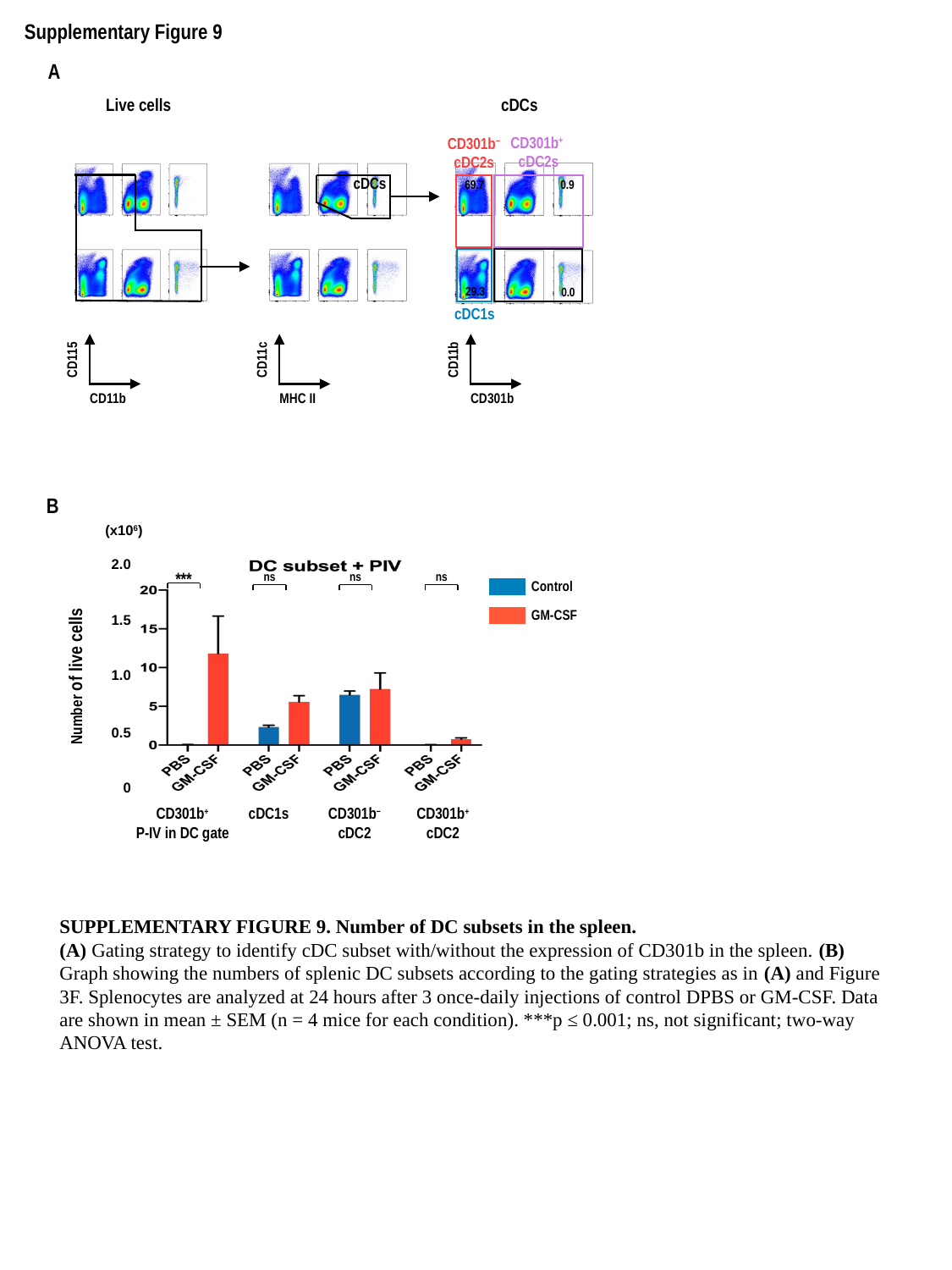

Supplementary Figure 9
A
Live cells
cDCs
CD301b+
cDC2s
CD301b−
cDC2s
69.7
0.9
29.3
0.0
cDC1s
cDCs
CD115
CD11b
CD11c
MHC II
CD11b
CD301b
B
(x106)
2.0
***
ns
ns
ns
Control
GM-CSF
1.5
Number of live cells
1.0
0.5
0
CD301b+
P-IV in DC gate
cDC1s
CD301b−
cDC2
CD301b+
cDC2
SUPPLEMENTARY FIGURE 9. Number of DC subsets in the spleen.
(A) Gating strategy to identify cDC subset with/without the expression of CD301b in the spleen. (B) Graph showing the numbers of splenic DC subsets according to the gating strategies as in (A) and Figure 3F. Splenocytes are analyzed at 24 hours after 3 once-daily injections of control DPBS or GM-CSF. Data are shown in mean ± SEM (n = 4 mice for each condition). ***p ≤ 0.001; ns, not significant; two-way ANOVA test.

## Slide 12
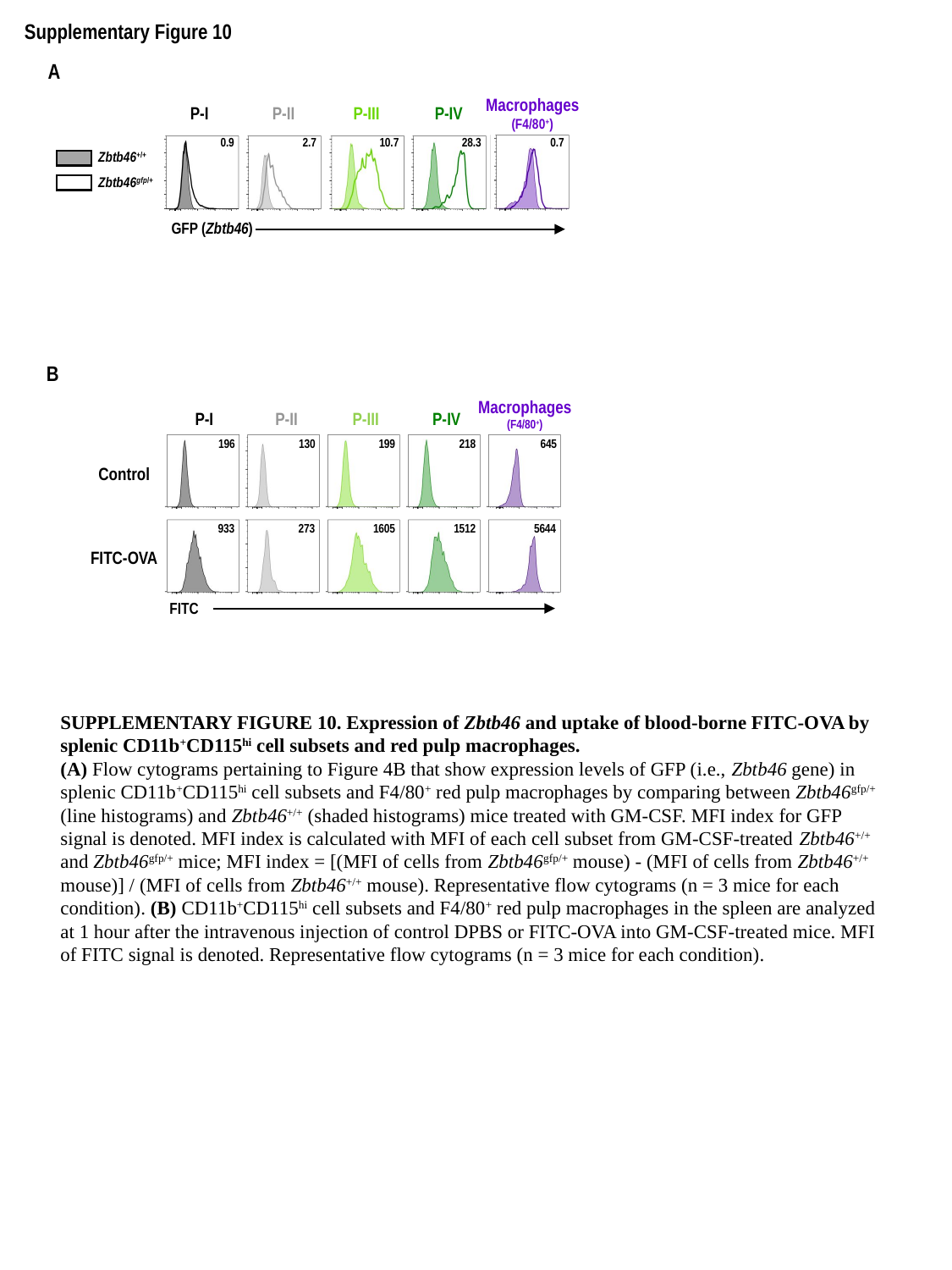

Supplementary Figure 10
A
Macrophages
(F4/80+)
P-I
P-II
P-III
P-IV
0.9
2.7
10.7
28.3
0.7
GFP (Zbtb46)
Zbtb46+/+
Zbtb46gfp/+
B
Macrophages
(F4/80+)
P-I
P-II
P-III
P-IV
196
130
199
218
645
Control
933
273
1605
1512
5644
FITC-OVA
FITC
SUPPLEMENTARY FIGURE 10. Expression of Zbtb46 and uptake of blood-borne FITC-OVA by splenic CD11b+CD115hi cell subsets and red pulp macrophages.
(A) Flow cytograms pertaining to Figure 4B that show expression levels of GFP (i.e., Zbtb46 gene) in splenic CD11b+CD115hi cell subsets and F4/80+ red pulp macrophages by comparing between Zbtb46gfp/+ (line histograms) and Zbtb46+/+ (shaded histograms) mice treated with GM-CSF. MFI index for GFP signal is denoted. MFI index is calculated with MFI of each cell subset from GM-CSF-treated Zbtb46+/+ and Zbtb46gfp/+ mice; MFI index = [(MFI of cells from Zbtb46gfp/+ mouse) - (MFI of cells from Zbtb46+/+ mouse)] / (MFI of cells from Zbtb46+/+ mouse). Representative flow cytograms (n = 3 mice for each condition). (B) CD11b+CD115hi cell subsets and F4/80+ red pulp macrophages in the spleen are analyzed at 1 hour after the intravenous injection of control DPBS or FITC-OVA into GM-CSF-treated mice. MFI of FITC signal is denoted. Representative flow cytograms (n = 3 mice for each condition).

## Slide 13
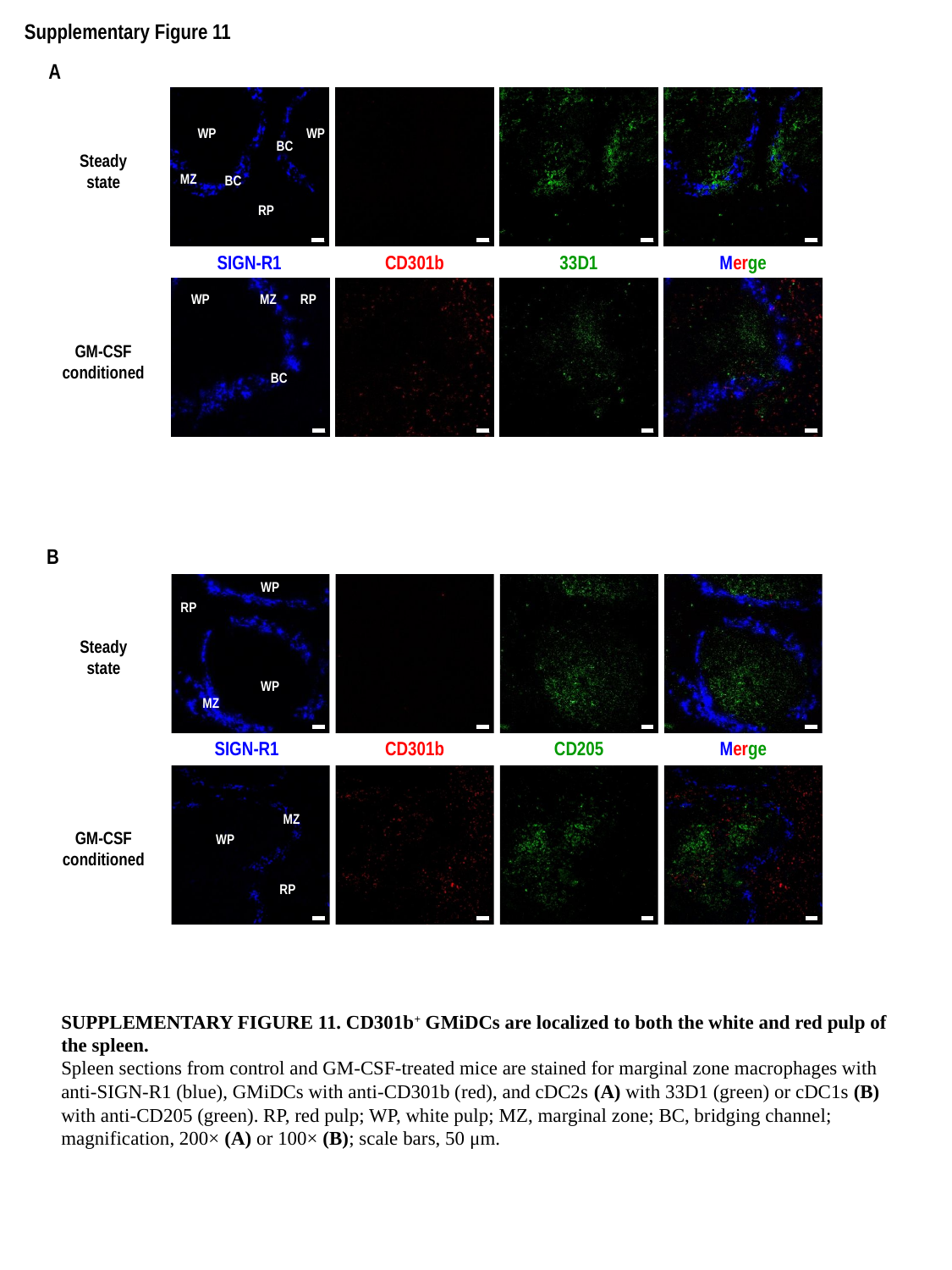

Supplementary Figure 11
A
WP
WP
BC
MZ
BC
RP
Steady
state
SIGN-R1
CD301b
33D1
Merge
WP
MZ
RP
BC
GM-CSF
conditioned
B
WP
RP
WP
MZ
Steady
state
SIGN-R1
CD301b
CD205
Merge
MZ
WP
RP
GM-CSF
conditioned
SUPPLEMENTARY FIGURE 11. CD301b+ GMiDCs are localized to both the white and red pulp of the spleen.
Spleen sections from control and GM-CSF-treated mice are stained for marginal zone macrophages with anti-SIGN-R1 (blue), GMiDCs with anti-CD301b (red), and cDC2s (A) with 33D1 (green) or cDC1s (B) with anti-CD205 (green). RP, red pulp; WP, white pulp; MZ, marginal zone; BC, bridging channel; magnification, 200× (A) or 100× (B); scale bars, 50 μm.

## Slide 14
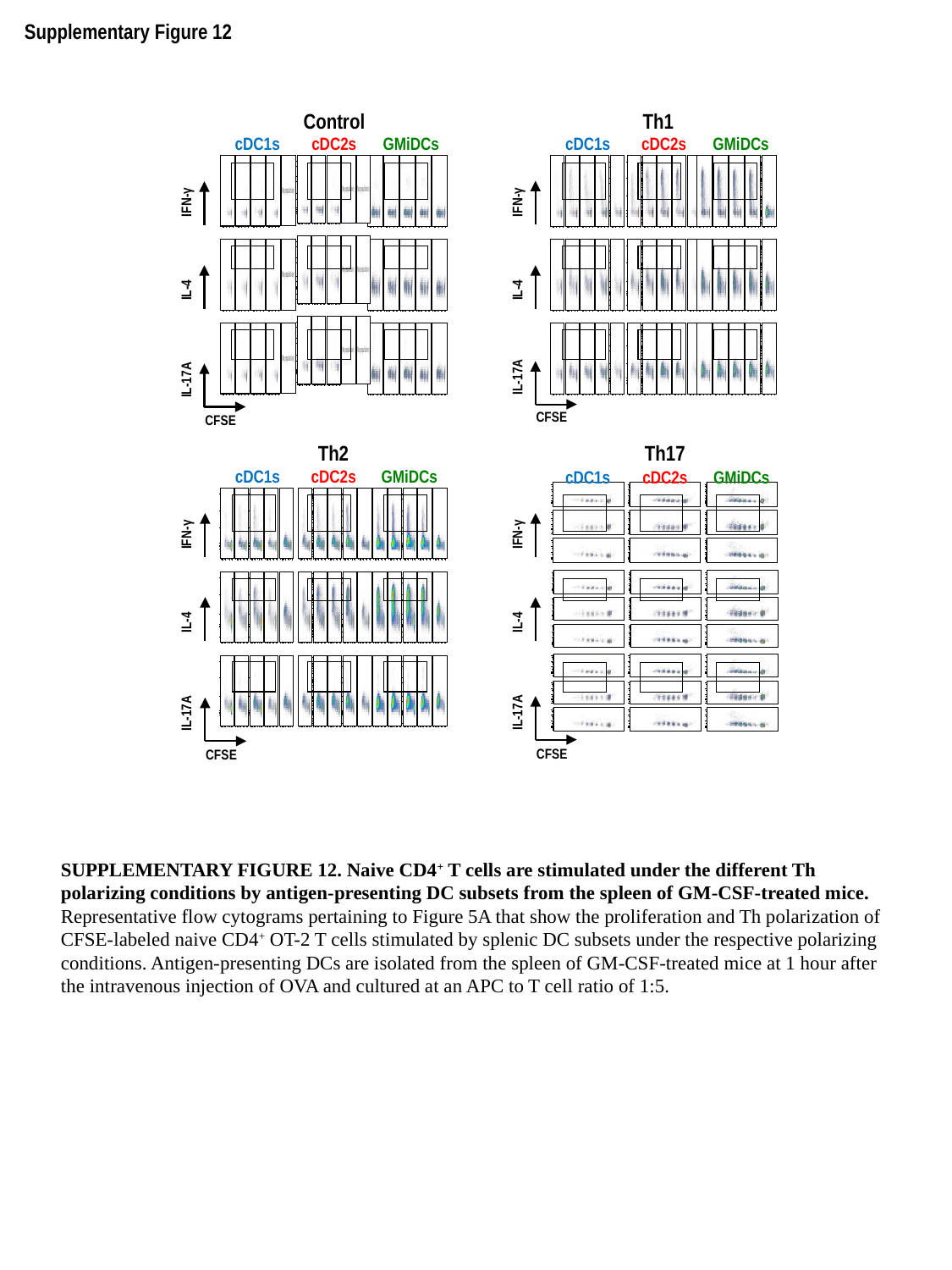

Supplementary Figure 12
Control
cDC1s
cDC2s
GMiDCs
IFN-γ
IL-4
IL-17A
CFSE
Th1
cDC1s
cDC2s
GMiDCs
IFN-γ
IL-4
IL-17A
CFSE
Th2
cDC1s
cDC2s
GMiDCs
IFN-γ
IL-4
IL-17A
CFSE
Th17
cDC1s
cDC2s
GMiDCs
IFN-γ
IL-4
IL-17A
CFSE
SUPPLEMENTARY FIGURE 12. Naive CD4+ T cells are stimulated under the different Th polarizing conditions by antigen-presenting DC subsets from the spleen of GM-CSF-treated mice.
Representative flow cytograms pertaining to Figure 5A that show the proliferation and Th polarization of CFSE-labeled naive CD4+ OT-2 T cells stimulated by splenic DC subsets under the respective polarizing conditions. Antigen-presenting DCs are isolated from the spleen of GM-CSF-treated mice at 1 hour after the intravenous injection of OVA and cultured at an APC to T cell ratio of 1:5.

## Slide 15
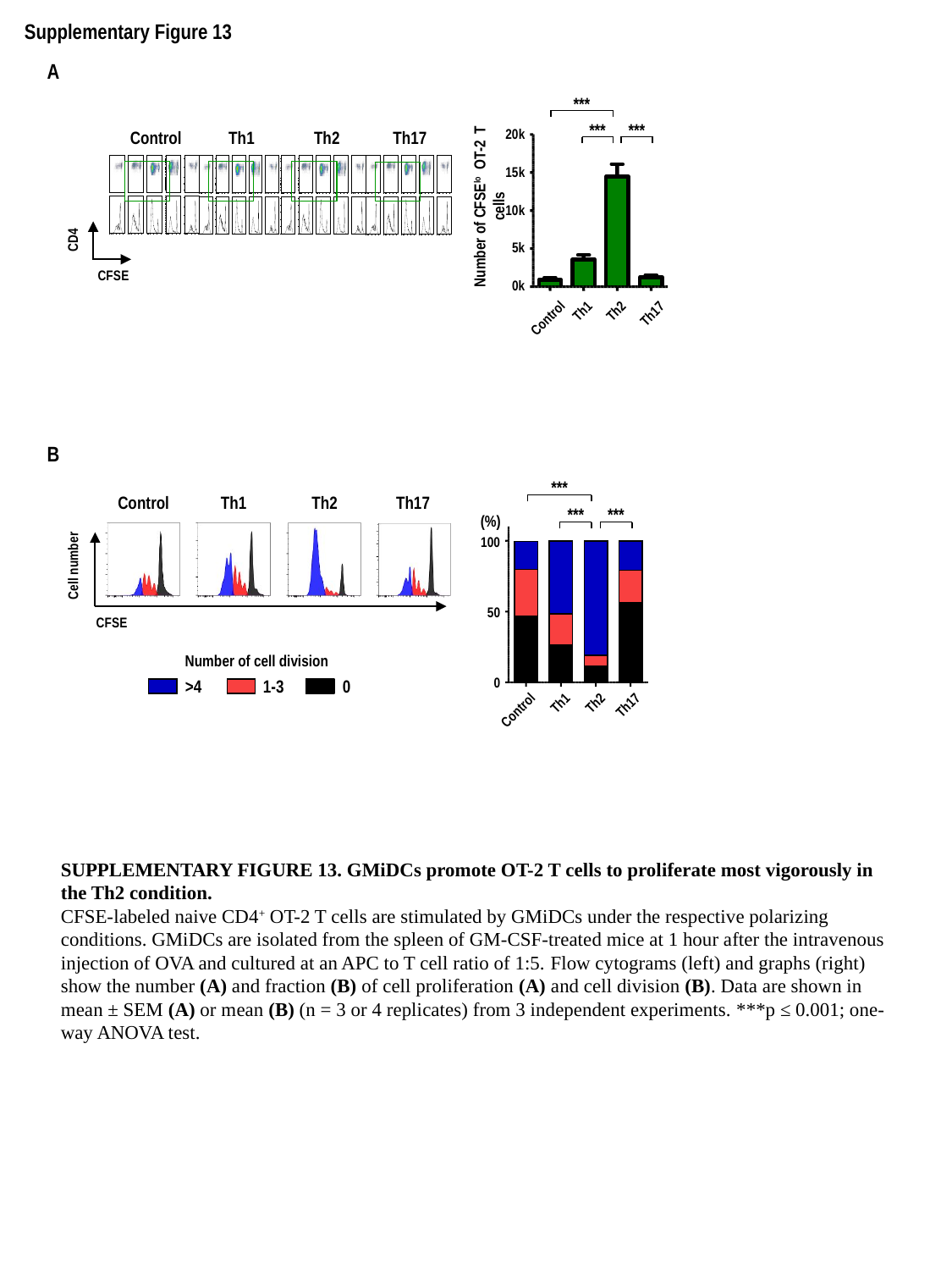

Supplementary Figure 13
A
***
Number of CFSElo OT-2 T cells
***
***
20k
15k
10k
5k
0k
Th1
Th2
Th17
Control
Control
Th1
Th2
Th17
CD4
CFSE
B
***
***
***
(%)
100
50
0
Th1
Th2
Th17
Control
Control
Th1
Th2
Th17
Cell number
CFSE
Number of cell division
1-3
0
>4
SUPPLEMENTARY FIGURE 13. GMiDCs promote OT-2 T cells to proliferate most vigorously in the Th2 condition.
CFSE-labeled naive CD4+ OT-2 T cells are stimulated by GMiDCs under the respective polarizing conditions. GMiDCs are isolated from the spleen of GM-CSF-treated mice at 1 hour after the intravenous injection of OVA and cultured at an APC to T cell ratio of 1:5. Flow cytograms (left) and graphs (right) show the number (A) and fraction (B) of cell proliferation (A) and cell division (B). Data are shown in mean ± SEM (A) or mean (B) (n = 3 or 4 replicates) from 3 independent experiments. ***p ≤ 0.001; one-way ANOVA test.

## Slide 16
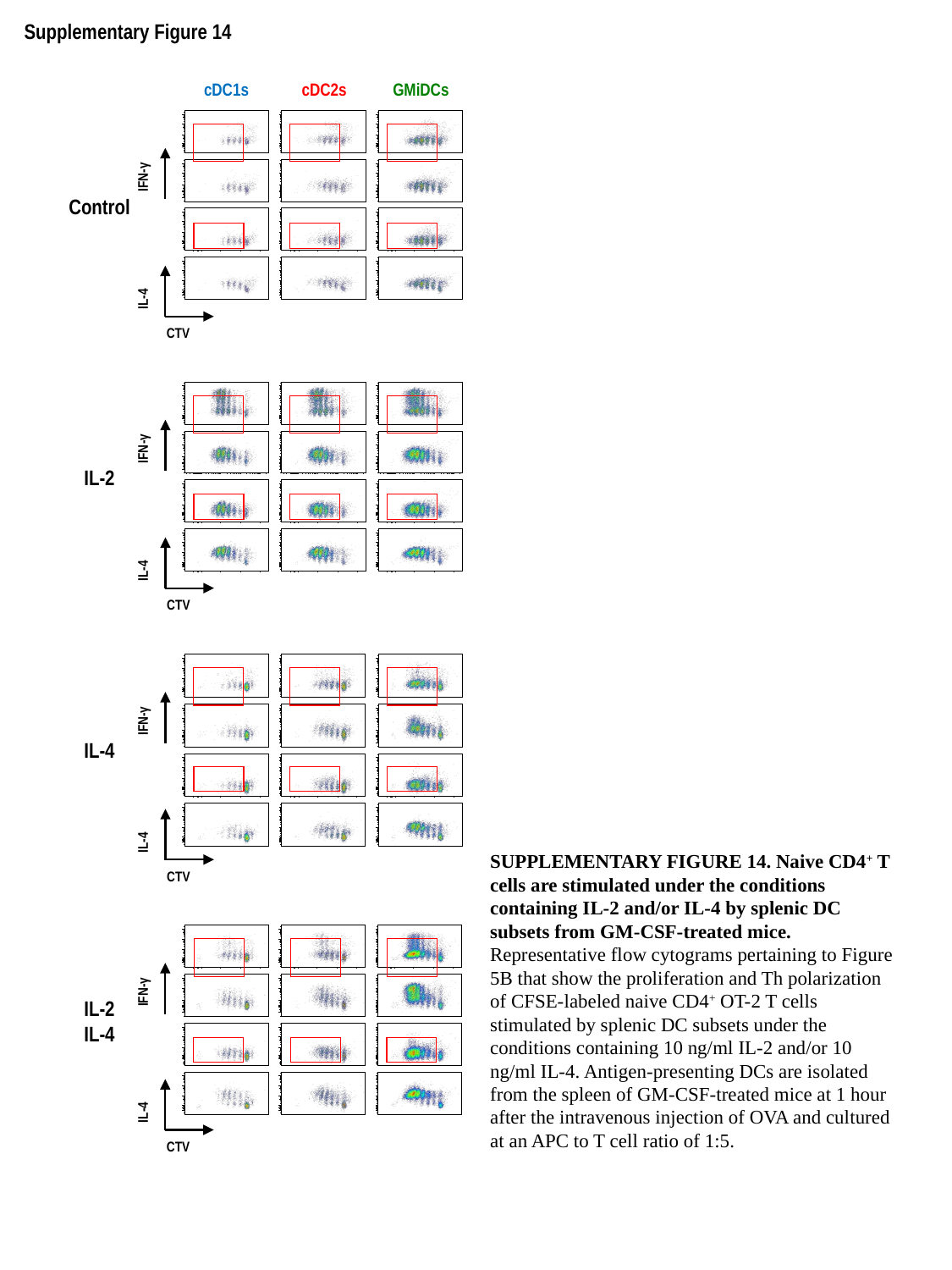

Supplementary Figure 14
cDC1s
cDC2s
GMiDCs
IFN-γ
Control
IL-4
CTV
IFN-γ
IL-2
IL-4
CTV
IFN-γ
IL-4
IL-4
CTV
IFN-γ
IL-2
IL-4
IL-4
CTV
SUPPLEMENTARY FIGURE 14. Naive CD4+ T cells are stimulated under the conditions containing IL-2 and/or IL-4 by splenic DC subsets from GM-CSF-treated mice.
Representative flow cytograms pertaining to Figure 5B that show the proliferation and Th polarization of CFSE-labeled naive CD4+ OT-2 T cells stimulated by splenic DC subsets under the conditions containing 10 ng/ml IL-2 and/or 10 ng/ml IL-4. Antigen-presenting DCs are isolated from the spleen of GM-CSF-treated mice at 1 hour after the intravenous injection of OVA and cultured at an APC to T cell ratio of 1:5.

## Slide 17
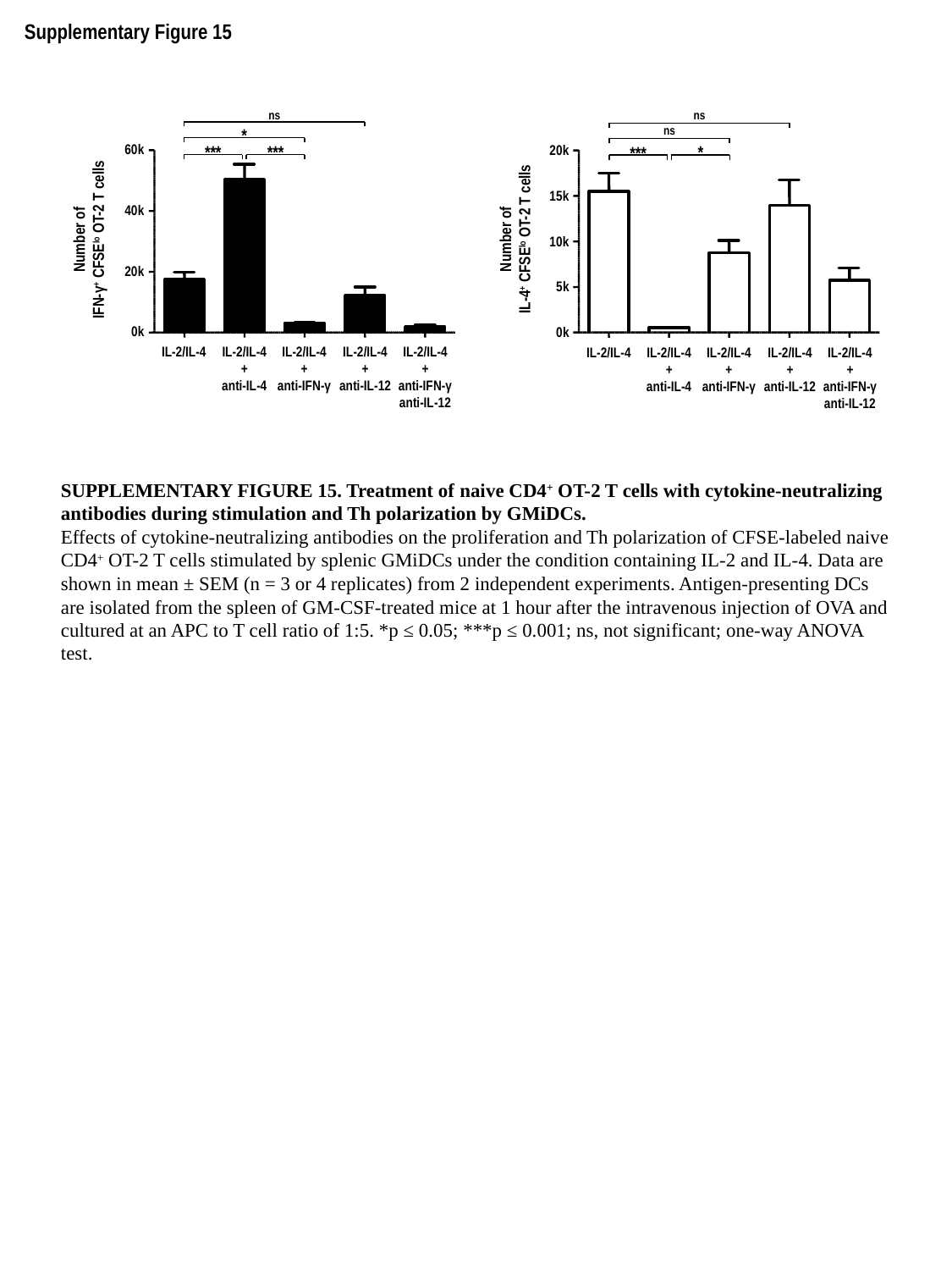

Supplementary Figure 15
ns
*
***
***
60k
40k
20k
0k
Number of
IFN-γ+ CFSElo OT-2 T cells
IL-2/IL-4
IL-2/IL-4
+
anti-IL-4
IL-2/IL-4
+
anti-IFN-γ
IL-2/IL-4
+
anti-IL-12
IL-2/IL-4
+
anti-IFN-γ
anti-IL-12
ns
ns
*
***
20k
15k
10k
5k
0k
Number of
IL-4+ CFSElo OT-2 T cells
IL-2/IL-4
IL-2/IL-4
+
anti-IL-4
IL-2/IL-4
+
anti-IFN-γ
IL-2/IL-4
+
anti-IL-12
IL-2/IL-4
+
anti-IFN-γ
anti-IL-12
SUPPLEMENTARY FIGURE 15. Treatment of naive CD4+ OT-2 T cells with cytokine-neutralizing antibodies during stimulation and Th polarization by GMiDCs.
Effects of cytokine-neutralizing antibodies on the proliferation and Th polarization of CFSE-labeled naive CD4+ OT-2 T cells stimulated by splenic GMiDCs under the condition containing IL-2 and IL-4. Data are shown in mean ± SEM (n = 3 or 4 replicates) from 2 independent experiments. Antigen-presenting DCs are isolated from the spleen of GM-CSF-treated mice at 1 hour after the intravenous injection of OVA and cultured at an APC to T cell ratio of 1:5. *p ≤ 0.05; ***p ≤ 0.001; ns, not significant; one-way ANOVA test.

## Slide 18
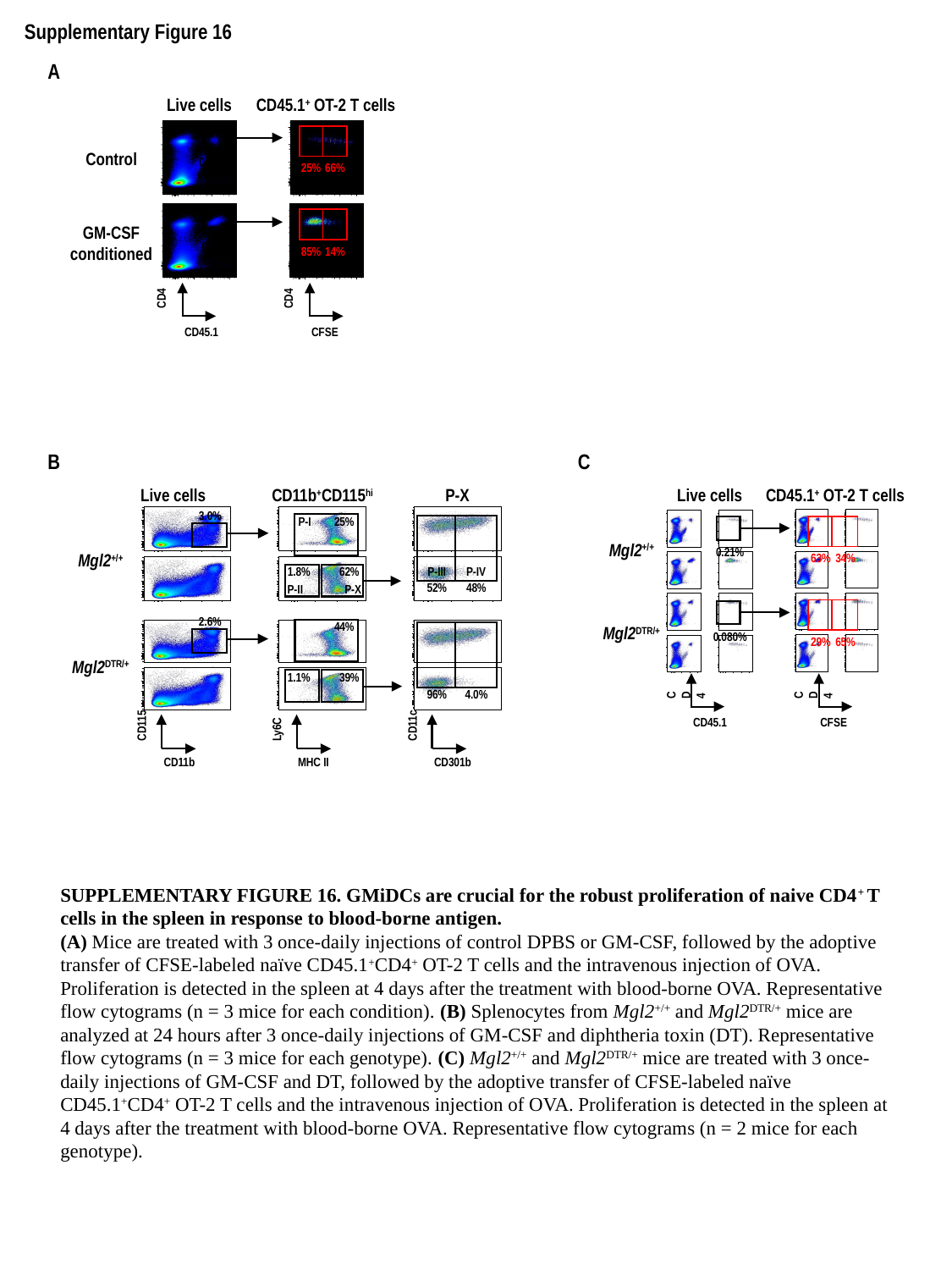

Supplementary Figure 16
A
Live cells
CD45.1+ OT-2 T cells
26.0%
Control
0.096%
25%
66%
85.9%
GM-CSF
conditioned
0.55%
85%
14%
CD4
CD45.1
CD4
CFSE
B
C
Live cells
CD11b+CD115hi
P-X
P-I
Mgl2+/+
P-III
P-IV
P-II
P-X
Mgl2DTR/+
CD11c
CD301b
CD115
CD11b
Ly6C
MHC II
Live cells
CD45.1+ OT-2 T cells
Mgl2+/+
0.21%
63%
34%
Mgl2DTR/+
0.080%
29%
65%
CD4
CD45.1
CD4
CFSE
3.0%
25%
62%
1.8%
52%
48%
2.6%
44%
39%
1.1%
96%
4.0%
SUPPLEMENTARY FIGURE 16. GMiDCs are crucial for the robust proliferation of naive CD4+ T cells in the spleen in response to blood-borne antigen.
(A) Mice are treated with 3 once-daily injections of control DPBS or GM-CSF, followed by the adoptive transfer of CFSE-labeled naïve CD45.1+CD4+ OT-2 T cells and the intravenous injection of OVA. Proliferation is detected in the spleen at 4 days after the treatment with blood-borne OVA. Representative flow cytograms (n = 3 mice for each condition). (B) Splenocytes from Mgl2+/+ and Mgl2DTR/+ mice are analyzed at 24 hours after 3 once-daily injections of GM-CSF and diphtheria toxin (DT). Representative flow cytograms (n = 3 mice for each genotype). (C) Mgl2+/+ and Mgl2DTR/+ mice are treated with 3 once-daily injections of GM-CSF and DT, followed by the adoptive transfer of CFSE-labeled naïve CD45.1+CD4+ OT-2 T cells and the intravenous injection of OVA. Proliferation is detected in the spleen at 4 days after the treatment with blood-borne OVA. Representative flow cytograms (n = 2 mice for each genotype).

## Slide 19
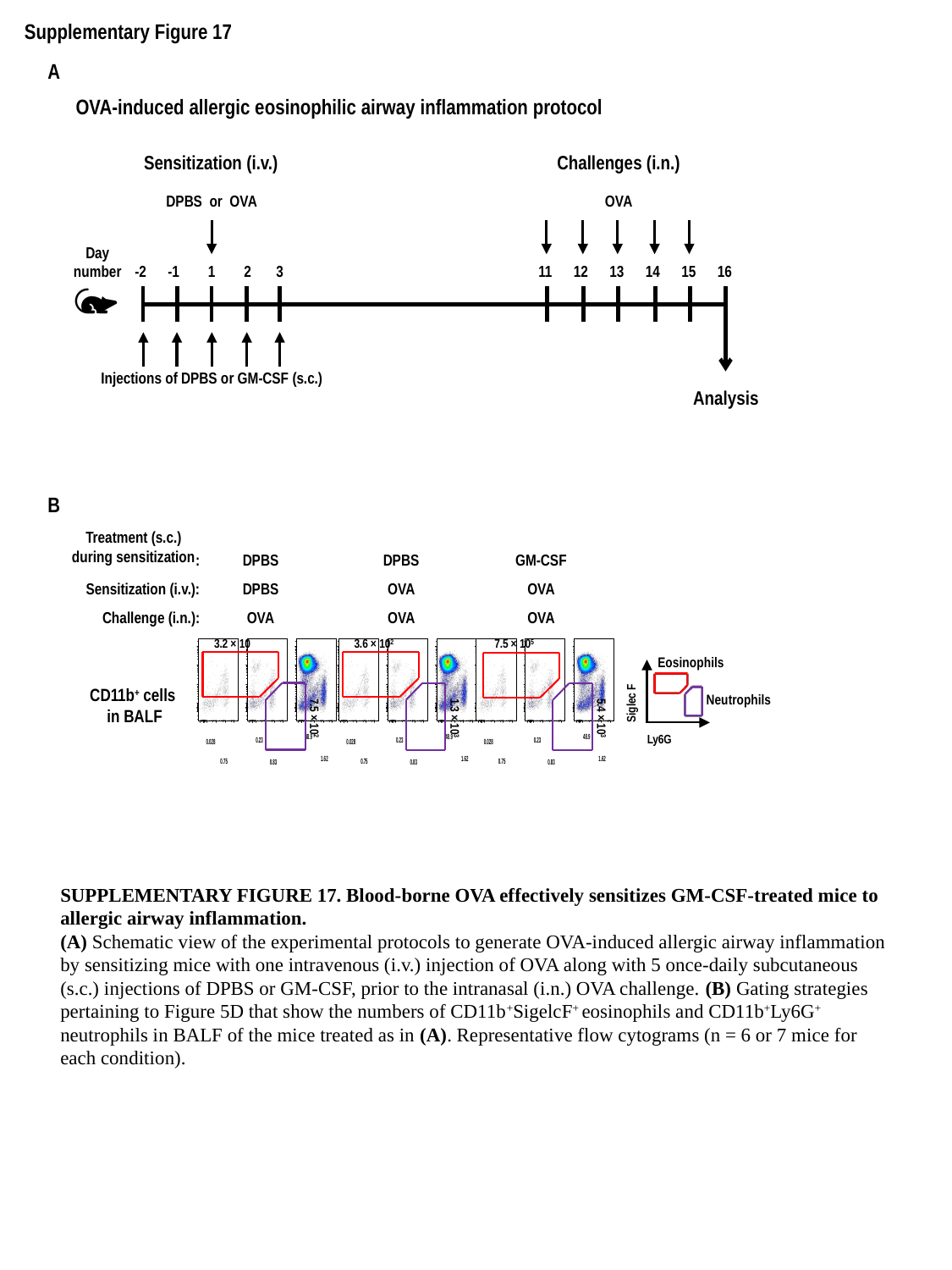

Supplementary Figure 17
A
OVA-induced allergic eosinophilic airway inflammation protocol
Sensitization (i.v.)
Challenges (i.n.)
DPBS or OVA
OVA
Day
number
-2 -1 1 2 3
11 12 13 14 15 16
Injections of DPBS or GM-CSF (s.c.)
Analysis
B
Treatment (s.c.)
during sensitization
:
Sensitization (i.v.):
Challenge (i.n.):
DPBS
DPBS
OVA
DPBS
OVA
OVA
GM-CSF
OVA
OVA
3.2 × 10
3.6 × 10^2^
7.5 × 10^5^
Eosinophils
Neutrophils
Siglec F
Ly6G
7.5 × 10^2^
1.3 × 10^3^
5.4 × 10^3^
CD11b+ cells
in BALF
SUPPLEMENTARY FIGURE 17. Blood-borne OVA effectively sensitizes GM-CSF-treated mice to allergic airway inflammation.
(A) Schematic view of the experimental protocols to generate OVA-induced allergic airway inflammation by sensitizing mice with one intravenous (i.v.) injection of OVA along with 5 once-daily subcutaneous (s.c.) injections of DPBS or GM-CSF, prior to the intranasal (i.n.) OVA challenge. (B) Gating strategies pertaining to Figure 5D that show the numbers of CD11b+SigelcF+ eosinophils and CD11b+Ly6G+ neutrophils in BALF of the mice treated as in (A). Representative flow cytograms (n = 6 or 7 mice for each condition).

## Slide 20
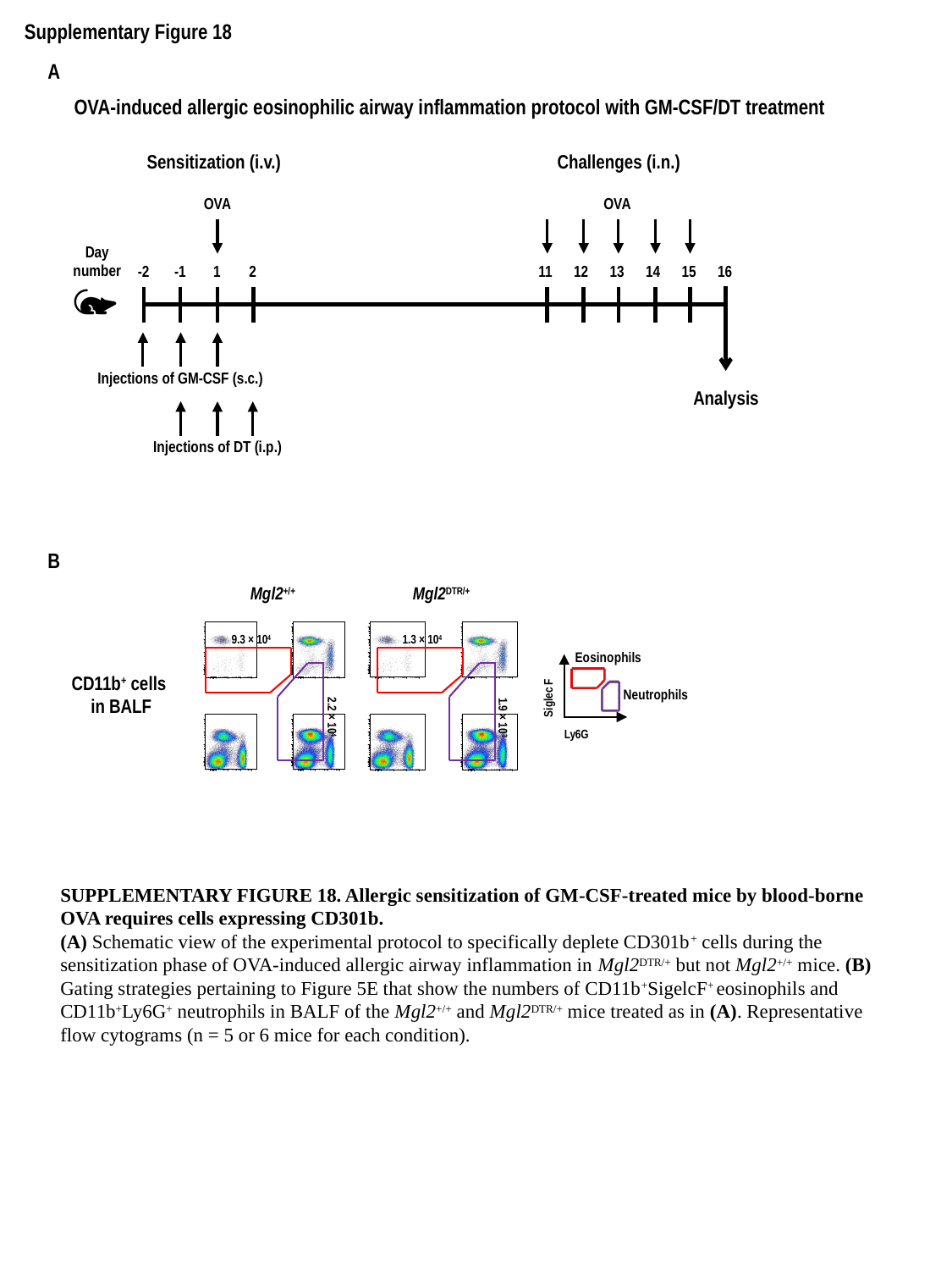

Supplementary Figure 18
A
OVA-induced allergic eosinophilic airway inflammation protocol with GM-CSF/DT treatment
Sensitization (i.v.)
Challenges (i.n.)
OVA
OVA
Day
number
-2
-1
1
2
11 12 13 14 15 16
Injections of GM-CSF (s.c.)
Analysis
Injections of DT (i.p.)
B
Mgl2+/+
Mgl2DTR/+
1.3 × 10^4^
1.9 × 10^3^
9.3 × 10^4^
2.2 × 10^4^
Eosinophils
Neutrophils
Siglec F
Ly6G
CD11b+ cells
in BALF
SUPPLEMENTARY FIGURE 18. Allergic sensitization of GM-CSF-treated mice by blood-borne OVA requires cells expressing CD301b.
(A) Schematic view of the experimental protocol to specifically deplete CD301b+ cells during the sensitization phase of OVA-induced allergic airway inflammation in Mgl2DTR/+ but not Mgl2+/+ mice. (B) Gating strategies pertaining to Figure 5E that show the numbers of CD11b+SigelcF+ eosinophils and CD11b+Ly6G+ neutrophils in BALF of the Mgl2+/+ and Mgl2DTR/+ mice treated as in (A). Representative flow cytograms (n = 5 or 6 mice for each condition).

## Slide 21
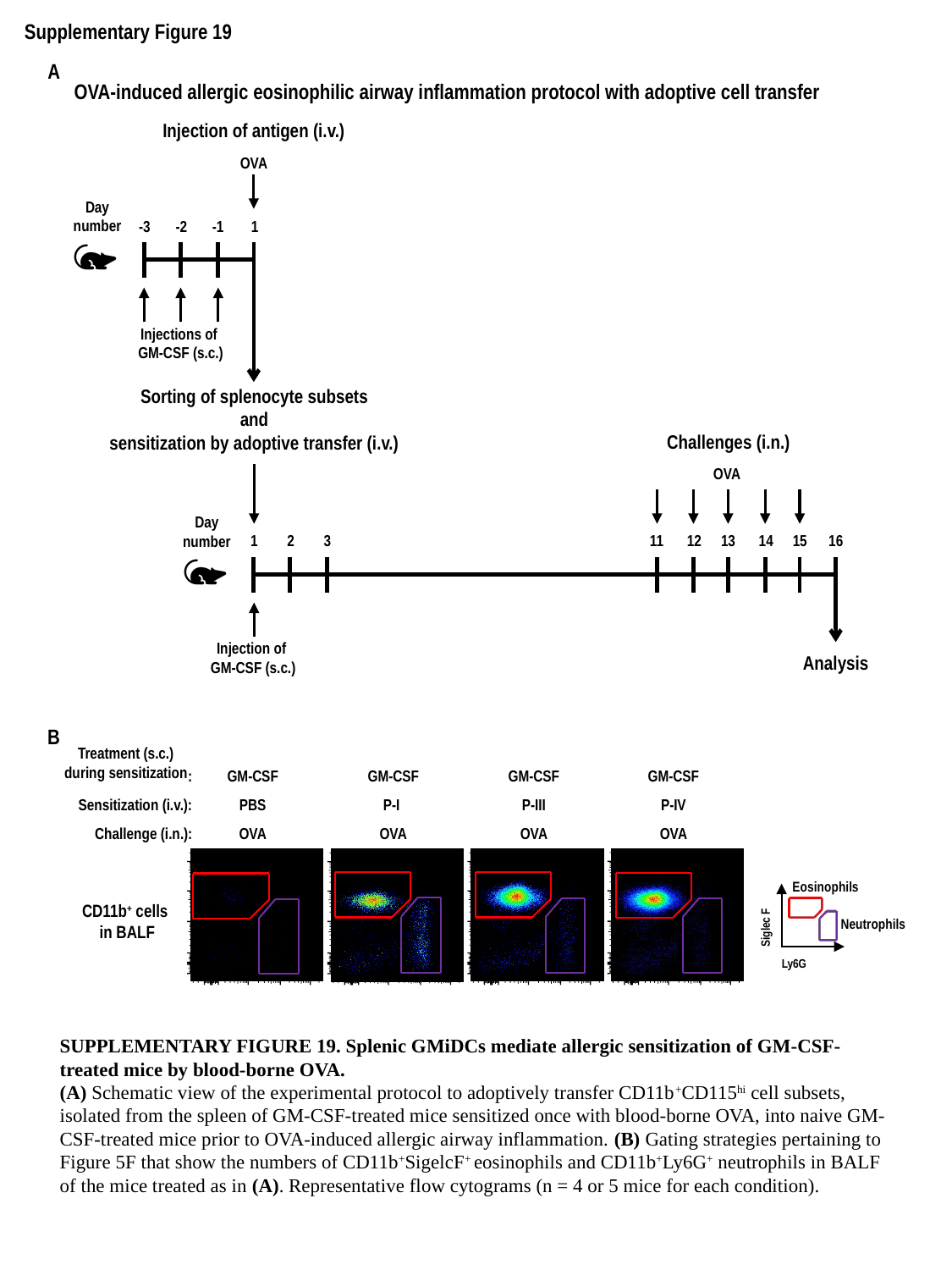

Supplementary Figure 19
A
OVA-induced allergic eosinophilic airway inflammation protocol with adoptive cell transfer
Injection of antigen (i.v.)
OVA
Day
number
-3
-2
-1
1
Injections of
GM-CSF (s.c.)
Sorting of splenocyte subsets
and
sensitization by adoptive transfer (i.v.)
Challenges (i.n.)
OVA
Day
number
1
2
3
11
12
13
14
15
16
Injection of
GM-CSF (s.c.)
Analysis
B
Treatment (s.c.)
during sensitization
:
Sensitization (i.v.):
Challenge (i.n.):
GM-CSF
PBS
OVA
GM-CSF
P-I
OVA
GM-CSF
P-III
OVA
GM-CSF
P-IV
OVA
6.5 × 10^2^
3.8 × 10^4^
3.9 × 10^5^
6.8 × 10^5^
CD11b+ cells
in BALF
6.0 × 10
1.2 × 10^4^
1.2 × 10^4^
4.9 × 10^3^
Eosinophils
Neutrophils
Siglec F
Ly6G
SUPPLEMENTARY FIGURE 19. Splenic GMiDCs mediate allergic sensitization of GM-CSF-treated mice by blood-borne OVA.
(A) Schematic view of the experimental protocol to adoptively transfer CD11b+CD115hi cell subsets, isolated from the spleen of GM-CSF-treated mice sensitized once with blood-borne OVA, into naive GM-CSF-treated mice prior to OVA-induced allergic airway inflammation. (B) Gating strategies pertaining to Figure 5F that show the numbers of CD11b+SigelcF+ eosinophils and CD11b+Ly6G+ neutrophils in BALF of the mice treated as in (A). Representative flow cytograms (n = 4 or 5 mice for each condition).

## Slide 22
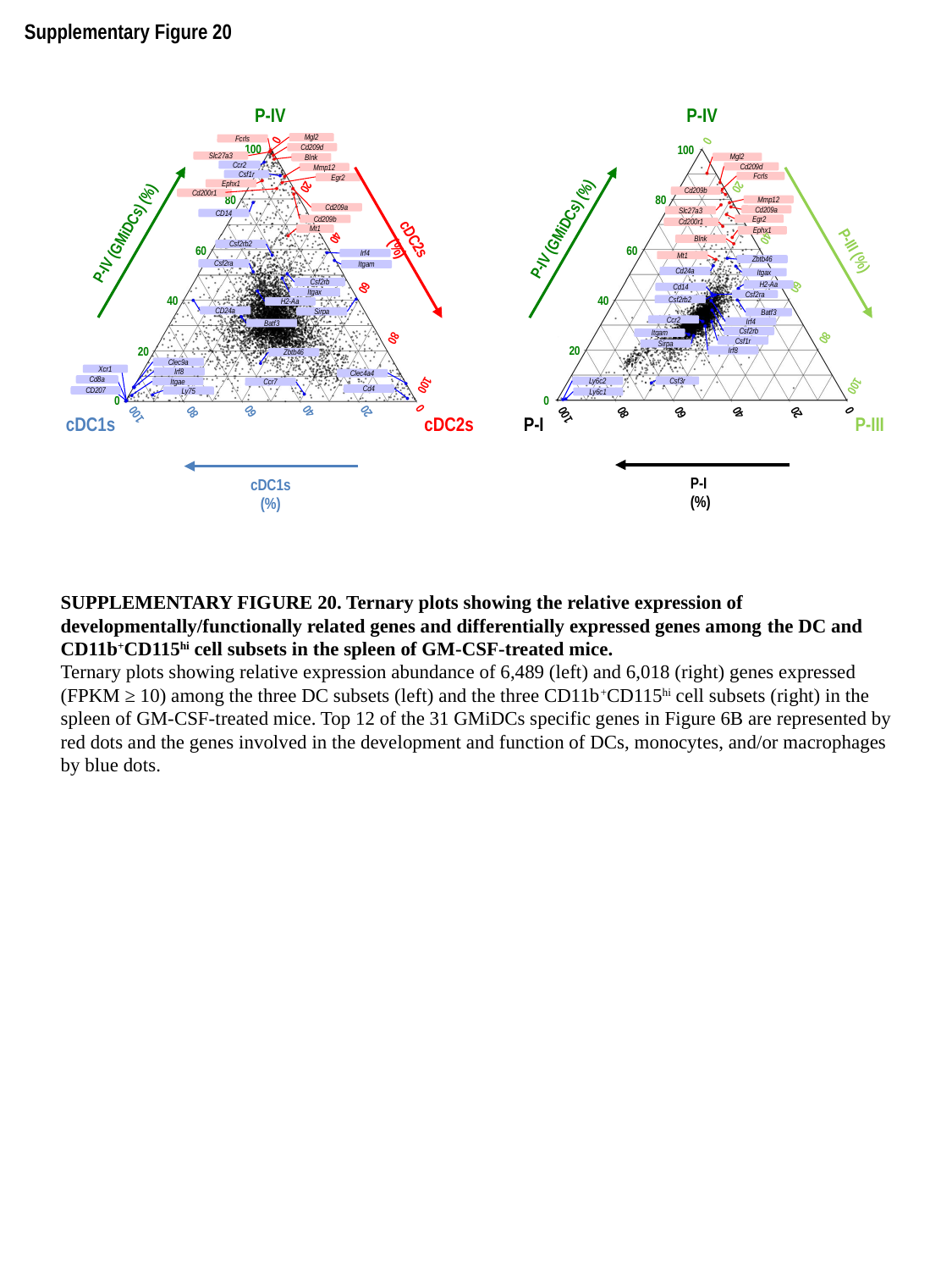

Supplementary Figure 20
P-IV
0
Mgl2
Fcrls
100
Cd209d
Slc27a3
Blnk
Ccr2
Mmp12
Csf1r
Egr2
20
Ephx1
80
Cd200r1
Cd209a
CD14
Cd209b
P-IV (GMiDCs) (%)
cDC2s (%)
Mt1
40
60
Csf2rb2
Irf4
Csf2ra
Itgam
60
Csf2rb
40
Itgax
H2-Aa
CD24a
Sirpa
Batf3
80
20
Zbtb46
Clec9a
Xcr1
Irf8
Clec4a4
100
Cd8a
Itgae
Ccr7
Cd4
CD207
Ly75
0
0
20
40
60
80
100
cDC1s
cDC2s
cDC1s (%)
P-IV
0
100
Mgl2
Cd209d
Fcrls
20
80
Cd209b
Mmp12
Cd209a
Slc27a3
P-IV (GMiDCs) (%)
Egr2
Cd200r1
40
Ephx1
P-III (%)
Blnk
60
Mt1
Zbtb46
Cd24a
Itgax
60
H2-Aa
Cd14
40
Csf2ra
Csf2rb2
Batf3
Ccr2
Irf4
80
Csf2rb
Itgam
Csf1r
20
Sirpa
Irf8
100
Csf3r
Ly6c2
0
Ly6c1
0
80
60
40
20
100
P-I
P-III
P-I (%)
SUPPLEMENTARY FIGURE 20. Ternary plots showing the relative expression of developmentally/functionally related genes and differentially expressed genes among the DC and CD11b+CD115hi cell subsets in the spleen of GM-CSF-treated mice.
Ternary plots showing relative expression abundance of 6,489 (left) and 6,018 (right) genes expressed (FPKM ≥ 10) among the three DC subsets (left) and the three CD11b+CD115hi cell subsets (right) in the spleen of GM-CSF-treated mice. Top 12 of the 31 GMiDCs specific genes in Figure 6B are represented by red dots and the genes involved in the development and function of DCs, monocytes, and/or macrophages by blue dots.

## Slide 23
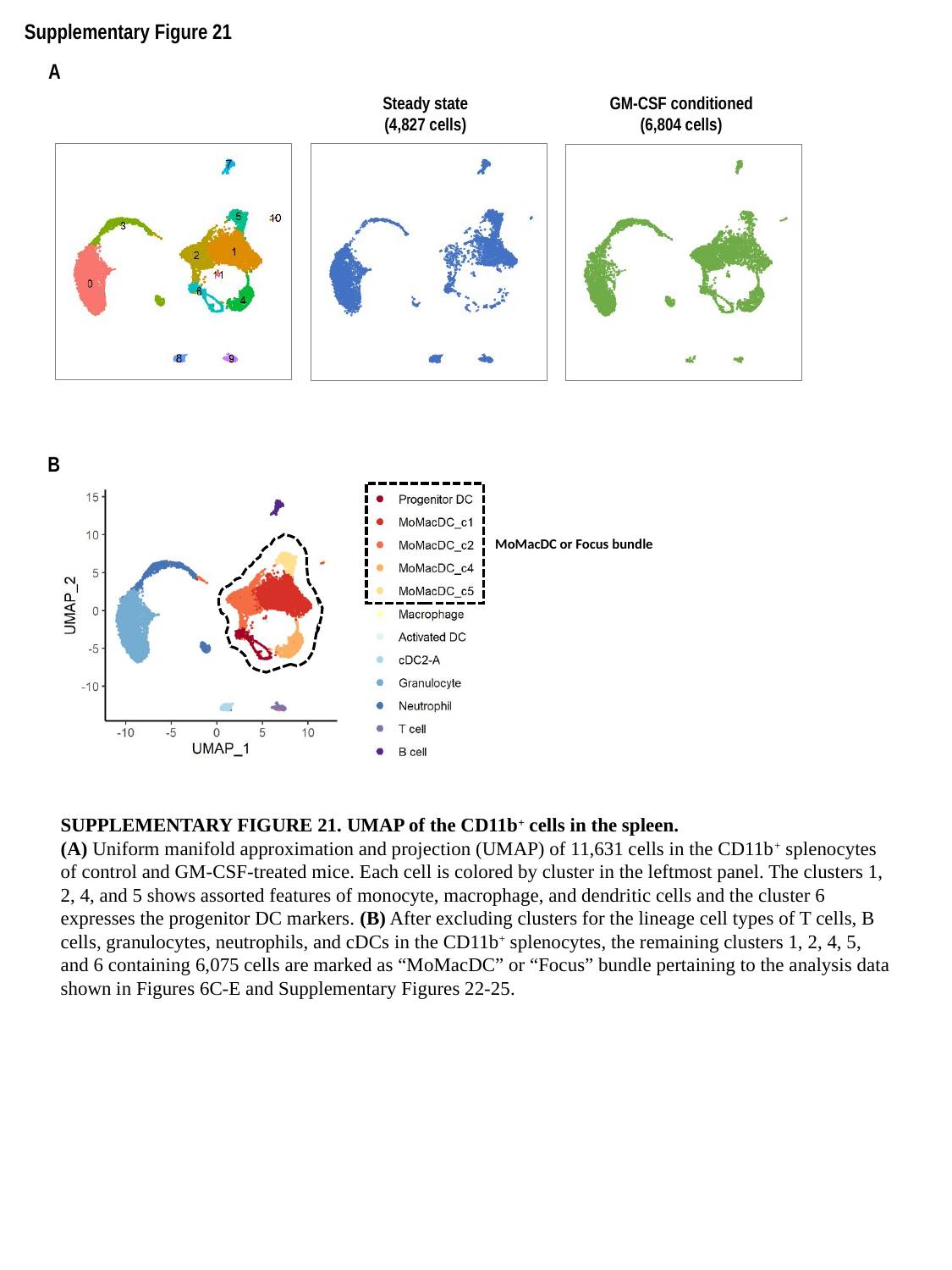

Supplementary Figure 21
A
Steady state
(4,827 cells)
GM-CSF conditioned
(6,804 cells)
B
MoMacDC or Focus bundle
SUPPLEMENTARY FIGURE 21. UMAP of the CD11b+ cells in the spleen.
(A) Uniform manifold approximation and projection (UMAP) of 11,631 cells in the CD11b+ splenocytes of control and GM-CSF-treated mice. Each cell is colored by cluster in the leftmost panel. The clusters 1, 2, 4, and 5 shows assorted features of monocyte, macrophage, and dendritic cells and the cluster 6 expresses the progenitor DC markers. (B) After excluding clusters for the lineage cell types of T cells, B cells, granulocytes, neutrophils, and cDCs in the CD11b+ splenocytes, the remaining clusters 1, 2, 4, 5, and 6 containing 6,075 cells are marked as “MoMacDC” or “Focus” bundle pertaining to the analysis data shown in Figures 6C-E and Supplementary Figures 22-25.

## Slide 24
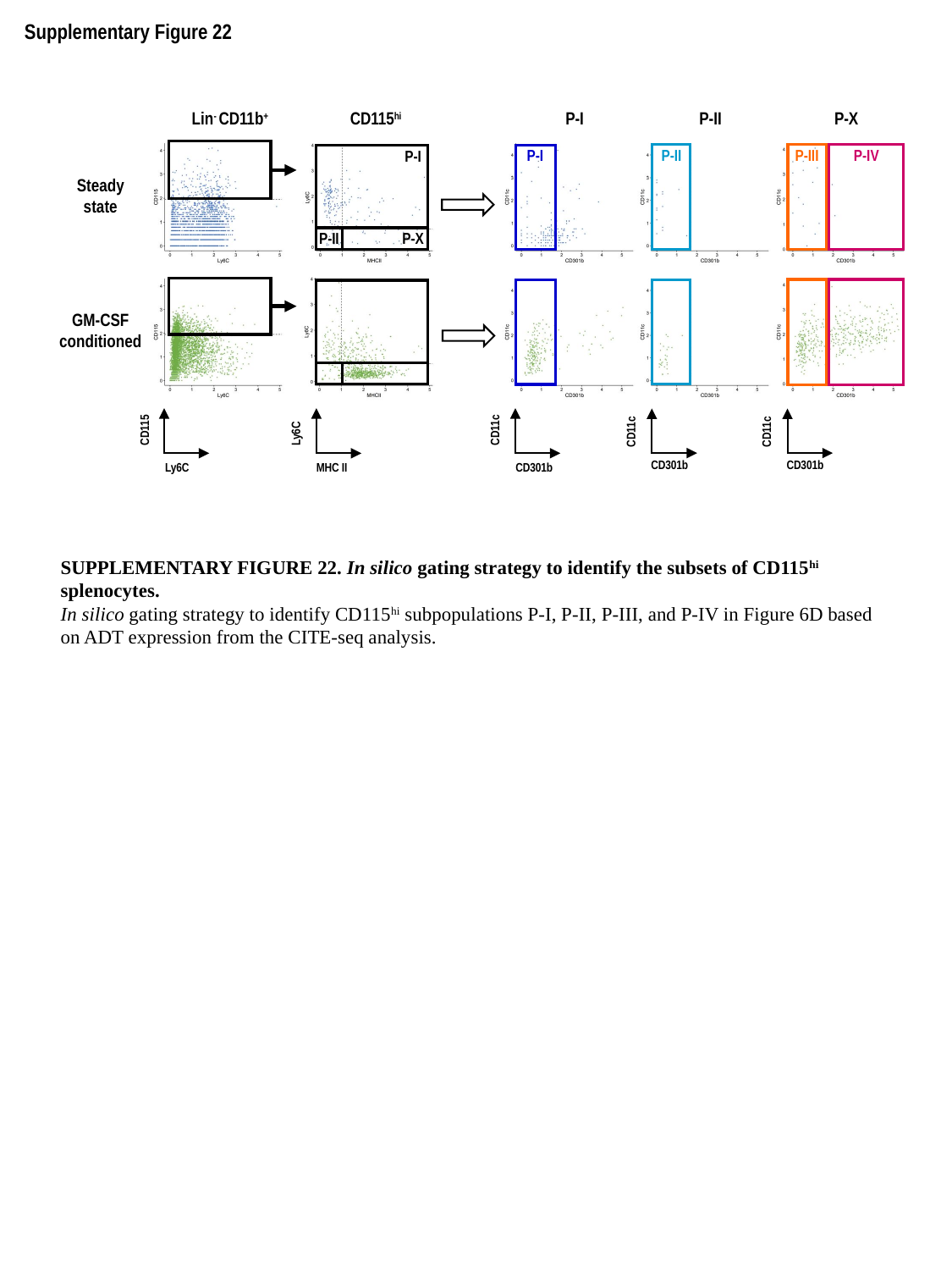

Supplementary Figure 22
Lin- CD11b+
CD115hi
P-I
P-II
P-X
P-I
P-II
P-III
P-IV
P-I
P-II
P-X
Steady
state
GM-CSF
conditioned
CD115
CD11c
CD11c
CD11c
Ly6C
CD301b
CD301b
Ly6C
MHC II
CD301b
SUPPLEMENTARY FIGURE 22. In silico gating strategy to identify the subsets of CD115hi splenocytes.
In silico gating strategy to identify CD115hi subpopulations P-I, P-II, P-III, and P-IV in Figure 6D based on ADT expression from the CITE-seq analysis.

## Slide 25
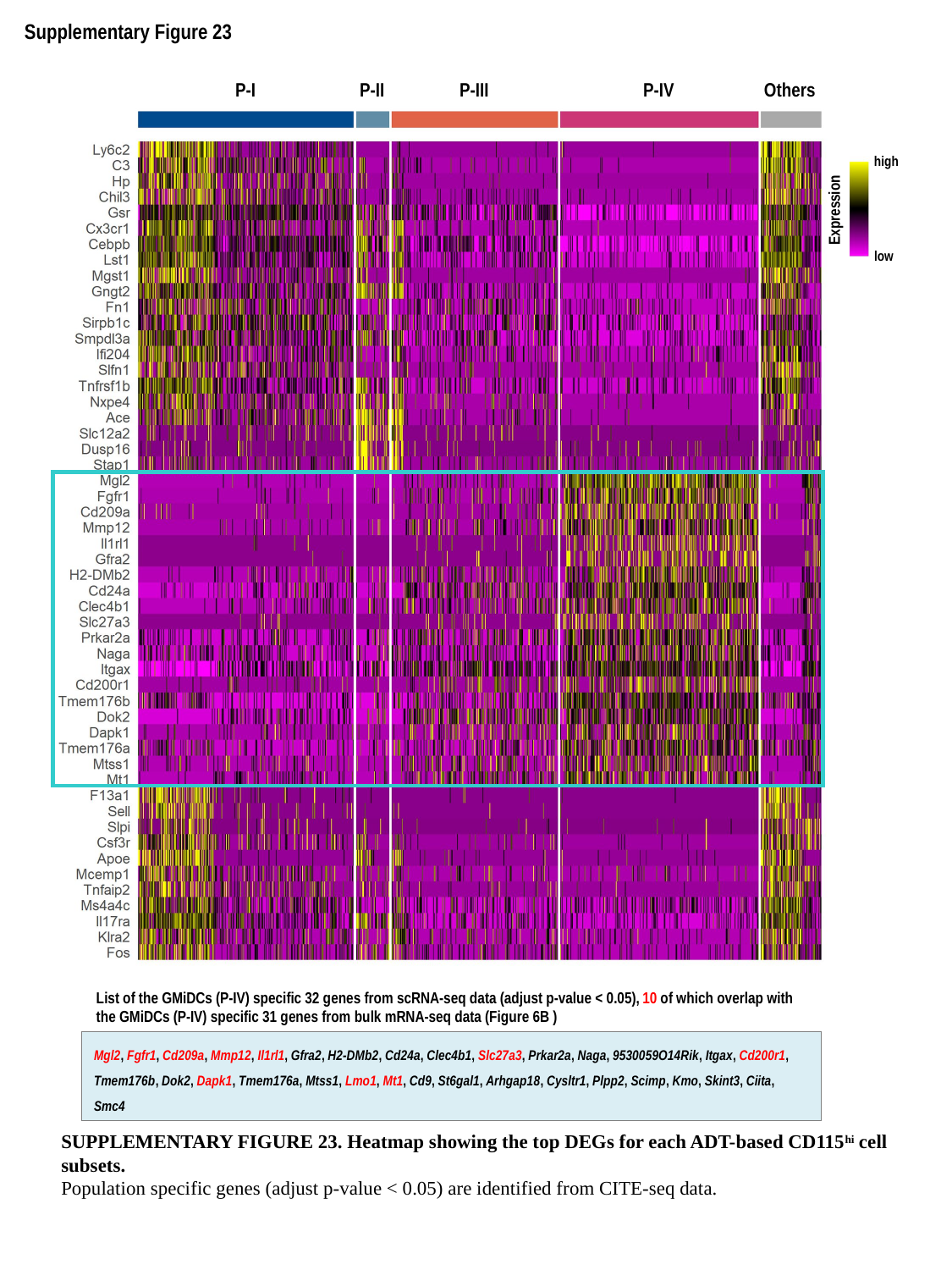

Supplementary Figure 23
P-I
P-II
P-III
P-IV
Others
high
Expression
low
List of the GMiDCs (P-IV) specific 32 genes from scRNA-seq data (adjust p-value < 0.05), 10 of which overlap with the GMiDCs (P-IV) specific 31 genes from bulk mRNA-seq data (Figure 6B )
Mgl2, Fgfr1, Cd209a, Mmp12, Il1rl1, Gfra2, H2-DMb2, Cd24a, Clec4b1, Slc27a3, Prkar2a, Naga, 9530059O14Rik, Itgax, Cd200r1, Tmem176b, Dok2, Dapk1, Tmem176a, Mtss1, Lmo1, Mt1, Cd9, St6gal1, Arhgap18, Cysltr1, Plpp2, Scimp, Kmo, Skint3, Ciita, Smc4
SUPPLEMENTARY FIGURE 23. Heatmap showing the top DEGs for each ADT-based CD115hi cell subsets.
Population specific genes (adjust p-value < 0.05) are identified from CITE-seq data.

## Slide 26
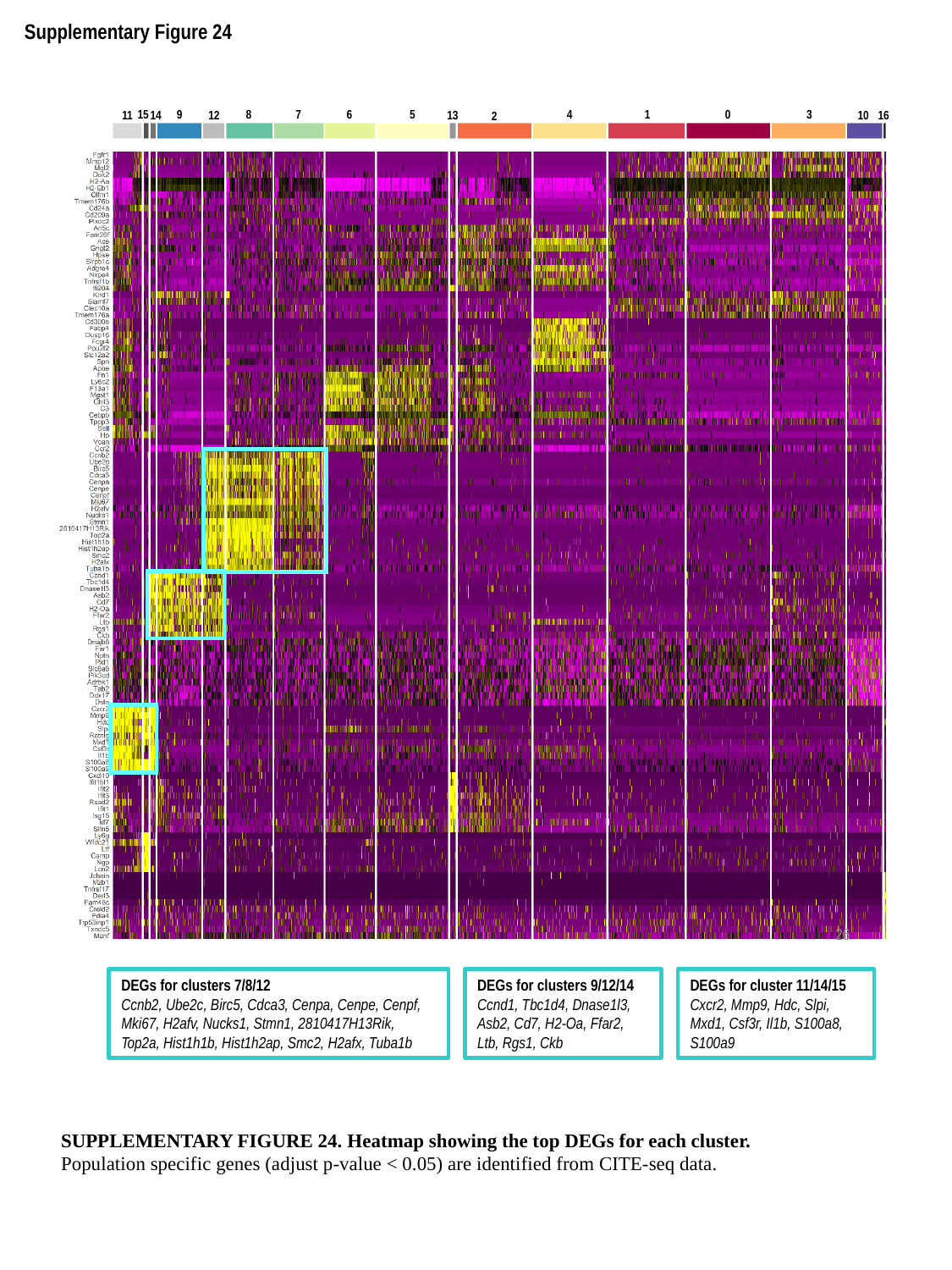

Supplementary Figure 24
15
7
5
9
8
6
4
1
0
3
12
10
11
14
13
16
2
26
DEGs for clusters 7/8/12
Ccnb2, Ube2c, Birc5, Cdca3, Cenpa, Cenpe, Cenpf, Mki67, H2afv, Nucks1, Stmn1, 2810417H13Rik, Top2a, Hist1h1b, Hist1h2ap, Smc2, H2afx, Tuba1b
DEGs for clusters 9/12/14
Ccnd1, Tbc1d4, Dnase1l3, Asb2, Cd7, H2-Oa, Ffar2, Ltb, Rgs1, Ckb
DEGs for cluster 11/14/15
Cxcr2, Mmp9, Hdc, Slpi, Mxd1, Csf3r, Il1b, S100a8, S100a9
SUPPLEMENTARY FIGURE 24. Heatmap showing the top DEGs for each cluster.
Population specific genes (adjust p-value < 0.05) are identified from CITE-seq data.

## Slide 27
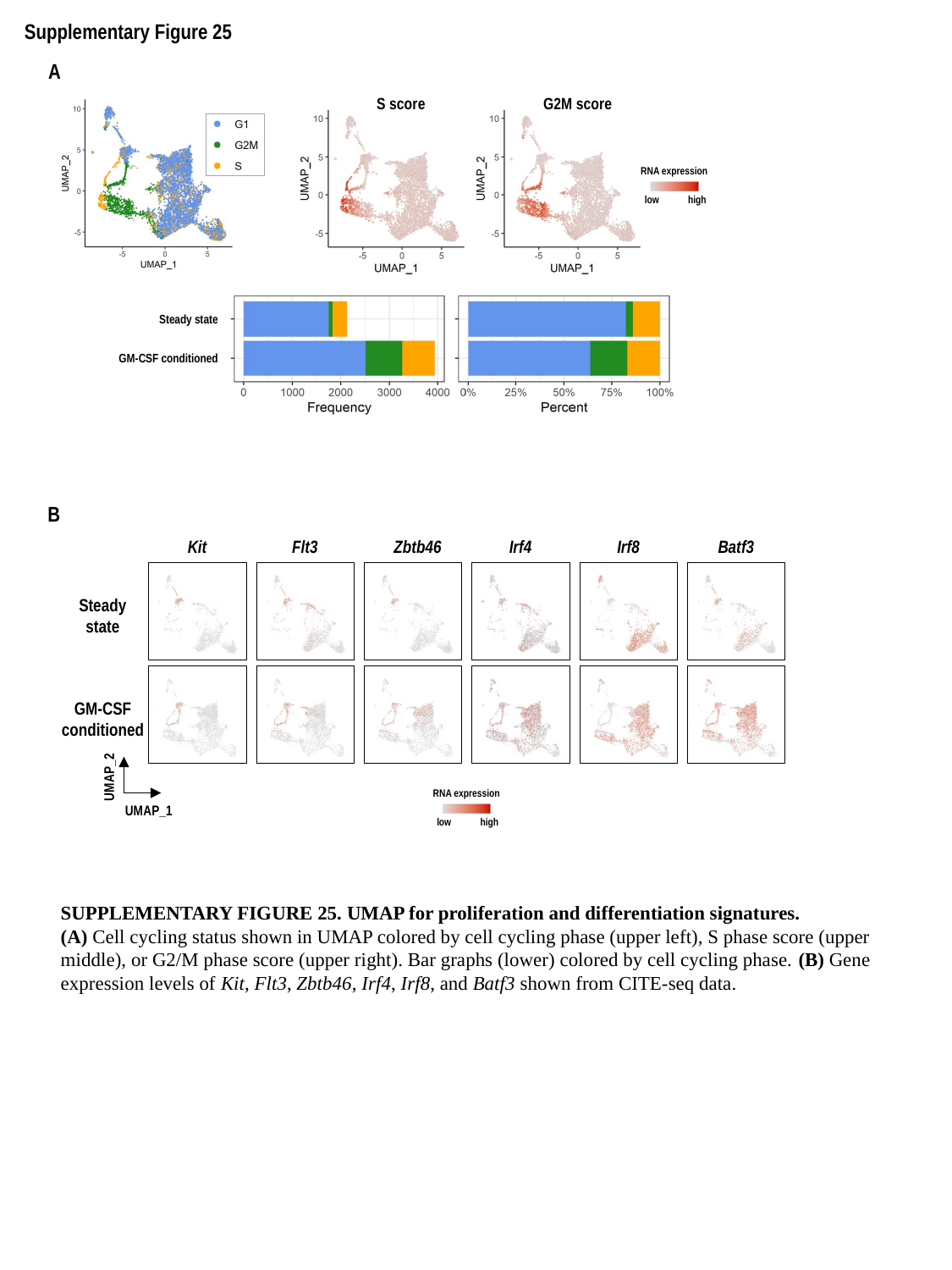

Supplementary Figure 25
A
S score
G2M score
RNA expression
low
high
Steady state
GM-CSF conditioned
B
Kit
Flt3
Zbtb46
Irf4
Irf8
Batf3
Steady
state
GM-CSF
conditioned
UMAP_2
UMAP_1
RNA expression
low
high
SUPPLEMENTARY FIGURE 25. UMAP for proliferation and differentiation signatures.
(A) Cell cycling status shown in UMAP colored by cell cycling phase (upper left), S phase score (upper middle), or G2/M phase score (upper right). Bar graphs (lower) colored by cell cycling phase. (B) Gene expression levels of Kit, Flt3, Zbtb46, Irf4, Irf8, and Batf3 shown from CITE-seq data.

## Slide 28
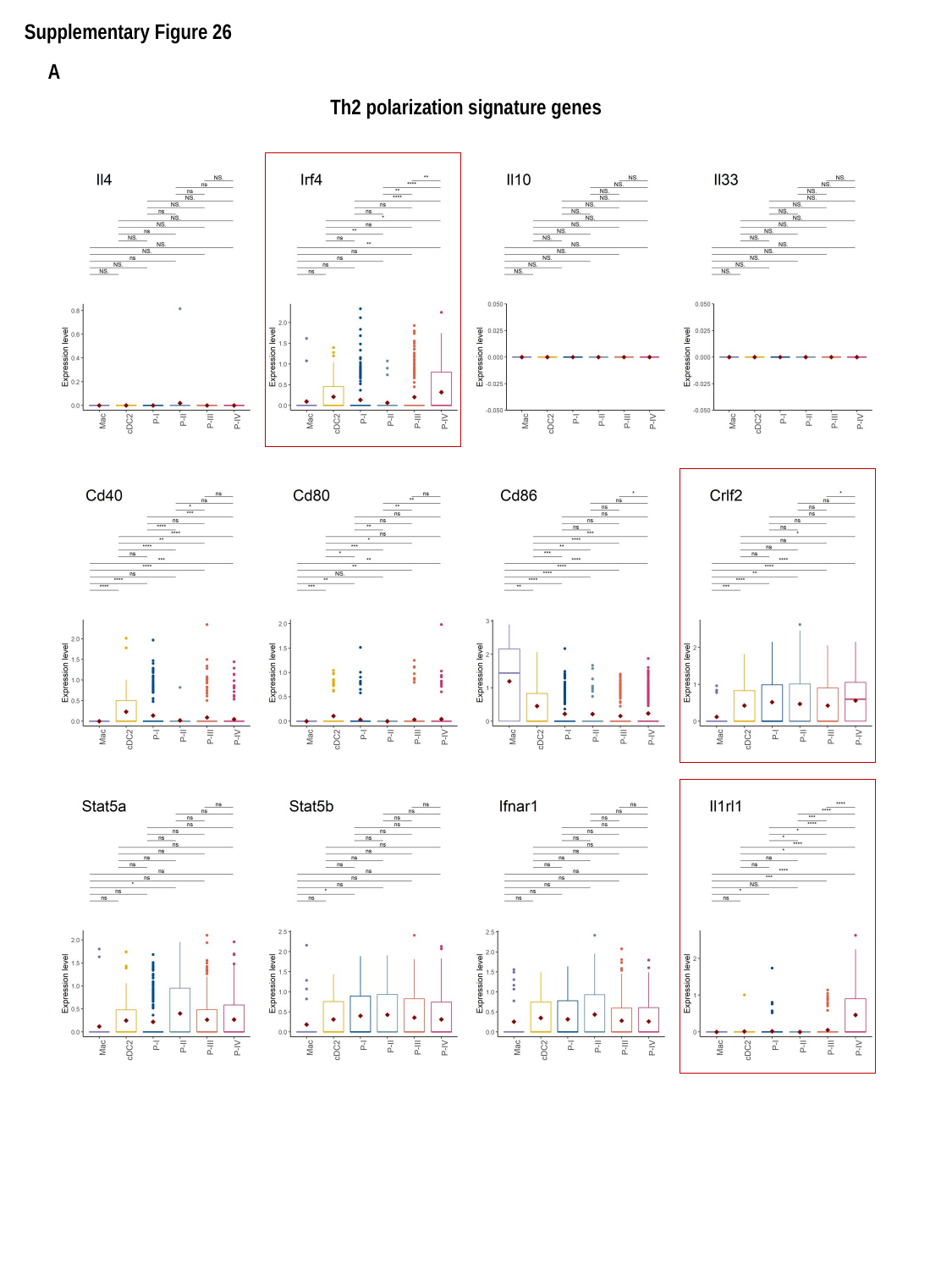

Supplementary Figure 26
A
Th2 polarization signature genes

## Slide 29
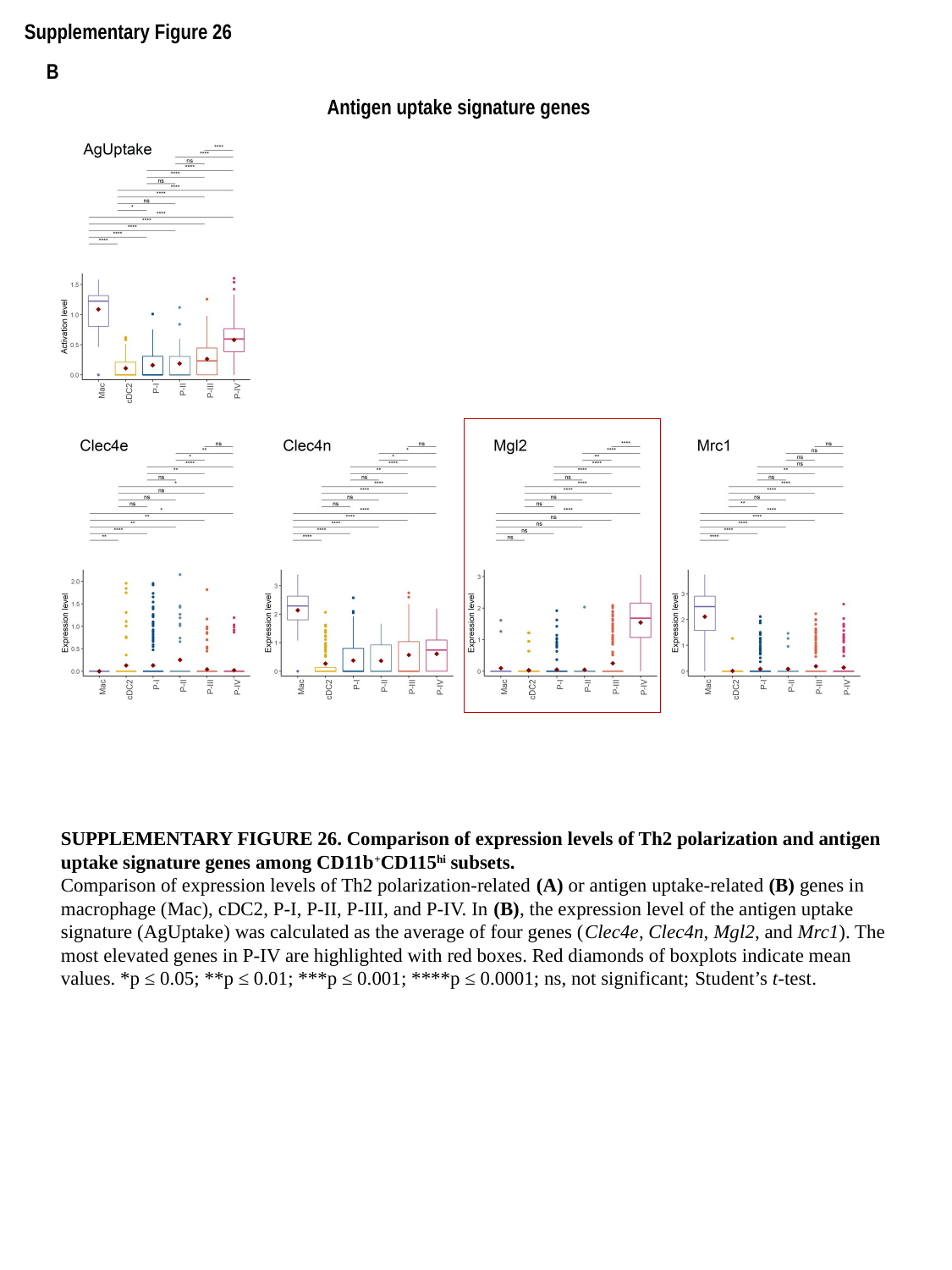

Supplementary Figure 26
B
Antigen uptake signature genes
SUPPLEMENTARY FIGURE 26. Comparison of expression levels of Th2 polarization and antigen uptake signature genes among CD11b+CD115hi subsets.
Comparison of expression levels of Th2 polarization-related (A) or antigen uptake-related (B) genes in macrophage (Mac), cDC2, P-I, P-II, P-III, and P-IV. In (B), the expression level of the antigen uptake signature (AgUptake) was calculated as the average of four genes (Clec4e, Clec4n, Mgl2, and Mrc1). The most elevated genes in P-IV are highlighted with red boxes. Red diamonds of boxplots indicate mean values. *p ≤ 0.05; **p ≤ 0.01; ***p ≤ 0.001; ****p ≤ 0.0001; ns, not significant; Student’s t-test.

## Slide 30
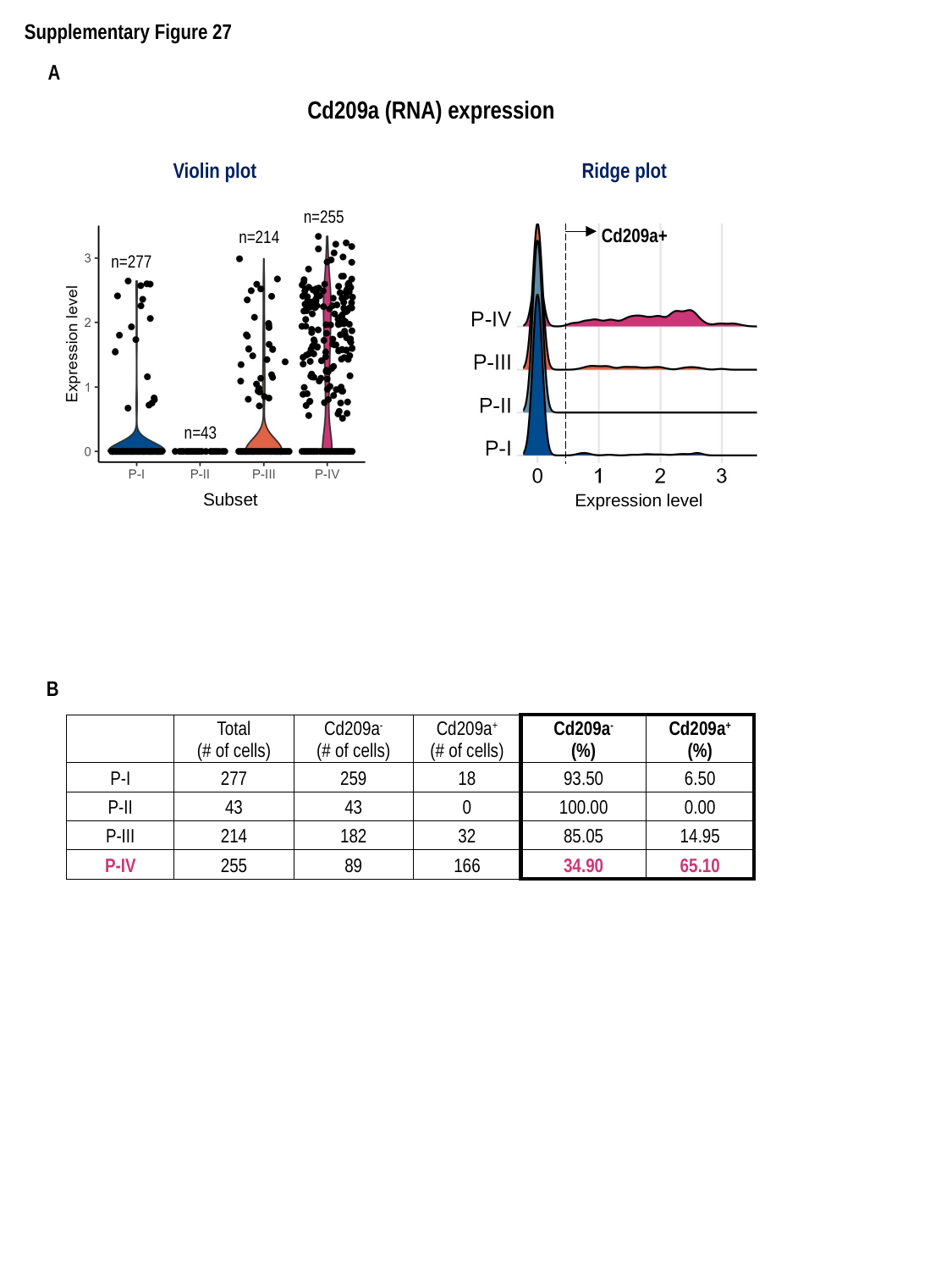

Supplementary Figure 27
A
Cd209a (RNA) expression
Violin plot
Ridge plot
n=255
n=214
n=277
n=43
Subset
Cd209a+
Expression level
B
| | Total (# of cells) | Cd209a- (# of cells) | Cd209a+ (# of cells) | Cd209a- (%) | Cd209a+ (%) |
| --- | --- | --- | --- | --- | --- |
| P-I | 277 | 259 | 18 | 93.50 | 6.50 |
| P-II | 43 | 43 | 0 | 100.00 | 0.00 |
| P-III | 214 | 182 | 32 | 85.05 | 14.95 |
| P-IV | 255 | 89 | 166 | 34.90 | 65.10 |

## Slide 31
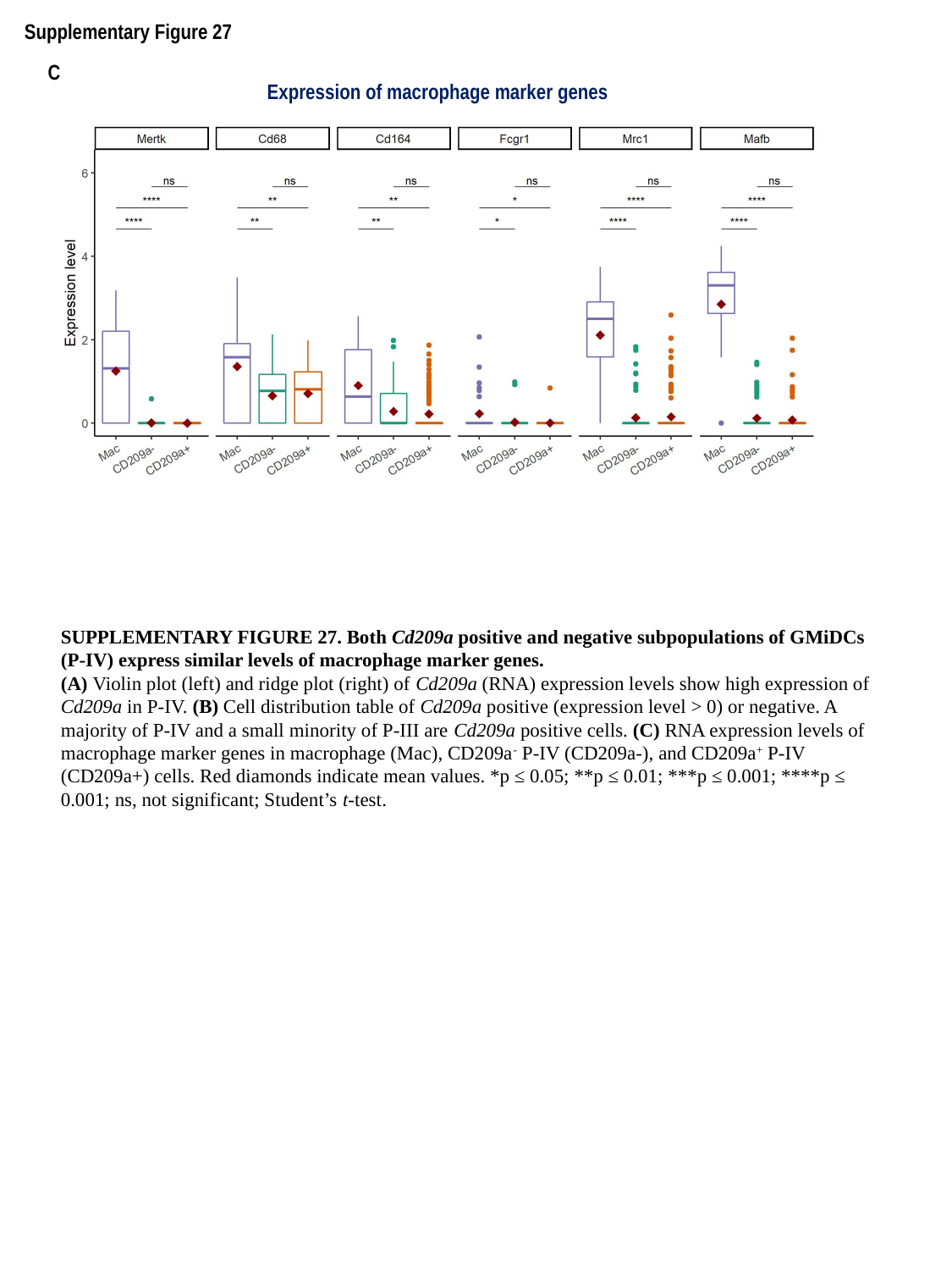

Supplementary Figure 27
C
Expression of macrophage marker genes
SUPPLEMENTARY FIGURE 27. Both Cd209a positive and negative subpopulations of GMiDCs (P-IV) express similar levels of macrophage marker genes.
(A) Violin plot (left) and ridge plot (right) of Cd209a (RNA) expression levels show high expression of Cd209a in P-IV. (B) Cell distribution table of Cd209a positive (expression level > 0) or negative. A majority of P-IV and a small minority of P-III are Cd209a positive cells. (C) RNA expression levels of macrophage marker genes in macrophage (Mac), CD209a- P-IV (CD209a-), and CD209a+ P-IV (CD209a+) cells. Red diamonds indicate mean values. *p ≤ 0.05; **p ≤ 0.01; ***p ≤ 0.001; ****p ≤ 0.001; ns, not significant; Student’s t-test.

## Slide 32
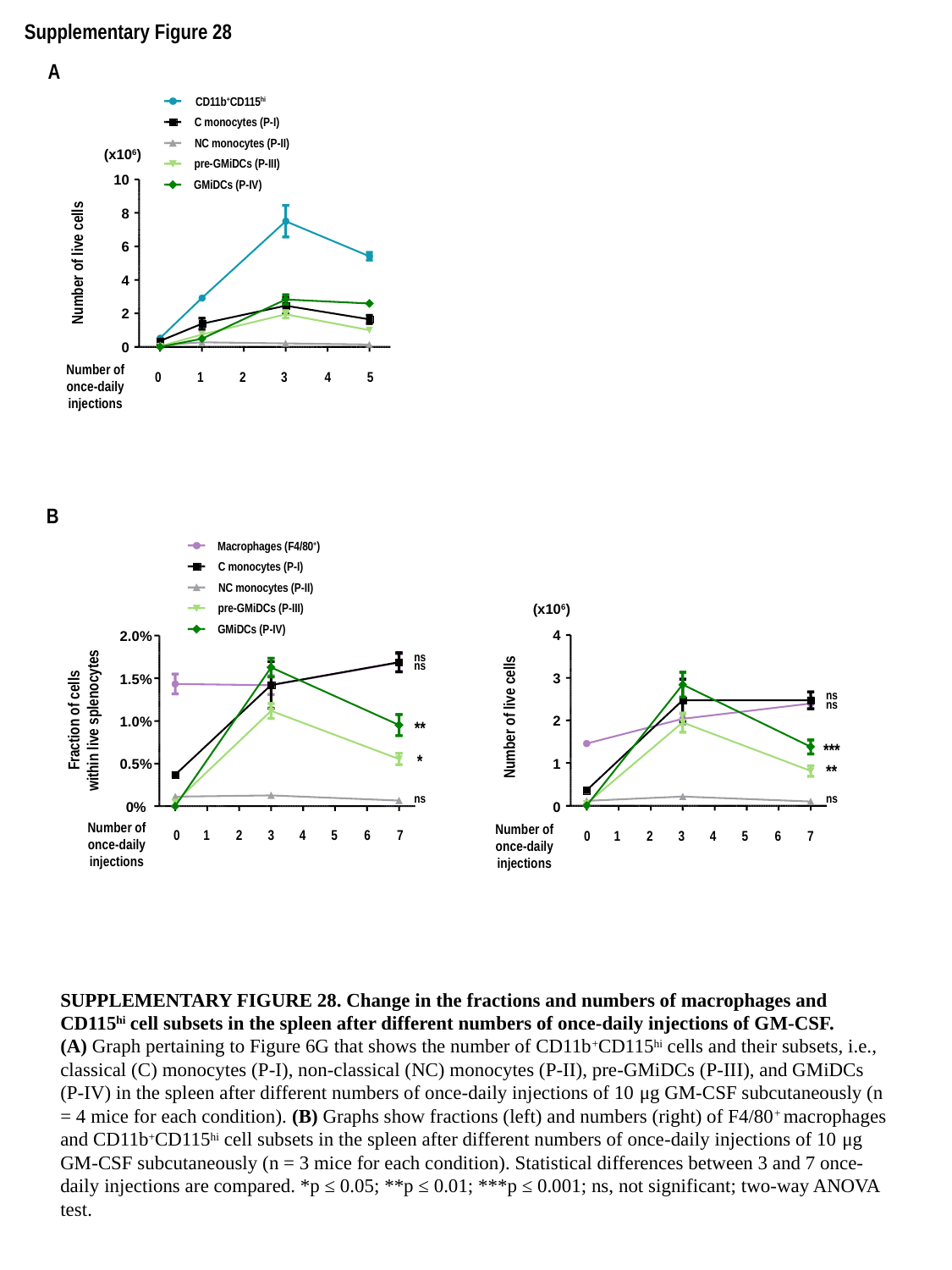

Supplementary Figure 28
A
CD11b+CD115hi
C monocytes (P-I)
NC monocytes (P-II)
pre-GMiDCs (P-III)
GMiDCs (P-IV)
(x106)
10
Number of live cells
8
6
4
2
0
Number of
once-daily
injections
0
1
2
3
4
5
B
Macrophages (F4/80+)
C monocytes (P-I)
NC monocytes (P-II)
pre-GMiDCs (P-III)
GMiDCs (P-IV)
(x106)
4
3
2
1
0
Number of live cells
Number of
once-daily
injections
0
1
2
3
4
5
6
7
2.0%
1.5%
1.0%
0.5%
0%
Fraction of cells
within live splenocytes
Number of
once-daily
injections
0
1
2
3
4
5
6
7
ns
ns
**
*
ns
ns
ns
***
**
ns
SUPPLEMENTARY FIGURE 28. Change in the fractions and numbers of macrophages and CD115hi cell subsets in the spleen after different numbers of once-daily injections of GM-CSF.
(A) Graph pertaining to Figure 6G that shows the number of CD11b+CD115hi cells and their subsets, i.e., classical (C) monocytes (P-I), non-classical (NC) monocytes (P-II), pre-GMiDCs (P-III), and GMiDCs (P-IV) in the spleen after different numbers of once-daily injections of 10 μg GM-CSF subcutaneously (n = 4 mice for each condition). (B) Graphs show fractions (left) and numbers (right) of F4/80+ macrophages and CD11b+CD115hi cell subsets in the spleen after different numbers of once-daily injections of 10 μg GM-CSF subcutaneously (n = 3 mice for each condition). Statistical differences between 3 and 7 once-daily injections are compared. *p ≤ 0.05; **p ≤ 0.01; ***p ≤ 0.001; ns, not significant; two-way ANOVA test.

## Slide 33
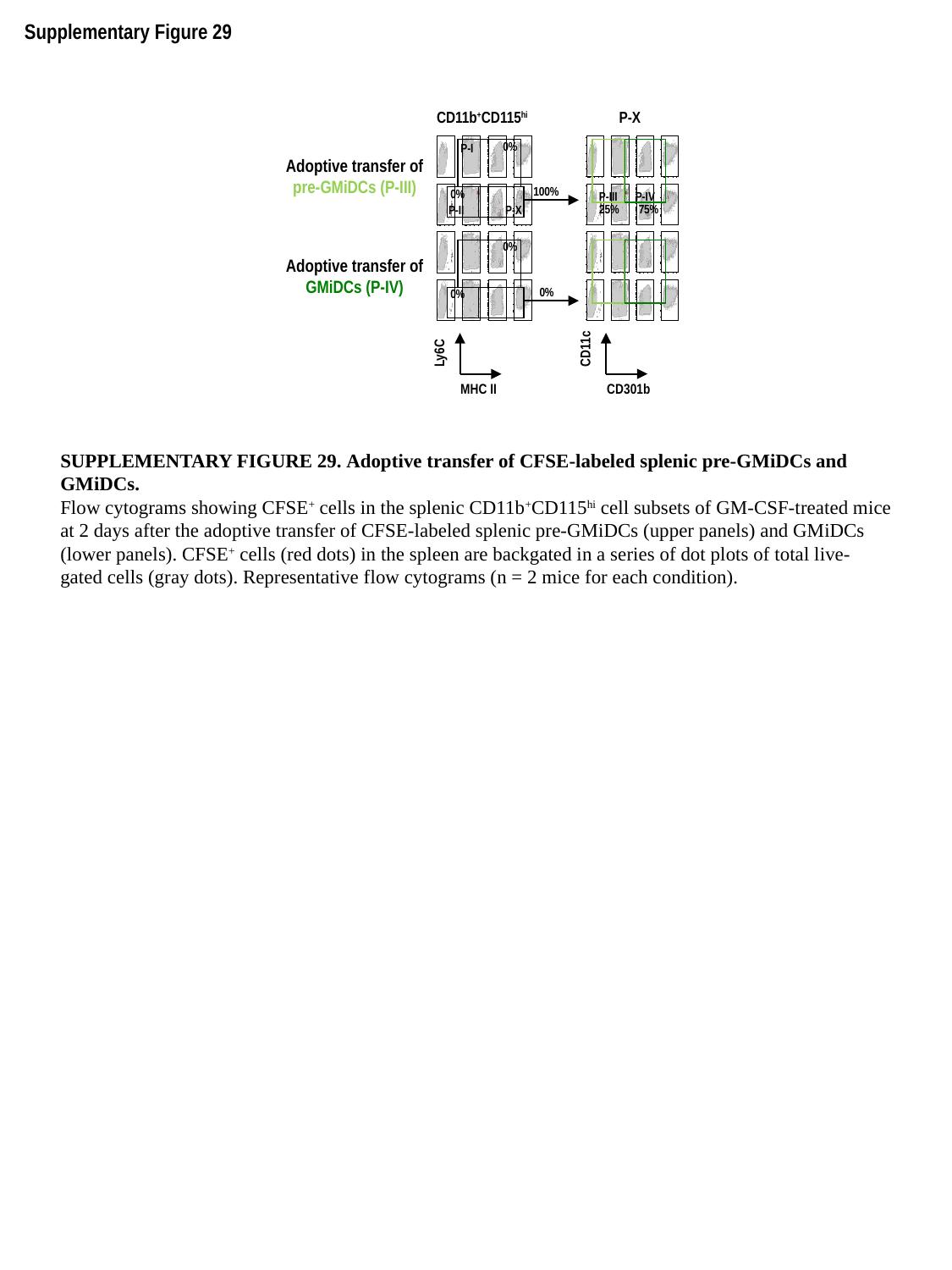

Supplementary Figure 29
CD11b+CD115hi
P-X
P-I
Adoptive transfer of
pre-GMiDCs (P-III)
P-III
P-IV
P-X
P-II
Adoptive transfer of
GMiDCs (P-IV)
CD11c
CD301b
Ly6C
MHC II
0%
100%
0%
25%
75%
0%
0%
0%
SUPPLEMENTARY FIGURE 29. Adoptive transfer of CFSE-labeled splenic pre-GMiDCs and GMiDCs.
Flow cytograms showing CFSE+ cells in the splenic CD11b+CD115hi cell subsets of GM-CSF-treated mice at 2 days after the adoptive transfer of CFSE-labeled splenic pre-GMiDCs (upper panels) and GMiDCs (lower panels). CFSE+ cells (red dots) in the spleen are backgated in a series of dot plots of total live-gated cells (gray dots). Representative flow cytograms (n = 2 mice for each condition).

## Slide 34
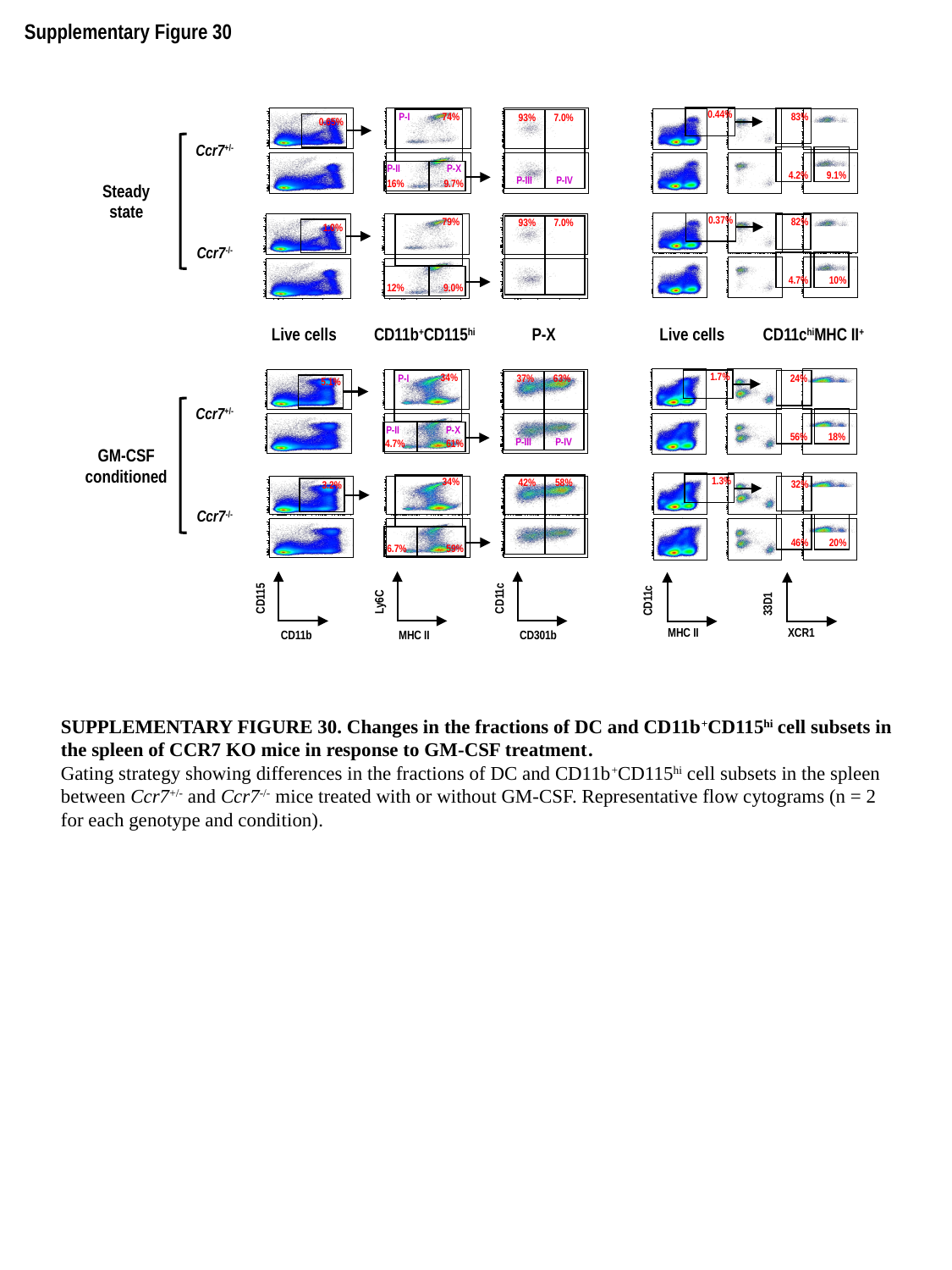

Supplementary Figure 30
0.44%
83%
74%
P-I
93%
7.0%
0.65%
Ccr7+/-
P-II
P-X
4.2%
9.1%
P-III
P-IV
16%
9.7%
Steady
state
0.37%
82%
79%
93%
7.0%
1.0%
Ccr7-/-
4.7%
10%
12%
9.0%
Live cells
CD11b+CD115hi
P-X
Live cells
CD11chiMHC II+
1.7%
34%
P-I
24%
37%
63%
5.1%
Ccr7+/-
P-II
P-X
56%
18%
P-III
P-IV
4.7%
61%
GM-CSF
conditioned
1.3%
34%
42%
58%
32%
3.2%
Ccr7-/-
46%
20%
6.7%
59%
CD115
CD11b
Ly6C
MHC II
CD11c
CD301b
CD11c
MHC II
33D1
XCR1
SUPPLEMENTARY FIGURE 30. Changes in the fractions of DC and CD11b+CD115hi cell subsets in the spleen of CCR7 KO mice in response to GM-CSF treatment.
Gating strategy showing differences in the fractions of DC and CD11b+CD115hi cell subsets in the spleen between Ccr7+/- and Ccr7-/- mice treated with or without GM-CSF. Representative flow cytograms (n = 2 for each genotype and condition).

## Slide 35
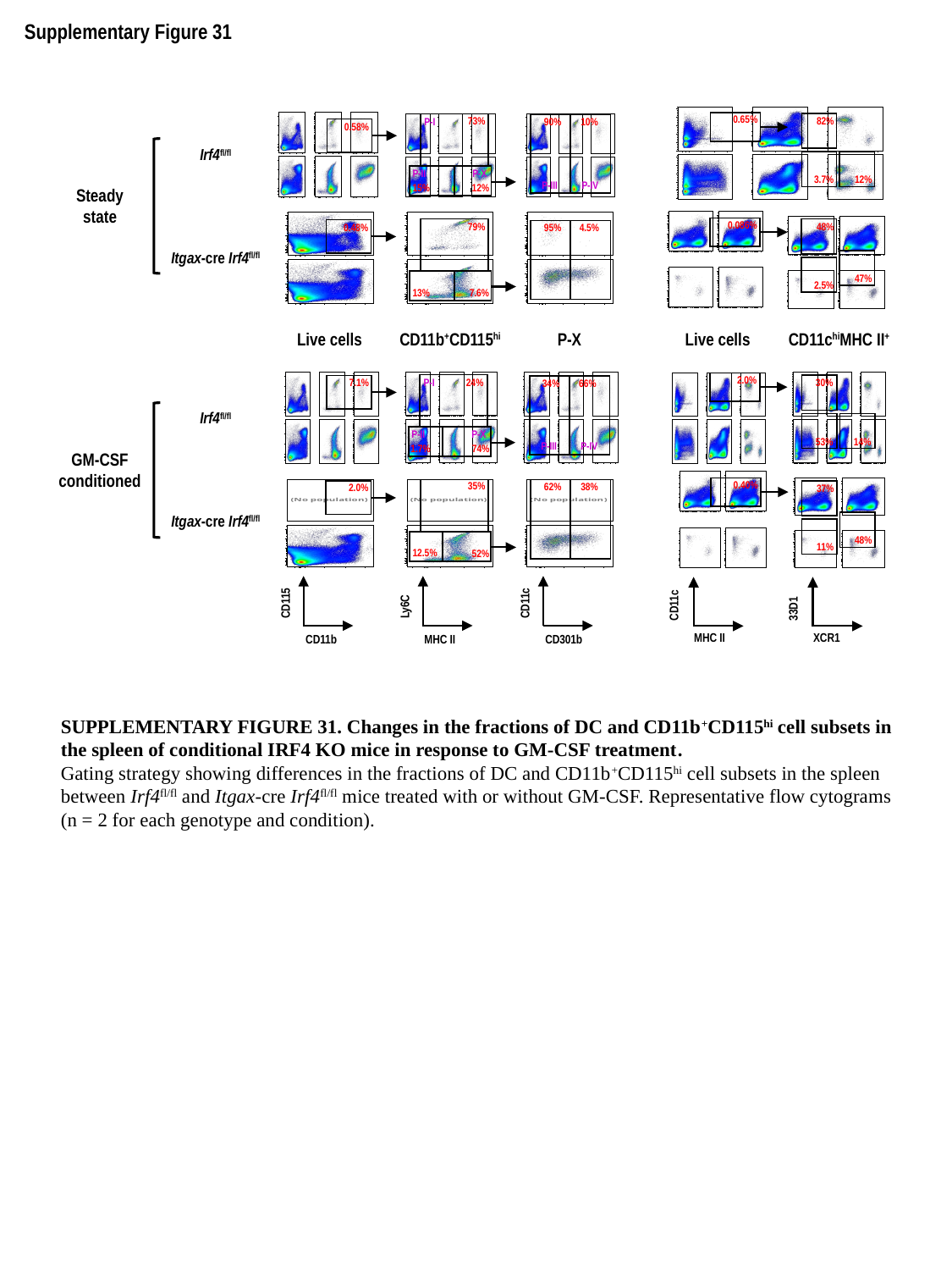

Supplementary Figure 31
0.65%
82%
73%
P-I
90%
10%
0.58%
Irf4fl/fl
P-II
P-X
3.7%
12%
P-III
P-IV
15%
12%
Steady
state
0.086%
48%
79%
95%
4.5%
0.48%
Itgax-cre Irf4fl/fl
47%
2.5%
13%
7.6%
Live cells
CD11b+CD115hi
P-X
Live cells
CD11chiMHC II+
2.0%
24%
7.1%
P-I
30%
34%
66%
Irf4fl/fl
P-II
P-X
53%
14%
P-III
P-IV
1.7%
74%
GM-CSF
conditioned
0.40%
35%
62%
38%
2.0%
37%
Itgax-cre Irf4fl/fl
48%
11%
12.5%
52%
CD115
CD11b
Ly6C
MHC II
CD11c
CD301b
CD11c
MHC II
33D1
XCR1
SUPPLEMENTARY FIGURE 31. Changes in the fractions of DC and CD11b+CD115hi cell subsets in the spleen of conditional IRF4 KO mice in response to GM-CSF treatment.
Gating strategy showing differences in the fractions of DC and CD11b+CD115hi cell subsets in the spleen between Irf4fl/fl and Itgax-cre Irf4fl/fl mice treated with or without GM-CSF. Representative flow cytograms (n = 2 for each genotype and condition).

## Slide 36
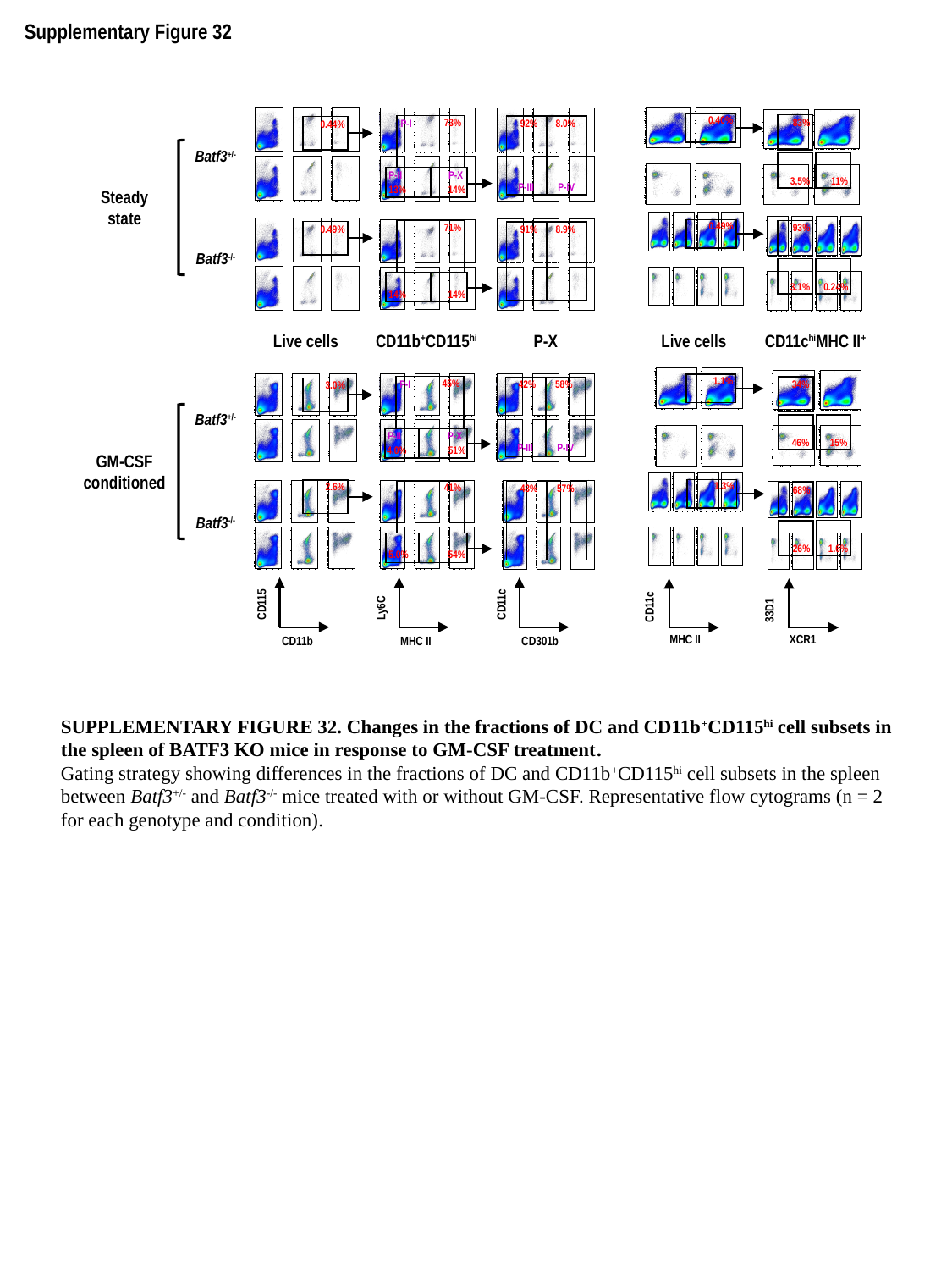

Supplementary Figure 32
0.40%
83%
73%
P-I
92%
8.0%
0.44%
Batf3+/-
P-II
P-X
3.5%
11%
P-III
P-IV
13%
14%
Steady
state
0.49%
93%
71%
91%
8.9%
0.49%
Batf3-/-
3.1%
0.24%
14%
14%
Live cells
CD11b+CD115hi
P-X
Live cells
CD11chiMHC II+
1.1%
45%
P-I
34%
42%
58%
3.0%
Batf3+/-
P-II
P-X
46%
15%
P-III
P-IV
4.0%
51%
GM-CSF
conditioned
1.3%
2.6%
41%
43%
57%
68%
Batf3-/-
1.6%
26%
5.0%
54%
CD115
CD11b
Ly6C
MHC II
CD11c
CD301b
CD11c
MHC II
33D1
XCR1
SUPPLEMENTARY FIGURE 32. Changes in the fractions of DC and CD11b+CD115hi cell subsets in the spleen of BATF3 KO mice in response to GM-CSF treatment.
Gating strategy showing differences in the fractions of DC and CD11b+CD115hi cell subsets in the spleen between Batf3+/- and Batf3-/- mice treated with or without GM-CSF. Representative flow cytograms (n = 2 for each genotype and condition).

## Slide 37
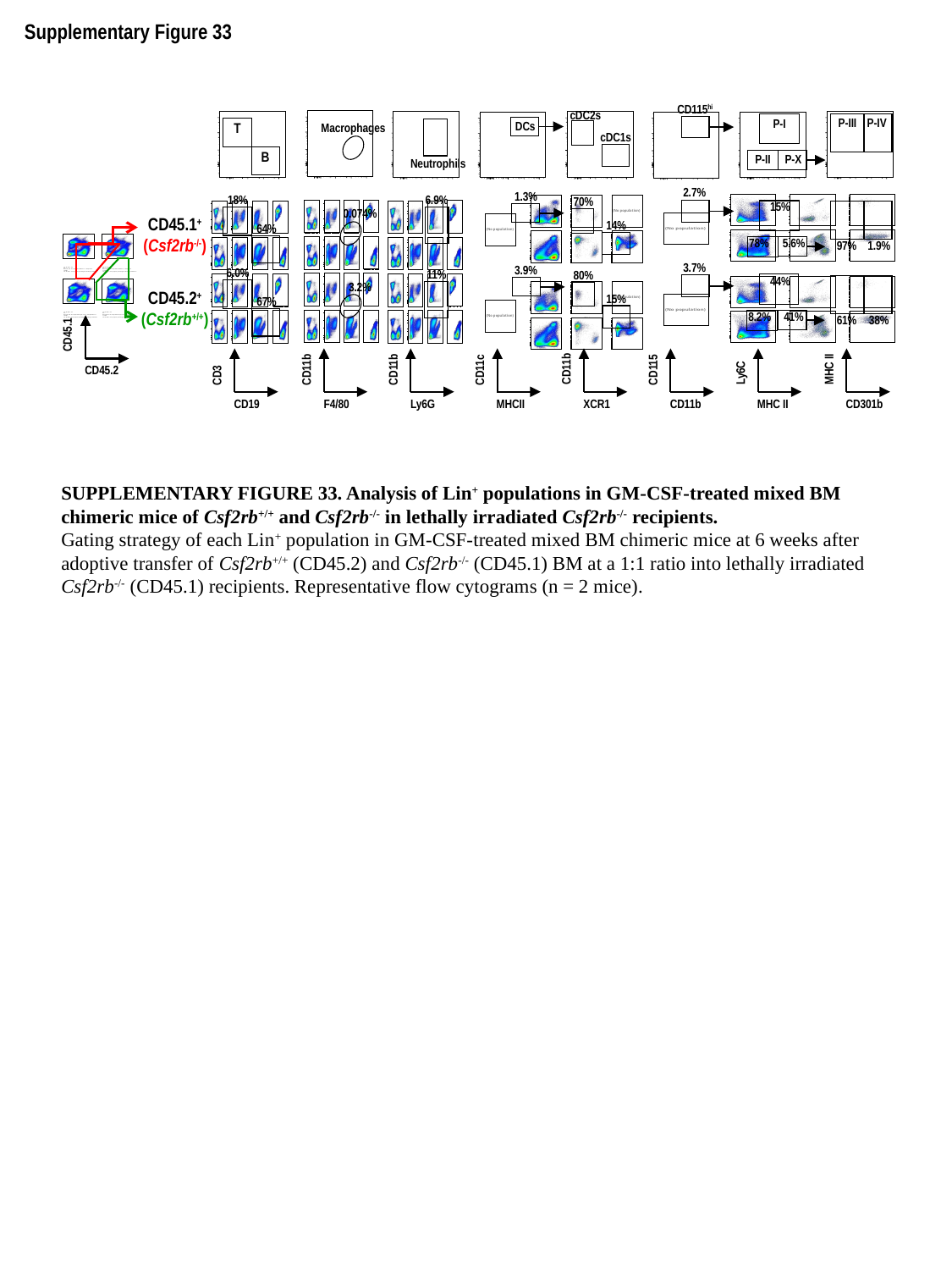

Supplementary Figure 33
CD115hi
cDC2s
P-III
P-IV
P-I
DCs
T
Macrophages
cDC1s
B
P-X
P-II
Neutrophils
CD45.1+
(Csf2rb-/-)
CD45.2+
(Csf2rb+/+)
CD45.1
CD45.2
MHC II
CD301b
CD3
CD19
CD11b
F4/80
CD11b
Ly6G
CD11c
MHCII
CD11b
XCR1
CD115
CD11b
Ly6C
MHC II
2.7%
3.7%
1.3%
3.9%
6.9%
11%
18%
6.0%
70%
80%
15%
44%
0.074%
3.2%
14%
15%
64%
67%
78%
8.2%
5.6%
41%
97%
1.9%
61%
38%
SUPPLEMENTARY FIGURE 33. Analysis of Lin+ populations in GM-CSF-treated mixed BM chimeric mice of Csf2rb+/+ and Csf2rb-/- in lethally irradiated Csf2rb-/- recipients.
Gating strategy of each Lin+ population in GM-CSF-treated mixed BM chimeric mice at 6 weeks after adoptive transfer of Csf2rb+/+ (CD45.2) and Csf2rb-/- (CD45.1) BM at a 1:1 ratio into lethally irradiated Csf2rb-/- (CD45.1) recipients. Representative flow cytograms (n = 2 mice).

## Slide 38
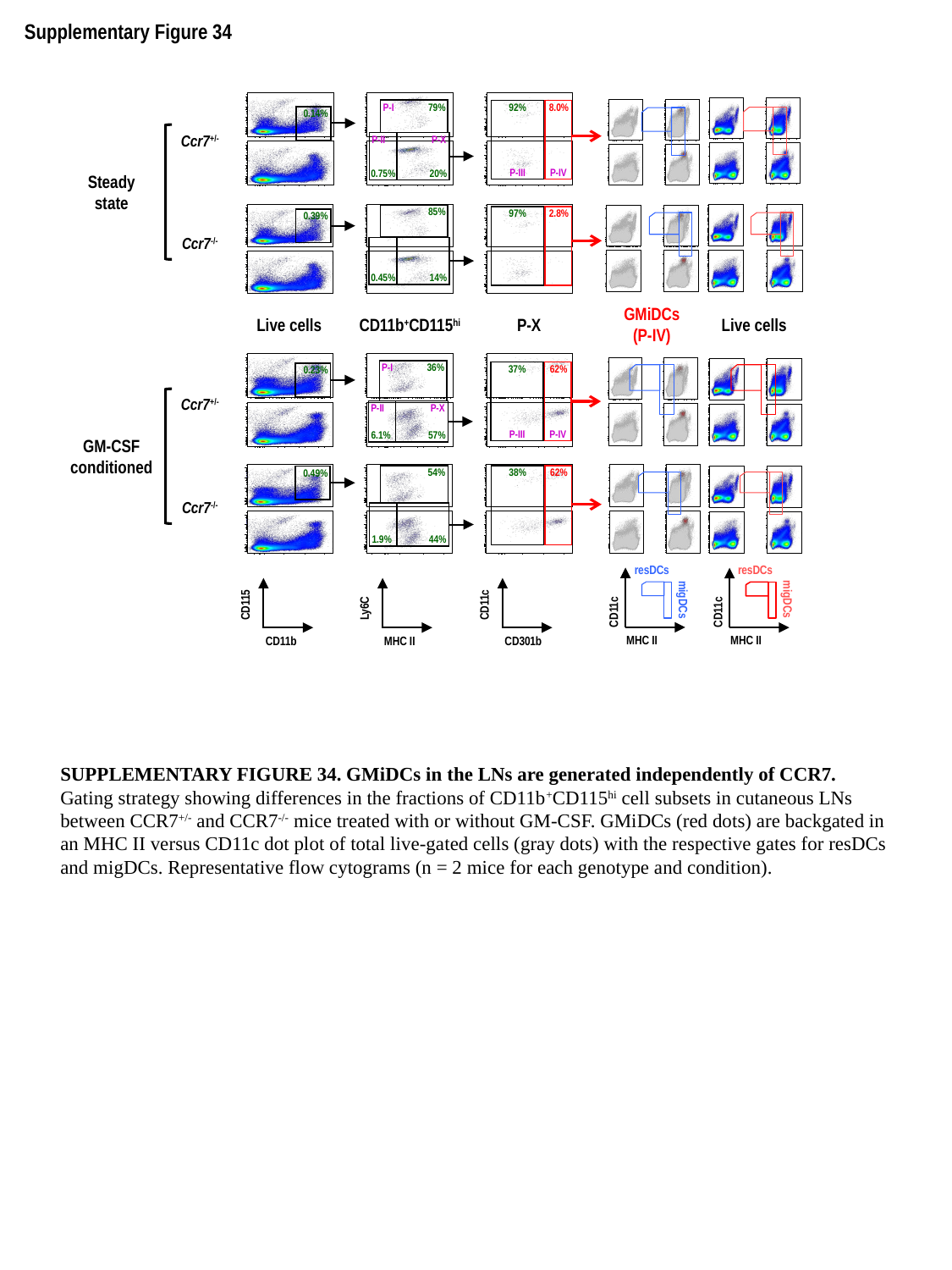

Supplementary Figure 34
79%
P-I
92%
8.0%
0.14%
Ccr7+/-
P-II
P-X
P-III
P-IV
0.75%
20%
Steady
state
85%
97%
2.8%
0.39%
Ccr7-/-
0.45%
14%
GMiDCs
(P-IV)
Live cells
CD11b+CD115hi
P-X
Live cells
36%
P-I
37%
62%
0.23%
Ccr7+/-
P-II
P-X
P-III
P-IV
6.1%
57%
GM-CSF
conditioned
54%
38%
62%
0.49%
Ccr7-/-
1.9%
44%
resDCs
migDCs
CD11c
MHC II
resDCs
migDCs
CD11c
MHC II
CD115
CD11b
Ly6C
MHC II
CD11c
CD301b
SUPPLEMENTARY FIGURE 34. GMiDCs in the LNs are generated independently of CCR7.
Gating strategy showing differences in the fractions of CD11b+CD115hi cell subsets in cutaneous LNs between CCR7+/- and CCR7-/- mice treated with or without GM-CSF. GMiDCs (red dots) are backgated in an MHC II versus CD11c dot plot of total live-gated cells (gray dots) with the respective gates for resDCs and migDCs. Representative flow cytograms (n = 2 mice for each genotype and condition).

## Slide 39
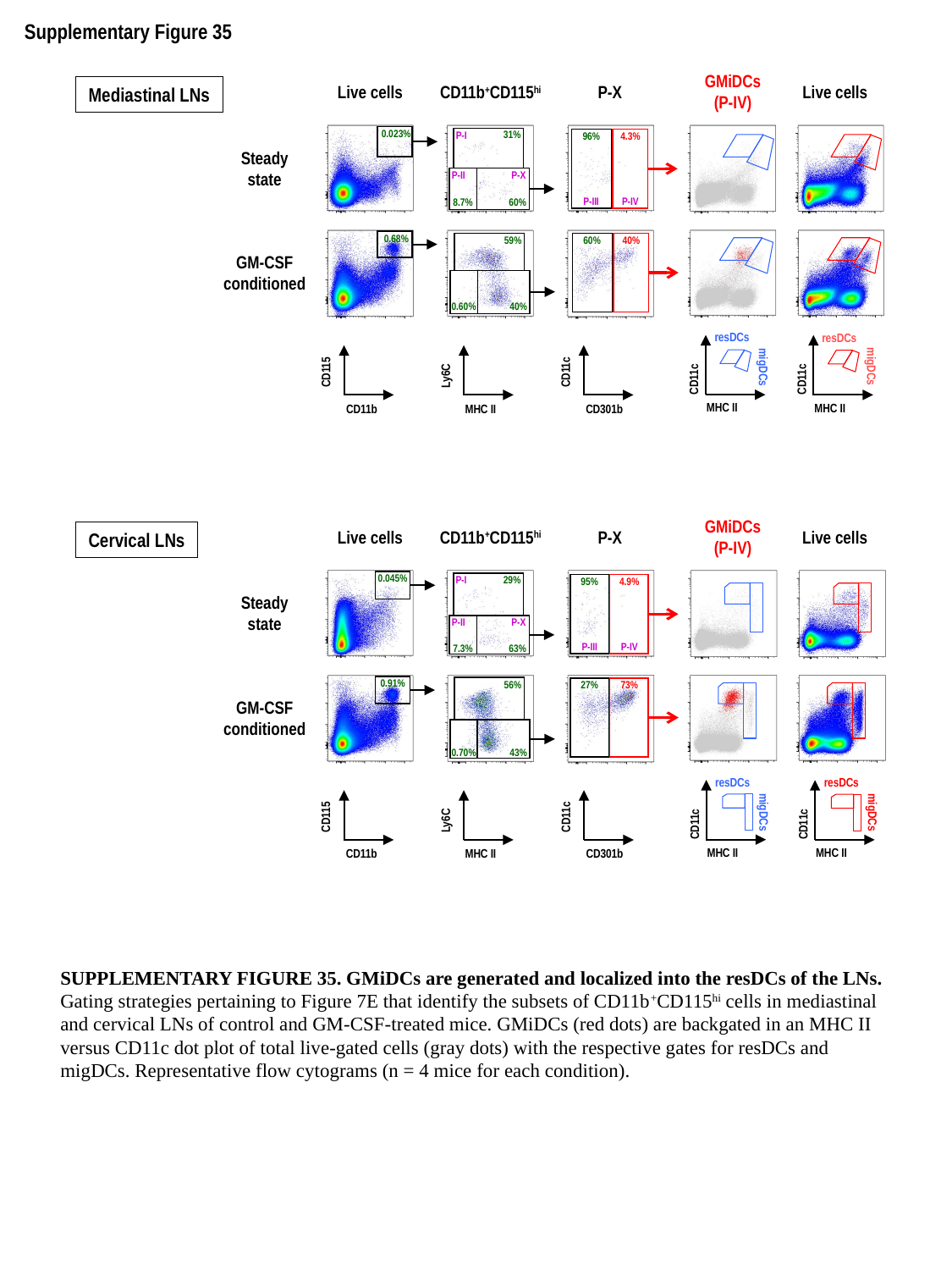

Supplementary Figure 35
GMiDCs
(P-IV)
Live cells
CD11b+CD115hi
P-X
Live cells
0.023%
31%
P-I
96%
4.3%
Steady
state
P-II
P-X
P-III
P-IV
8.7%
60%
0.68%
60%
40%
59%
GM-CSF
conditioned
0.60%
40%
resDCs
migDCs
CD11c
MHC II
resDCs
migDCs
CD11c
MHC II
CD115
CD11b
Ly6C
MHC II
CD11c
CD301b
Mediastinal LNs
GMiDCs
(P-IV)
Live cells
CD11b+CD115hi
P-X
Live cells
0.045%
29%
P-I
95%
4.9%
Steady
state
P-II
P-X
P-III
P-IV
7.3%
63%
0.91%
56%
27%
73%
GM-CSF
conditioned
0.70%
43%
resDCs
migDCs
CD11c
MHC II
resDCs
migDCs
CD11c
MHC II
CD115
CD11b
Ly6C
MHC II
CD11c
CD301b
Cervical LNs
SUPPLEMENTARY FIGURE 35. GMiDCs are generated and localized into the resDCs of the LNs.
Gating strategies pertaining to Figure 7E that identify the subsets of CD11b+CD115hi cells in mediastinal and cervical LNs of control and GM-CSF-treated mice. GMiDCs (red dots) are backgated in an MHC II versus CD11c dot plot of total live-gated cells (gray dots) with the respective gates for resDCs and migDCs. Representative flow cytograms (n = 4 mice for each condition).

## Slide 40
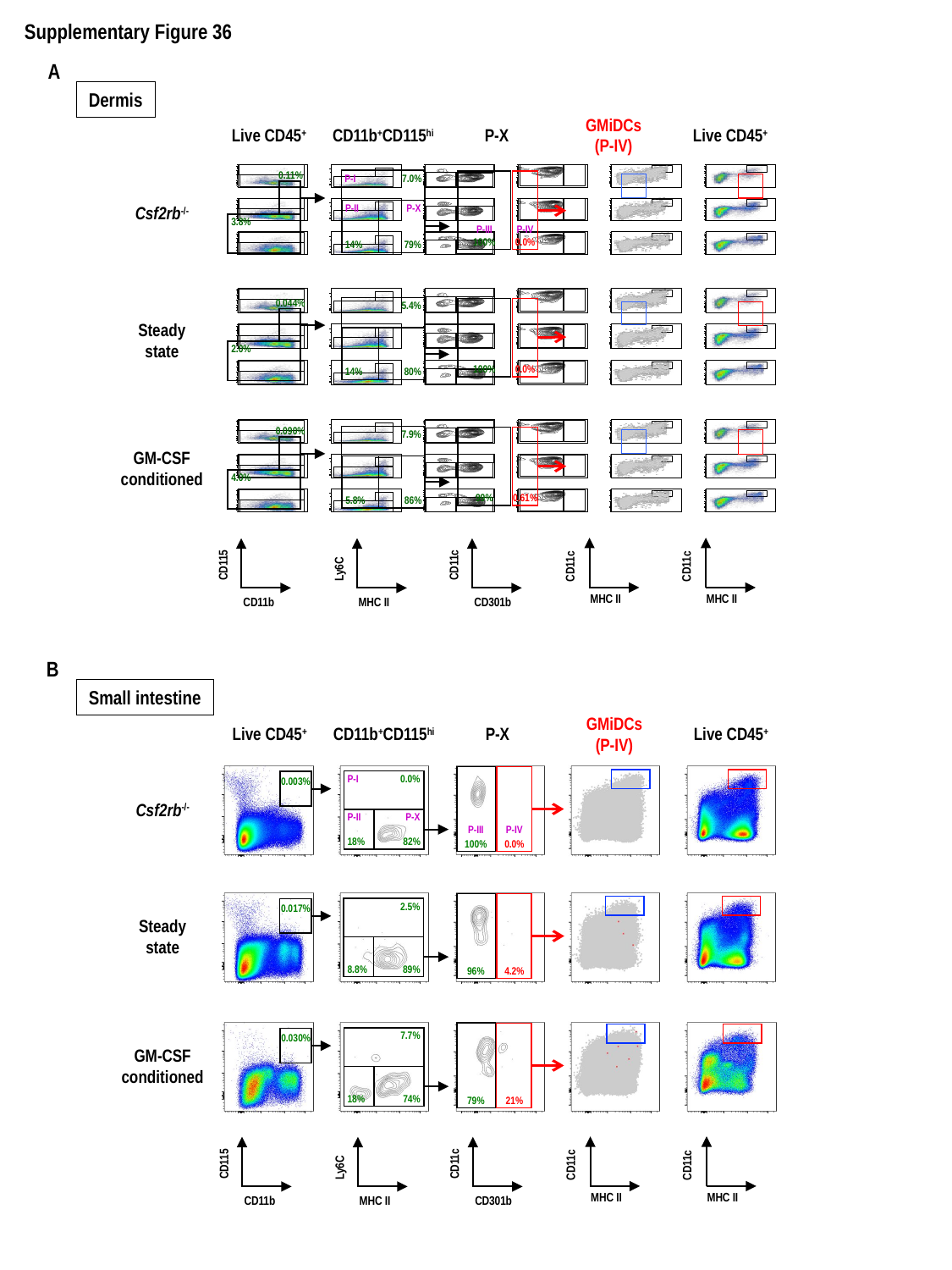

Supplementary Figure 36
A
Dermis
GMiDCs
(P-IV)
Live CD45+
CD11b+CD115hi
P-X
Live CD45+
0.11%
P-I
7.0%
P-II
P-X
3.8%
P-III
P-IV
100%
0.0%
79%
14%
Csf2rb-/-
0.044%
5.4%
2.0%
0.0%
100%
80%
14%
Steady
state
0.090%
7.9%
4.0%
99%
0.61%
86%
5.8%
GM-CSF
conditioned
CD11c
MHC II
CD11c
MHC II
CD115
CD11b
Ly6C
MHC II
CD11c
CD301b
B
Small intestine
GMiDCs
(P-IV)
Live CD45+
CD11b+CD115hi
P-X
Live CD45+
0.0%
P-I
0.003%
Csf2rb-/-
P-II
P-X
P-III
P-IV
18%
82%
100%
0.0%
2.5%
0.017%
Steady
state
89%
8.8%
96%
4.2%
7.7%
0.030%
GM-CSF
conditioned
74%
18%
79%
21%
CD115
CD11c
CD11c
CD11c
Ly6C
MHC II
MHC II
CD11b
MHC II
CD301b

## Slide 41
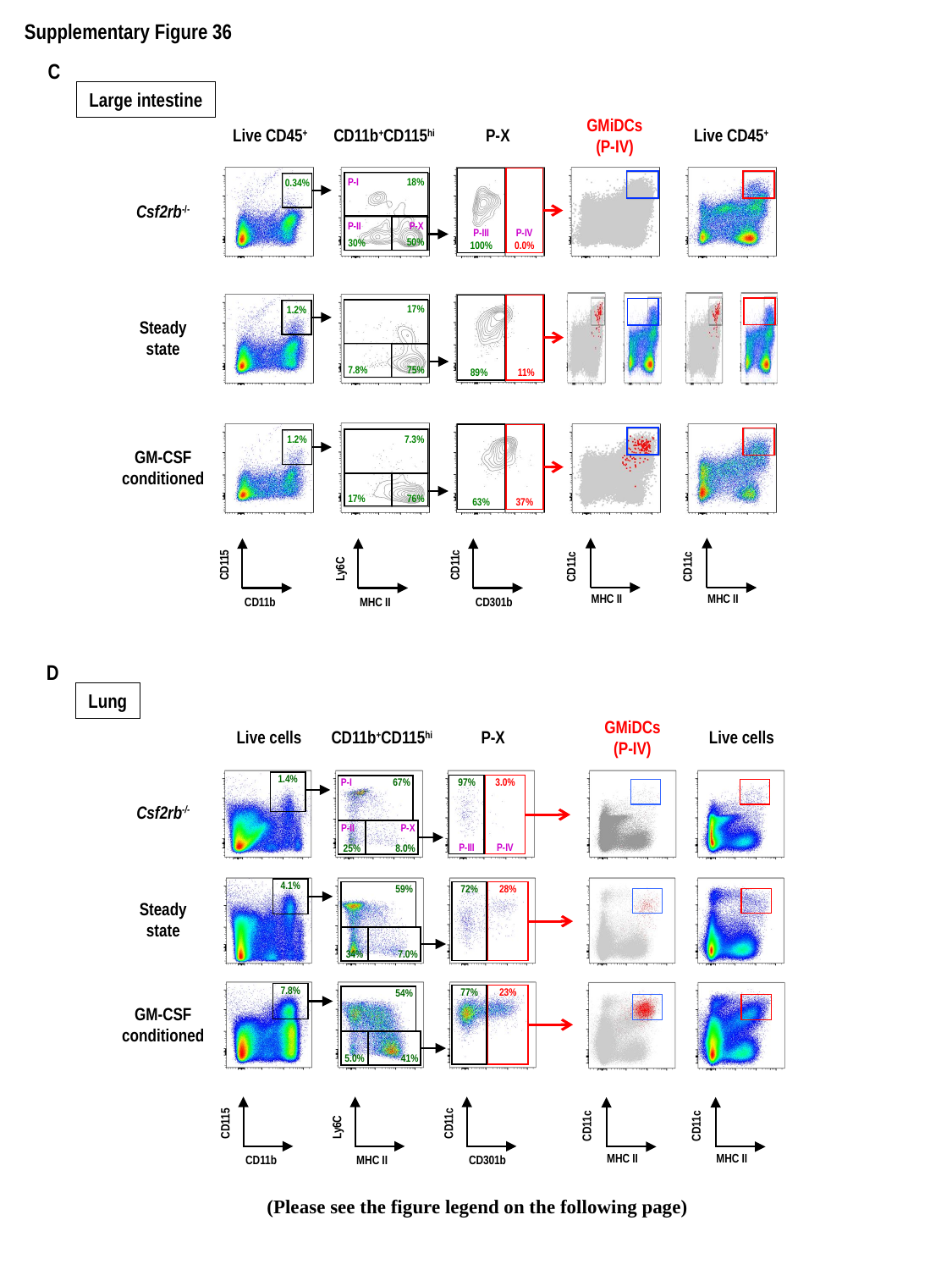

Supplementary Figure 36
C
Large intestine
GMiDCs
(P-IV)
Live CD45+
CD11b+CD115hi
P-X
Live CD45+
0.34%
18%
50%
30%
P-III
P-IV
100%
0.0%
P-I
Csf2rb-/-
P-II
P-X
1.2%
89%
11%
17%
7.8%
75%
Steady
state
7.3%
76%
17%
1.2%
63%
37%
GM-CSF
conditioned
CD11c
MHC II
CD11c
MHC II
CD115
CD11b
Ly6C
MHC II
CD11c
CD301b
D
Lung
GMiDCs
(P-IV)
Live cells
CD11b+CD115hi
P-X
Live cells
1.4%
97%
3.0%
67%
P-I
Csf2rb-/-
P-X
P-II
P-III
P-IV
25%
8.0%
4.1%
59%
72%
28%
Steady
state
34%
7.0%
7.8%
77%
23%
54%
GM-CSF
conditioned
5.0%
41%
CD115
CD11b
Ly6C
MHC II
CD11c
CD301b
CD11c
MHC II
CD11c
MHC II
(Please see the figure legend on the following page)

## Slide 42
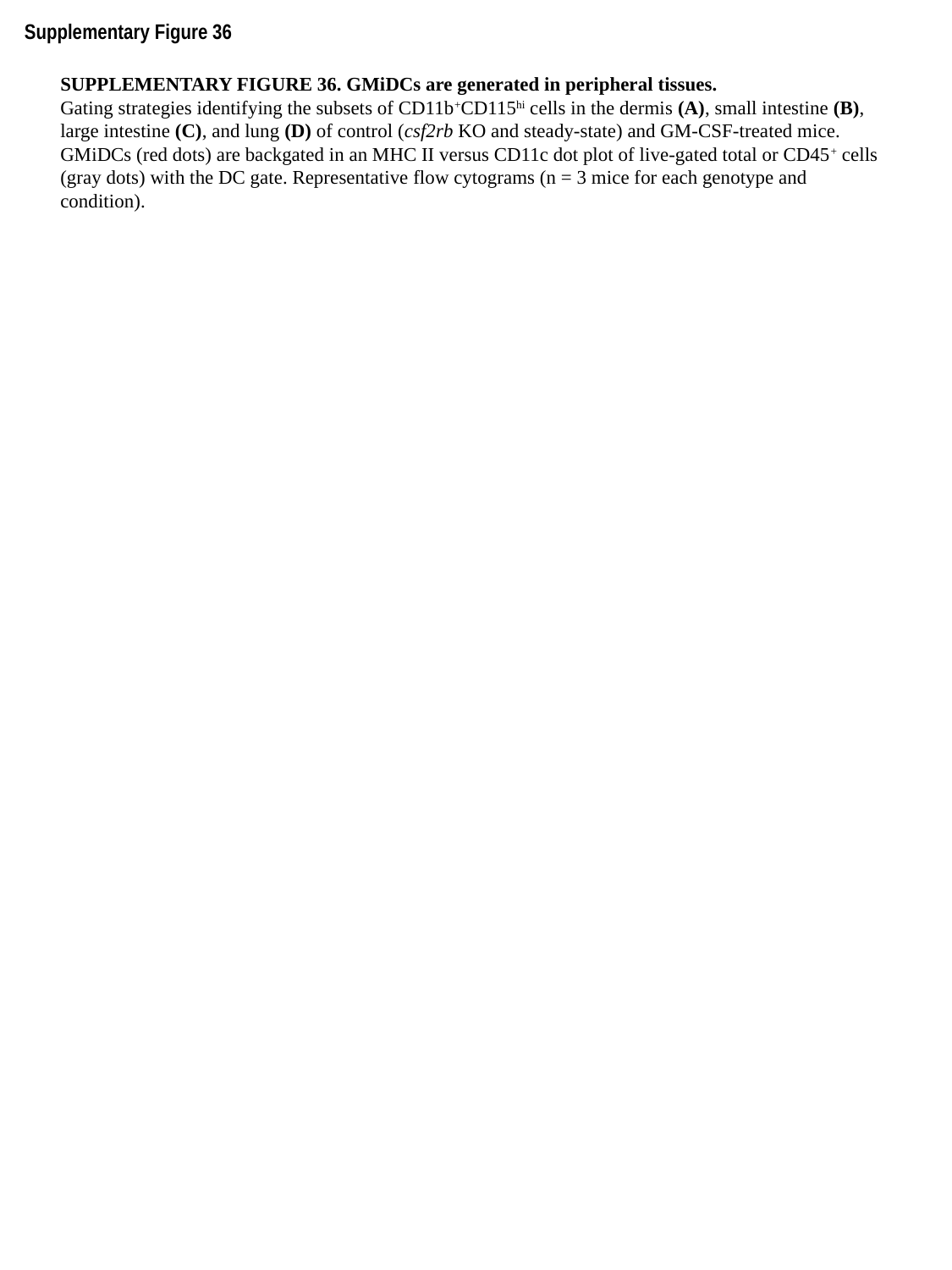

Supplementary Figure 36
SUPPLEMENTARY FIGURE 36. GMiDCs are generated in peripheral tissues.
Gating strategies identifying the subsets of CD11b+CD115hi cells in the dermis (A), small intestine (B), large intestine (C), and lung (D) of control (csf2rb KO and steady-state) and GM-CSF-treated mice. GMiDCs (red dots) are backgated in an MHC II versus CD11c dot plot of live-gated total or CD45+ cells (gray dots) with the DC gate. Representative flow cytograms (n = 3 mice for each genotype and condition).

## Slide 43
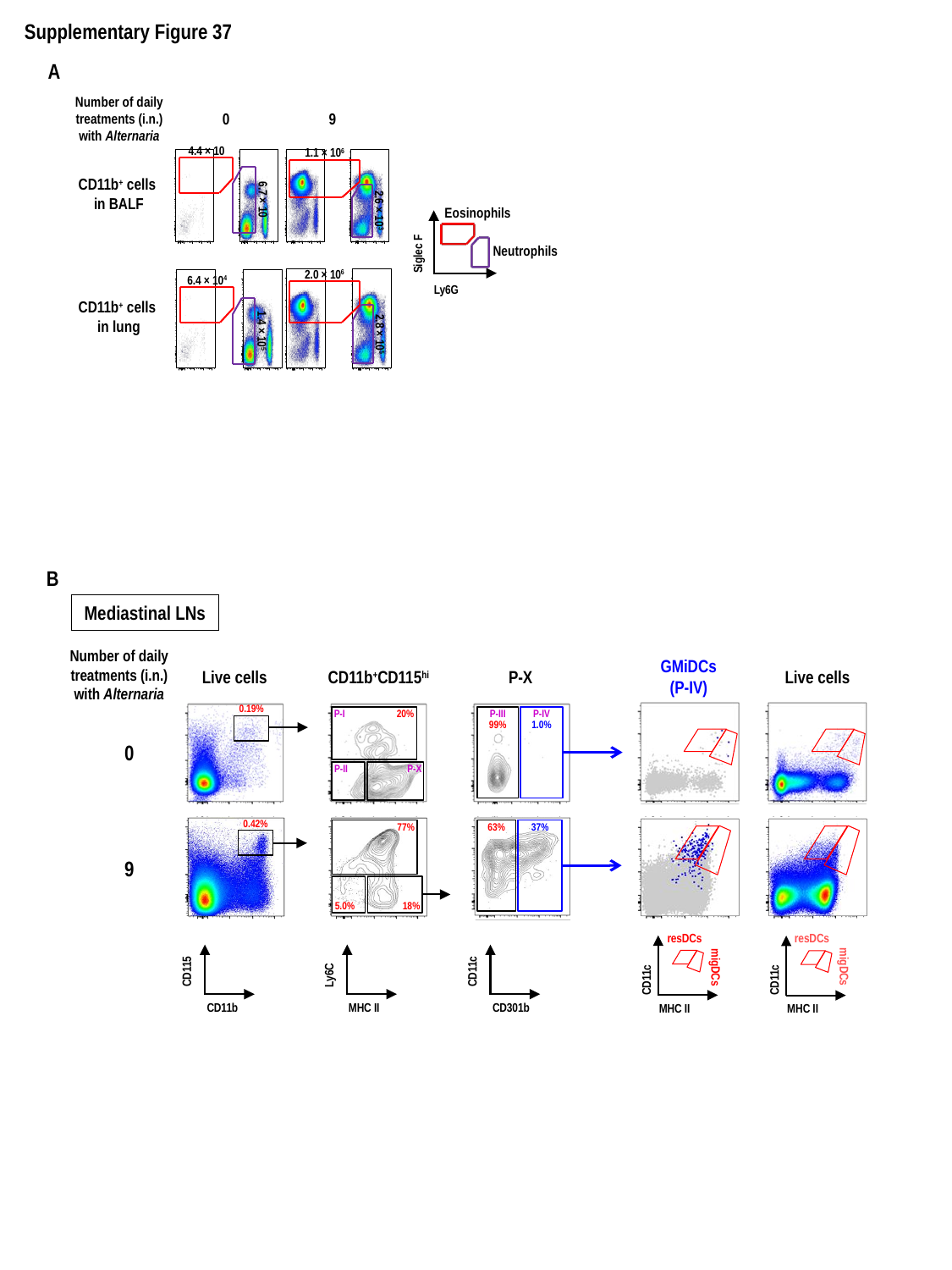

Supplementary Figure 37
A
Number of daily
treatments (i.n.)
with Alternaria
0
4.4 × 10
6.7 × 10
6.4 × 10^4^
1.4 × 10^5^
9
1.1 × 10^6^
2.6 × 10^3^
2.0 × 10^6^
2.8 × 10^5^
CD11b+ cells
in BALF
Eosinophils
Neutrophils
Siglec F
Ly6G
CD11b+ cells
in lung
B
Mediastinal LNs
Number of daily
treatments (i.n.)
with Alternaria
GMiDCs
(P-IV)
Live cells
CD11b+CD115hi
P-X
Live cells
0.19%
P-III
P-IV
20%
P-I
99%
1.0%
0
P-II
P-X
0.42%
63%
37%
77%
9
5.0%
18%
resDCs
migDCs
CD11c
MHC II
resDCs
migDCs
CD11c
MHC II
CD115
CD11b
Ly6C
MHC II
CD11c
CD301b

## Slide 44
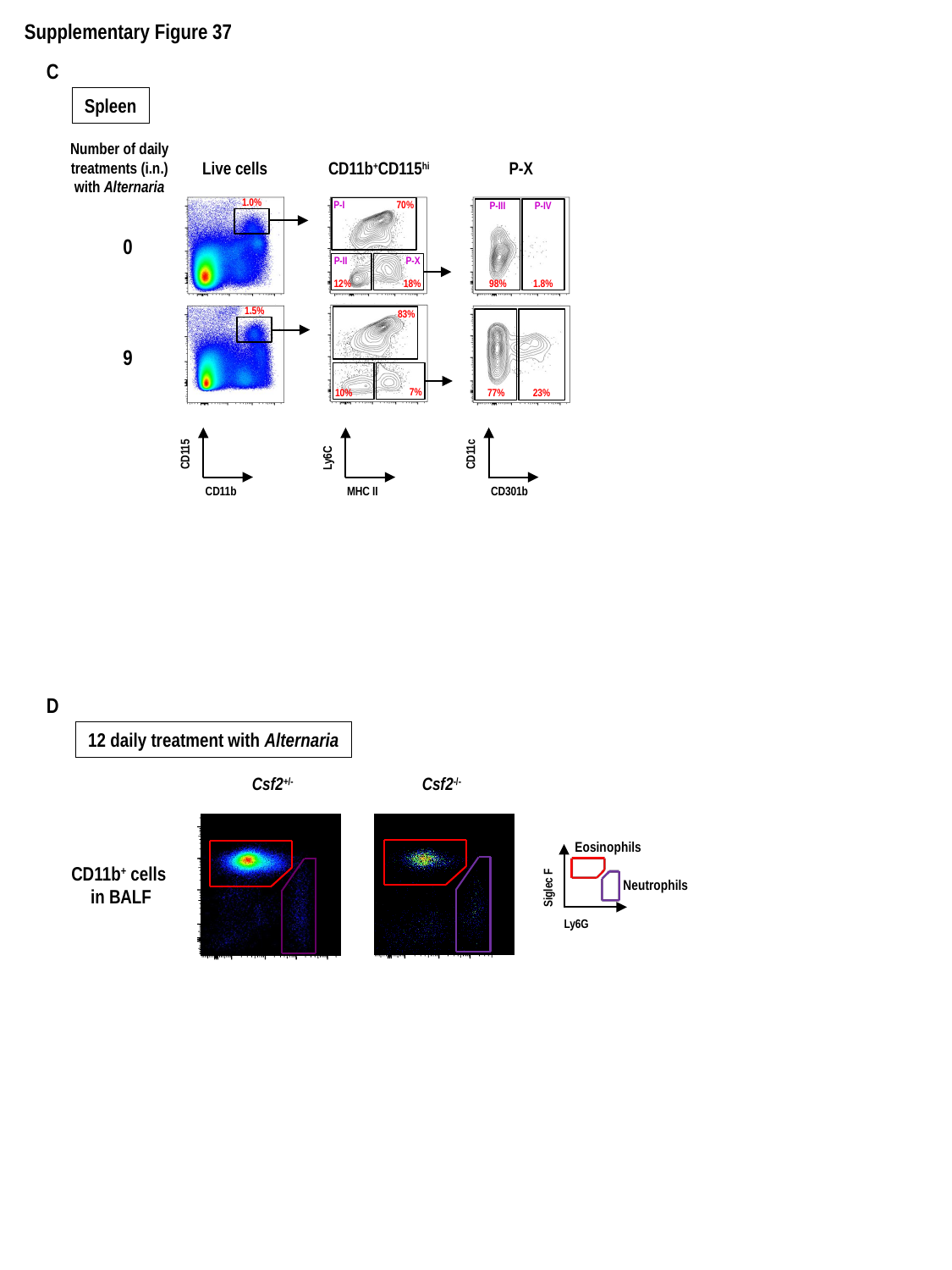

Supplementary Figure 37
C
Spleen
Number of daily
treatments (i.n.)
with Alternaria
Live cells
CD11b+CD115hi
P-X
1.0%
70%
P-I
P-III
P-IV
0
P-II
P-X
18%
12%
98%
1.8%
1.5%
83%
9
7%
10%
77%
23%
CD115
CD11b
Ly6C
MHC II
CD11c
CD301b
D
12 daily treatment with Alternaria
Csf2-/-
Csf2+/-
7.4 × 10^4^
1.0 × 10^4^
4.5 × 10^6^
6.6 × 10^4^
Eosinophils
Neutrophils
Siglec F
Ly6G
CD11b+ cells
in BALF

## Slide 45
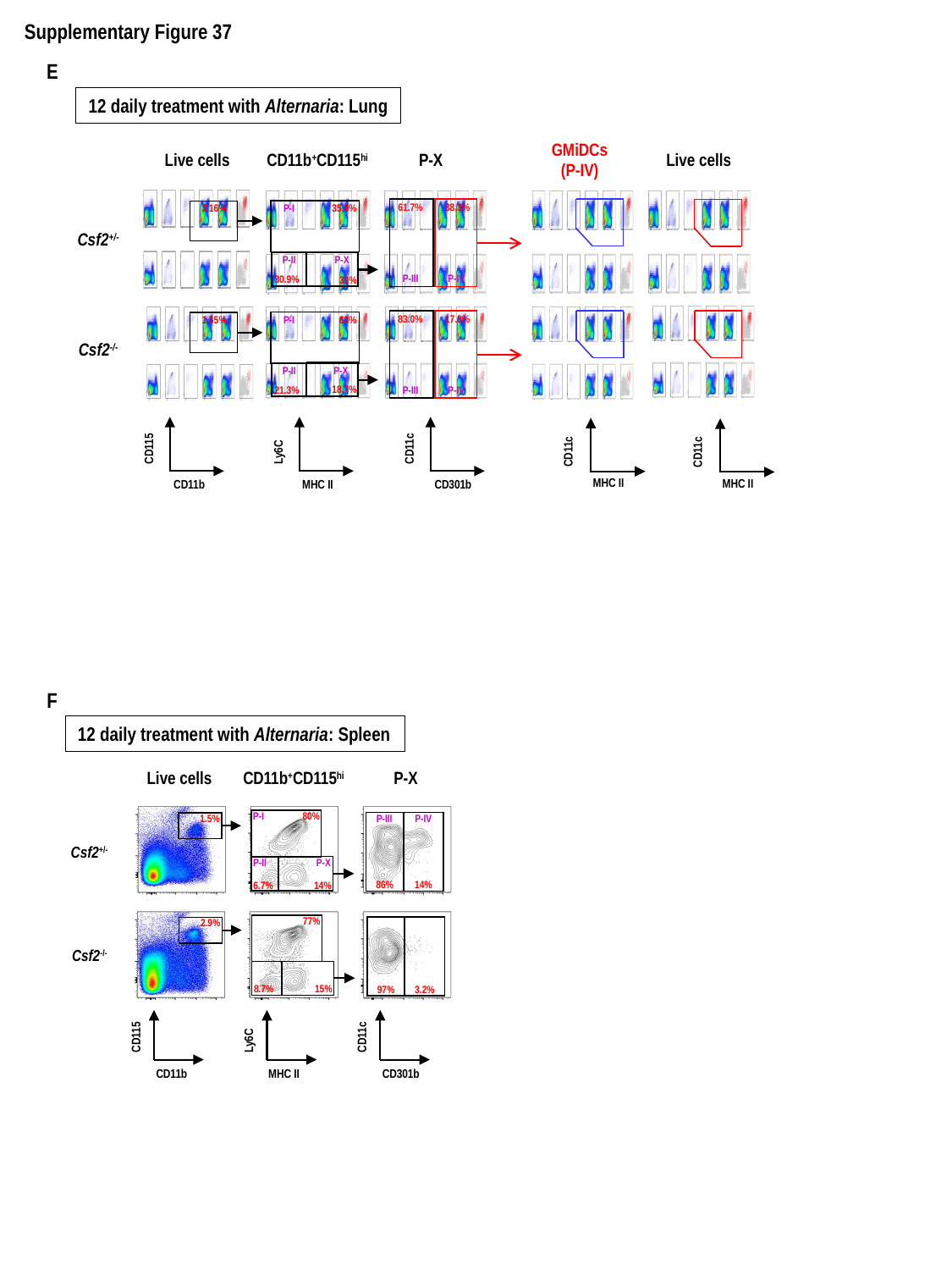

Supplementary Figure 37
E
12 daily treatment with Alternaria: Lung
GMiDCs
(P-IV)
Live cells
CD11b+CD115hi
P-X
Live cells
61.7%
38.3%
35.9%
P-I
3.16%
Csf2+/-
P-II
P-X
P-III
P-IV
30.9%
39%
83.0%
17.0%
60%
P-I
1.55%
Csf2-/-
P-II
P-X
18.7%
21.3%
P-III
P-IV
CD115
CD11b
Ly6C
MHC II
CD11c
CD301b
CD11c
MHC II
CD11c
MHC II
F
12 daily treatment with Alternaria: Spleen
Live cells
CD11b+CD115hi
P-X
80%
P-I
P-III
P-IV
1.5%
Csf2+/-
P-X
P-II
86%
14%
14%
6.7%
77%
2.9%
Csf2-/-
8.7%
15%
97%
3.2%
CD115
CD11b
Ly6C
MHC II
CD11c
CD301b

## Slide 46
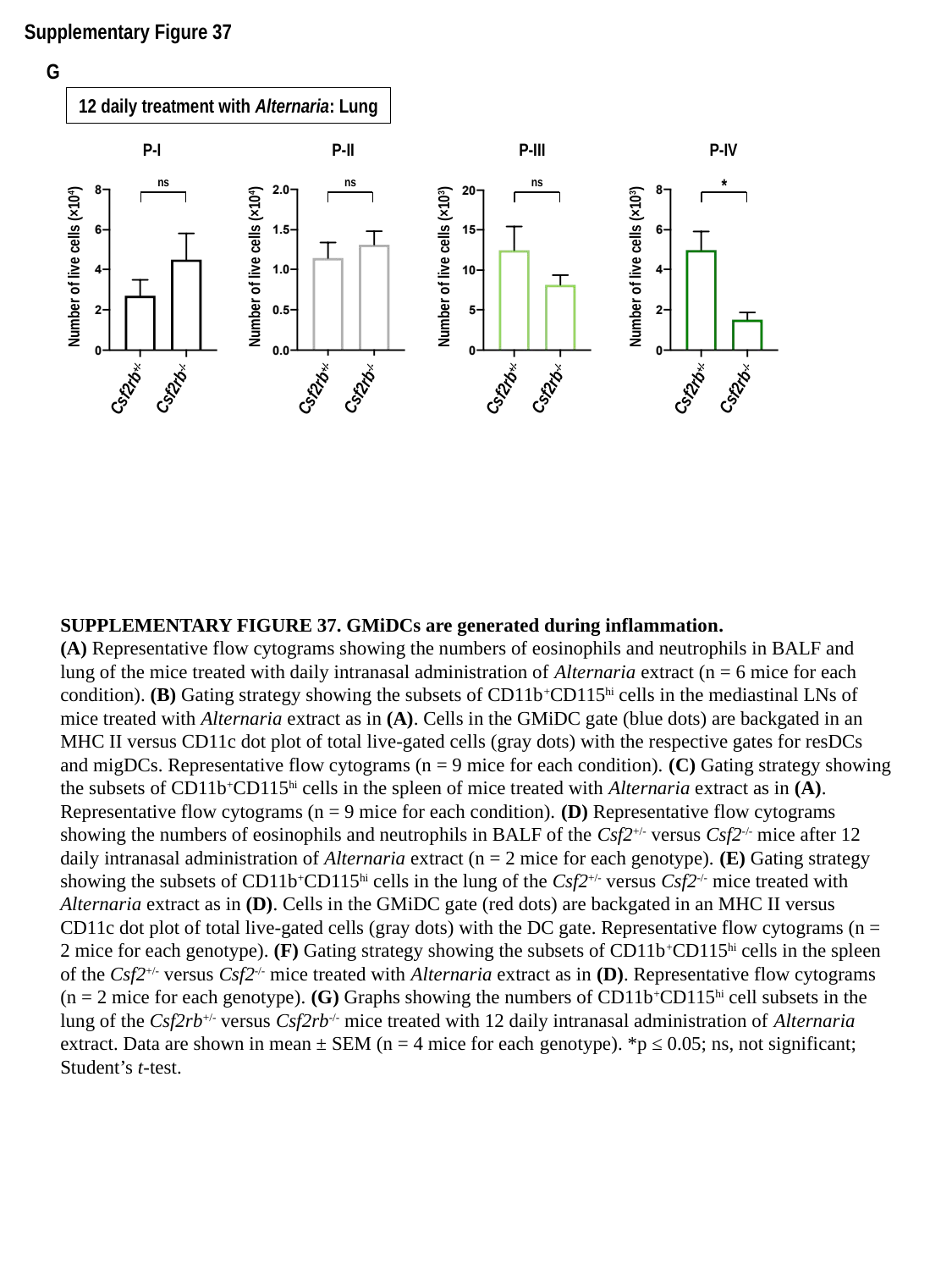

Supplementary Figure 37
G
12 daily treatment with Alternaria: Lung
P-I
P-II
P-III
P-IV
*
ns
ns
ns
Number of live cells (×104)
Number of live cells (×104)
Number of live cells (×103)
Number of live cells (×103)
Csf2rb+/-
Csf2rb-/-
Csf2rb+/-
Csf2rb-/-
Csf2rb+/-
Csf2rb-/-
Csf2rb+/-
Csf2rb-/-
SUPPLEMENTARY FIGURE 37. GMiDCs are generated during inflammation.
(A) Representative flow cytograms showing the numbers of eosinophils and neutrophils in BALF and lung of the mice treated with daily intranasal administration of Alternaria extract (n = 6 mice for each condition). (B) Gating strategy showing the subsets of CD11b+CD115hi cells in the mediastinal LNs of mice treated with Alternaria extract as in (A). Cells in the GMiDC gate (blue dots) are backgated in an MHC II versus CD11c dot plot of total live-gated cells (gray dots) with the respective gates for resDCs and migDCs. Representative flow cytograms (n = 9 mice for each condition). (C) Gating strategy showing the subsets of CD11b+CD115hi cells in the spleen of mice treated with Alternaria extract as in (A). Representative flow cytograms (n = 9 mice for each condition). (D) Representative flow cytograms showing the numbers of eosinophils and neutrophils in BALF of the Csf2+/- versus Csf2-/- mice after 12 daily intranasal administration of Alternaria extract (n = 2 mice for each genotype). (E) Gating strategy showing the subsets of CD11b+CD115hi cells in the lung of the Csf2+/- versus Csf2-/- mice treated with Alternaria extract as in (D). Cells in the GMiDC gate (red dots) are backgated in an MHC II versus CD11c dot plot of total live-gated cells (gray dots) with the DC gate. Representative flow cytograms (n = 2 mice for each genotype). (F) Gating strategy showing the subsets of CD11b+CD115hi cells in the spleen of the Csf2+/- versus Csf2-/- mice treated with Alternaria extract as in (D). Representative flow cytograms (n = 2 mice for each genotype). (G) Graphs showing the numbers of CD11b+CD115hi cell subsets in the lung of the Csf2rb+/- versus Csf2rb-/- mice treated with 12 daily intranasal administration of Alternaria extract. Data are shown in mean ± SEM (n = 4 mice for each genotype). *p ≤ 0.05; ns, not significant; Student’s t-test.
