# Supplementary material for: Granulocyte Macrophage-Colony Stimulating Factor Produces a Splenic Subset of Monocyte-Derived Dendritic Cells That Efficiently Polarize T Helper Type 2 Cells in Response to Blood-Borne Antigen
Source: Front Immunol. 2022 Jan 3;12:767037. doi: 10.3389/fimmu.2021.767037 (PMC8778578; doi:10.3389/fimmu.2021.767037)
Supplement: Supplementary file 2 [file Table_1.docx]

**SUPPLEMENTARY TABLE 1. Antibodies used in the study.**

| **Antibodies** | **Source** | **Identifier** |
| --- | --- | --- |
| anti-mouse CD3 PE/Cy7 (clone 17A2) | Biolegend | Cat # 100219 |
| anti-mouse CD3 APC (clone 145-2C11) | Biolegend | Cat # 100311 |
| anti-mouse TCRβ chain PE/Cy7 (clone H57-597) | Biolegend | Cat # 109221 |
| anti-mouse CD4 PE, PE/Cy7 (clone GK1.5) | Biolegend | Cat # 100408, 100421 |
| anti-mouse Vα2 APC, PerCP/Cy5.5 (clone B20.1) | Biolegend | Cat # 127810, 127814 |
| anti-mouse CD8a PE, PE/Cy7 (clone 53-6.7) | Biolegend | Cat # 100708 |
| anti-mouse/human CD11b APC, PerCP/Cy5.5, BV421, BV605, PE/Cy7 (clone M1/70) | Biolegend | Cat # 101211, 101228, 101235, 101237, 101216 |
| anti-mouse CD11c PE, BV421, APC/Cy7 (clone N418) | Biolegend | Cat # 117308, 117330, 117323 |
| anti-mouse CD14 PE, PE/Cy7, FITC (clone Sa14-2) | Biolegend | Cat # 123309, 123316, 123307 |
| anti-mouse CD16/32 PerCP/Cy5.5 (clone 93) | Biolegend | Cat # 101324 |
| anti-mouse CD19 PE/Cy7, APC/Cy7 (clone 6D5) | Biolegend | Cat # 115520, 115529 |
| anti-mouse CD24 PerCP/Cy5.5 (clone M1/69) | Biolegend | Cat # 101824 |
| anti-mouse CD25 PerCP/Cy5.5 (clone PC61) | Biolegend | Cat # 102030 |
| anti-mouse CD45 PE/Cy7 (clone 30-F11) | Biolegend | Cat # 103114 |
| anti-mouse CD45.1 PE, BV421 (clone A20) | Biolegend | Cat # 110707, 110731 |
| anti-mouse CD45.2 A488, A700 (clone 104) | Biolegend | Cat # 109816, 109822 |
| anti-mouse CD62L A488 (clone MEL-14) | Biolegend | Cat # 104419 |
| anti-mouse CD64 PE, PerCP/Cy5.5 (clone X54-5/7.1) | Biolegend | Cat # 139303, 139307 |
| anti-mouse CD80 PE/Cy7 (clone 16-10A1) | Biolegend | Cat # 104733 |
| anti-mouse CD83 PE/Cy7 (clone Michel-19) | Biolegend | Cat # 121517 |
| anti-mouse CD86 PE/Cy7 (clone GL-1) | Biolegend | Cat # 105013 |
| anti-mouse CD103 PE/Cy7 (clone 2E7) | Biolegend | Cat # 121425 |
| anti-mouse CD172a FITC (clone P84) | Biolegend | Cat # 144006 |
| anti-mouse CD205 PE/Cy7 (clone NLDC-145) | Biolegend | Cat # 138209 |
| anti-mouse CD206 PE/Cy7 (clone C068C2) | Biolegend | Cat # 141720 |
| anti-mouse CD207 PE (clone eBioL31) | eBioscience, Thermo Fisher Scientific | Cat # 12-2075-80 |
| anti-mouse CD209a APC, A488 (clone MMD3) | Invitrogen, Thermo Fisher Scientific | Cat # 50-2094-80, 53-2094-80 |
| anti-mouse CD209b APC (clone eBio22D1) | Invitrogen, Thermo Fisher Scientific | Cat # 17-2093-80 |
| anti-mouse CD301a PE/Cy7 (clone LOM-8.7) | Biolegend | Cat # 145609 |
| anti-mouse CD301b PE/Cy7, APC, A594 (clone URA-1) | Biolegend | Cat # 146808, 146813, 146812 |
| anti-mouse CD326 BV605 (clone G8.8) | Biolegend | Cat # 118227 |
| anti-mouse/rat XCR1 APC, PerCP/Cy5.5, FITC (clone ZET) | Biolegend | Cat # 148206, 148207, 148209 |
| anti-mouse 33D1 PE, APC (clone 33D1) | Biolegend | Cat # 124905, 124913 |
| anti-mouse Ly-6C A647 (clone HK1.4) | Biolegend | Cat # 128010 |
| anti-mouse Ly-6G PE/Cy7, PerCP/Cy5.5 (clone 1A8) | Biolegend | Cat # 127618, 127616 |
| anti-mouse F4/80 PE, PE/Cy7, PerCP/Cy5.5 (clone BM8) | Biolegend | Cat # 123109, 123127 |
| anti-mouse FcεRIα PE (clone MAR-1) | Biolegend | Cat # 134307 |
| anti-mouse H-2 PE (clone M1/42) | Biolegend | Cat # 125506 |
| anti-mouse I-A/I-E APC/Cy7, A488 (clone M5/114.15.2) | Biolegend | Cat # 107628, 107616 |
| anti-mouse PD-L1 PE/Cy7 (clone 10F.9G2) | Biolegend | Cat #124313 |
| anti-mouse PD-L2 PE/Cy7 (clone TY25) | Biolegend | Cat # 107213 |
| anti-mouse/rat CCR2 APC (clone Monoclonal Rat IgG2B Clone # 475301) | R&D systems | Cat # FAB5538A |
| anti-mouse CX3CR1 A647 (clone SA011F11) | Biolegend | Cat # 149003 |
| anti-mouse Siglec-F A647 (clone E50-2440) | BD Bioscience | Cat # 562680 |
| anti-mouse IL-4 PE/Cy7 (clone 11B11) | Biolegend | Cat # 504117 |
| anti-mouse IL-17A APC/Cy7 (clone TC11-18H10.1) | Biolegend | Cat # 506940 |
| anti-mouse IFN-γ PerCP/Cy5.5 (clone XMG1.2) | Biolegend | Cat # 505822 |
| Rat IgG1, κ isotype Ctrl PE/Cy7, APC, BV421, APC/Cy7, PerCP/Cy5.5 (clone RTK2071) | Biolegend | Cat # 400416, 400411, 400429, 400422, 400425 |
| anti-mouse CD115 biotin (clone AFS98) | Biolegend | Cat # 135508 |
| anti-mouse CD135 biotin (clone A2F10) | Biolegend | Cat # 135308 |
| anti-mouse CD11b biotin (clone M1/70) | Biolegend | Cat # 101204 |
| anti-mouse CD19 biotin (clone 6D5) | Biolegend | Cat # 115504 |
| anti-mouse CD25 biotin (clone PC61) | Biolegend | Cat # 102004 |
| anti-mouse CD44 biotin (clone IM7) | Biolegend | Cat # 103004 |
| anti-mouse NK1.1 biotin (clone PK136) | Biolegend | Cat # 108704 |
| anti-mouse I-A/I-E biotin (clone M5/114.15.2) | Biolegend | Cat # 107604 |
| anti-mouse F4/80 biotin (clone BM8) | Biolegend | Cat # 123106 |
| anti-mouse CD4 biotin (clone GK1.5) | Biolegend | Cat # 100404 |
| anti-mouse CD8 biotin (clone 53-6.7) | Biolegend | Cat # 100704 |
| anti-mouse 33D1 biotin (clone 33D1) | Biolegend | Cat # 124904 |
| anti-mouse DEC205 biotin (clone NLDC145) | Biolegend | Cat # 138212 |
| Ultra-LEAF^TM^ Purified anti-mouse IFN-γ (clone XMG1.2) | Biolegend | Cat # 505834 |
| Ultra-LEAF^TM^ Purified anti-mouse IL-4 (clone 11B11) | Biolegend | Cat # 504122 |
| anti-mouse CD209b APC (clone 22D1) | Invitrogen, Thermo Fisher Scientific | Cat # 17-2093-80 |
| anti-mouse CD169 efluor^®^ 660 (clone Siglec-1) | Invitrogen, Thermo Fisher Scientific | Cat # 50-5755-80 |
| anti-mouse B220 APC, A488 (clone RA3-6B2) | Biolegend | Cat # 103212, 103225 |
| Streptavidin PE, A488 | Biolegend | Cat # 405245, 405235 |
| anti-mouse 2A1 A647 | In house | N/A |
